# Evaluation of Arabic-Language AI Chatbot Responses to Migraine-Related Questions: A Comparative Cross-Sectional Study

**DOI:** 10.3390/jcm15103908

**Published:** 2026-05-19

**Authors:** Danah Aljaafari, Hussain Khalifa Aljumah, Mujtaba Abbas Alzuwayr, Yaqeen Mohammed Al-Essa, Hassan Ali Alradhi, Majed M. Alabdali, Nora Almuslim, Mustafa Ahmed Alqarni, Walid Alesefir

**Affiliations:** 1 Department of Neurology, College of Medicine, Imam Abdulrahman bin Faisal University, Dammam 34212, Saudi Arabia; dtaljaafari@iau.edu.sa (D.A.); nmuslim@iau.edu.sa (N.A.); mqarni@iau.edu.sa (M.A.A.); 2 College of Medicine, King Faisal University, Al-Ahsa 36362, Saudi Arabia222414184@student.kfu.edu.sa (M.A.A.); 223044014@student.kfu.edu.sa (Y.M.A.-E.); 222418924@student.kfu.edu.sa (H.A.A.); 3 Internal Medicine Department, Hail University, Hail 55476, Saudi Arabia; w.alesefyr@uoh.edu.sa

**Keywords:** artificial intelligence, AI chatbot, migraine, patient education, Arabic language, large language model, DISCERN, quality assessment

## Abstract

**Background/Objectives:** Migraine is a common and disabling neurological disorder, and many individuals increasingly seek information online. With the growing use of large language models (LLMs), such as ChatGPT, for patient education, concerns have emerged regarding the quality and reliability of the responses they generate, particularly in Arabic, where evidence remains limited. This study aimed to evaluate the reliability, quality, and accuracy of Arabic-language responses to frequently asked questions (FAQs) about migraine. **Methods:** A total of 25 FAQs were selected using a multisource approach and entered into four LLMs (ChatGPT-4.1, Gemini 3 Flash, DeepSeek-V3.2, and Grok 4.1), generating 100 responses. Responses were evaluated by a panel of expert neurologists using the modified DISCERN (mDISCERN), Global Quality Scale (GQS), and an accuracy scale. Inter-rater reliability was assessed using the intraclass correlation coefficient (ICC). **Results:** Significant differences were observed between chatbots for mDISCERN and GQS (both *p* < 0.001), whereas accuracy did not differ significantly across models (*p* = 0.072). DeepSeek and Grok demonstrated the highest mDISCERN scores (34.07 ± 1.31 and 34.29 ± 2.59, respectively), while DeepSeek achieved the highest GQS (4.95 ± 0.13). The clearest between-model differences were observed in source transparency and communication of uncertainty. Inter-rater reliability was good across all instruments (ICC range, 0.799–0.831). **Conclusions:** Medical content generated by the chatbots was broadly comparable, whereas important differences were observed in how that content was communicated. These tools may support patient education; however, their use should remain guided by clinical oversight and professional judgment.

## 1. Introduction

### 1.1. Background

Migraine is the most common primary headache disorder in adults and the second leading cause of disability worldwide, affecting approximately 15.2% of the global population [1,2]. The International Headache Society (IHS) defines migraine as recurrent headache attacks typically accompanied by nausea, vomiting, photophobia, and phonophobia [3]. Patients typically experience these attacks in distinct clinical phases, which can include prodromal symptoms, an aura phase for some individuals, the primary headache, and a postdromal recovery period. Migraine substantially affects multiple domains of patients’ lives, including social, occupational, and family functioning [4]. Despite its widespread prevalence and significant burden, migraine remains substantially underdiagnosed and undertreated worldwide [5].

Many individuals with migraine do not seek medical care or fail to receive an accurate diagnosis and guideline-recommended treatment. This gap is partly driven by cultural misconceptions that frame migraine as psychosocial rather than a neurological disorder, thereby limiting access to appropriate care [6,7]. Consequently, patients with headache disorders often turn to informal yet readily accessible sources of health information, including the internet and social media [8], reflecting a broader shift toward digital platforms for health-related inquiries.

### 1.2. Emergence of AI Chatbots

In recent years, artificial intelligence (AI) has gained increasing attention in healthcare [9]. Large language models (LLMs) are advanced systems trained on extensive datasets from diverse sources across the internet. These systems have progressed quickly from basic rule-based search tools to highly capable, transformer-based generative models. Building on this foundation, AI chatbots can provide rapid, text-based responses to health-related queries and support patient education [10,11]. Chatbots such as ChatGPT, Google Gemini, DeepSeek, and Grok have experienced widespread public adoption because they allow direct, conversational interaction with users. However, the quality and reliability of their responses remain variable across clinical topics and platforms [12,13,14]. Although these systems may provide accurate and comprehensive responses, important limitations persist [15], including factual inaccuracies, “hallucinations,” and completely fabricated citations. These issues can easily mislead users and negatively influence their health decisions.

### 1.3. Rationale

Addressing these limitations through culturally adapted, AI-driven patient education tools may improve access to reliable information and support patient engagement in migraine management. Although benchmarking studies of LLMs for migraine education have recently emerged [16,17,18], rigorous evaluations remain limited, particularly for Arabic-language outputs. Previous studies have reported lower performance of LLMs in non-English contexts, including Arabic [19,20]. This is particularly relevant in Arabic-speaking populations, where migraine is frequently underdiagnosed and associated with a significant disability [21]. Therefore, this study aimed to evaluate and compare the reliability, quality, and accuracy of Arabic-language responses generated by four contemporary LLMs (ChatGPT-4.1, Gemini 3 Flash, DeepSeek-V3.2, and Grok 4.1) to frequently asked questions (FAQs) about migraine. The specific objectives were: (i) to quantify reliability using the modified DISCERN instrument (mDISCERN); (ii) to evaluate overall informational quality using the Global Quality Scale (GQS); (iii) to assess scientific accuracy against established headache guidelines; and (iv) to characterize between-model differences in source transparency and communication of uncertainty.

## 2. Materials and Methods

This cross-sectional study was conducted between 25 December 2025 and 20 February 2026 to evaluate the quality, reliability, and accuracy of Arabic-language responses generated by four major LLMs (ChatGPT-4.1, Gemini 3 Flash, DeepSeek-V3.2, and Grok 4.1). Using a standardized set of 25 FAQs about migraine, a panel of four blinded raters assessed the chatbot outputs using three validated evaluation instruments: the mDISCERN tool, the GQS, and an accuracy scale.

### 2.1. Study Design and Question Selection

**Figure 1:**
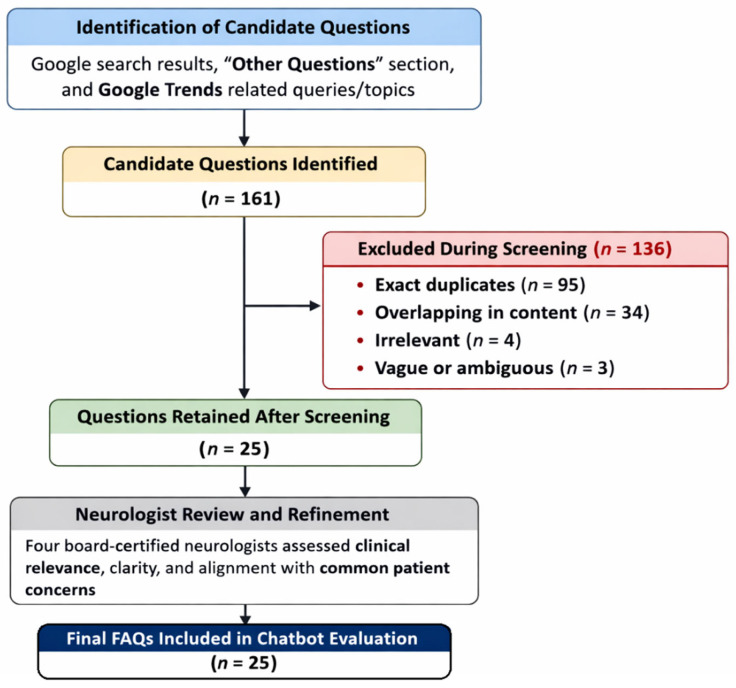
Flowchart regarding the retrieval of study questions.

A systematic, multi-source approach was employed to curate the question set to reflect real-world patient information-seeking behavior (Figure 1). An initial search was conducted using the Google Chrome browser in incognito mode with cleared cookies and cache to minimize personalized search bias. The search used the common Arabic term for migraine, Al-Shaqiqa (الشقيقة). Only websites appearing on the first three pages of the search engine results pages (SERPs) were analyzed, as most users do not proceed beyond these pages [22]. The “Other Questions” section was also reviewed to identify additional queries. To capture public interest trends, Google Trends searches from the past five years using the “worldwide” filter were conducted with the same Arabic term to identify top and rising related queries and topics. We then pooled all candidate queries and excluded exact duplicates, overlapping content, and irrelevant or ambiguous questions. Finally, a panel of board-certified neurologists reviewed the remaining items for clinical relevance, clarity, and alignment with common patient concerns. This process yielded the final set of 25 migraine-related FAQs for chatbot evaluation. The full set of 25 Arabic-language migraine FAQs and the 100 generated responses from the four evaluated AI chatbots are provided in Supplementary Materials Table S1.

### 2.2. Entry Procedure

Each query was processed using the latest freely accessible versions of four LLMs: ChatGPT-4.1 (OpenAI; San Francisco, CA, USA), Gemini 3 Flash (Google DeepMind; London, UK), DeepSeek-V3.2 (DeepSeek-AI; Hangzhou, China), and Grok 4.1 (xAI; Palo Alto, CA, USA). All questions were entered on 5 January 2026 by a single author who was not involved in the evaluation.

New standardized accounts were created for each platform to eliminate the influence of prior user history or personalization algorithms. Queries were entered exactly as written, without modification, clarification, or follow-up prompts. A new chat session was used for each query to minimize contextual carryover. This procedure was designed to closely simulate real-world public user behavior while maintaining methodological rigor and minimizing bias. Responses were collected and stored in Microsoft Word (Microsoft 365, version 2026; Microsoft Corporation, Redmond, WA, USA).

### 2.3. Evaluation of Responses

Quality and reliability of responses were assessed using three evaluation tools: the mDISCERN instrument, GQS, and an accuracy scale. The mDISCERN instrument is derived from the validated 16-item tool for evaluating the quality of written health information. It comprised three sections: Section 1 evaluated publication reliability through eight specific items (Items 1–8); Section 2 assessed the quality of information regarding treatment choices across seven items (Items 9–15); and Section 3 provided a single, independent overall quality rating (Item 16). Each item was scored on a 5-point Likert scale ranging from 1 (no), 2–4 (partially), to 5 (yes), yielding a possible total score of 16 to 80 [23]. The majority of the 25 included FAQs were oriented toward general patient education—addressing migraine symptoms, triggers, lifestyle considerations, and red-flag features—rather than specific treatment choices. Consequently, applying Section 2 of the DISCERN instrument, which evaluates information about treatment options, would have resulted in scores that reflected the nature of the questions rather than the performance of the chatbots. Thus, we utilized only Section 1 of the DISCERN instrument, an approach consistent with the mDISCERN approach described in the previous literature [24]. According to this approach [24], mDISCERN scores < 15 were categorized as poor, scores between 16 and 31 as fair, and scores > 32 as good.

The GQS is a validated 5-point Likert scale used to assess the overall quality and usability of information, with scores ranging from 1 (poor quality) to 5 (excellent quality and comprehensive information) [25].

Accuracy was evaluated using a 5-point Likert scale adapted from previous AI benchmarking research [26]. Accuracy was assessed with reference to established migraine definitions and diagnostic criteria from the International Classification of Headache Disorders, 3rd edition (ICHD-3) [27], as well as current treatment recommendations from the American Headache Society [28]. The scale was used to assess the scientific accuracy and currency of the responses, with scores ranging from 1 (Very inaccurate: seriously misleading, providing information contrary to current medical evidence and potentially causing harm) to 5 (Very accurate: highly accurate and up to date with current medical knowledge, requiring only minor adjustments) [26].

Each AI-generated response was independently assessed by a panel of four board-certified neurologists, each with 10–15 years of specialized clinical and academic experience in migraine and headache disorders at a tertiary academic medical center. Prior to assessment, evaluators attended a standardized calibration session in which scoring criteria and rating guidelines were reviewed to ensure consistent application of all instruments. To reduce grading bias, evaluators were instructed not to query AI chatbots with the study questions prior to formal assessment. Responses were anonymized, assigned unique identifiers, and color-coded before evaluation, ensuring that evaluators were blinded to the source platform.

Disagreements between evaluators were resolved through discussion and consensus, with adjudication applied when ratings differed by two points or more.

### 2.4. Statistical Analysis

All statistical analyses were performed using IBM SPSS Statistics for Windows, Version 29.0 (IBM Corp., Armonk, NY, USA), and R software (RStudio, Version 2023.12.0; Posit Software, PBC, Boston, MA, USA). Continuous variables were summarized as mean ± standard deviation (SD) and median with interquartile range (IQR), while categorical variables were presented as frequencies and percentages. Normality was assessed using the Shapiro–Wilk test. Differences in total mDISCERN, GQS, accuracy, and item-level mDISCERN component scores among AI chatbots were assessed using the Kruskal–Wallis test. Post hoc pairwise comparisons using Dunn’s test with Bonferroni correction for outcomes showing a significant overall difference. Inter-rater reliability among evaluators was assessed using the intraclass correlation coefficient (ICC) based on a two-way random-effects, absolute-agreement model and interpreted as poor (<0.50), moderate (0.50–0.75), good (0.75–0.90), or excellent (>0.90). Spearman correlation analysis was performed to examine associations among mDISCERN, GQS, and accuracy scores. A *p*-value < 0.05 was considered statistically significant.

## 3. Results

This study evaluated the quality of Arabic-language responses generated by ChatGPT, DeepSeek, Gemini, and Grok to 25 migraine-related patient questions. A total of 100 responses were generated and evaluated, with 25 responses per chatbot. The following sections first detail the inter-rater reliability of the evaluation instruments. Subsequent analyses compare chatbot performance at both the item and overall instrument levels. Finally, the categorical score distributions and the correlations between the three quality metrics are presented.

Table 1 presents the inter-rater reliability of overall scores assigned by four independent raters. Mean mDISCERN scores ranged from 31.60 (SD 2.37) to 32.46 (SD 3.53), with an ICC of 0.831 (*p* < 0.001), indicating good reliability. Similarly, GQS scores showed minimal variation among raters, ranging from 4.68 (SD 0.62) to 4.75 (SD 0.58), with an ICC of 0.816 (*p* < 0.001). Accuracy scores ranged from 4.68 (SD 0.57) to 4.75 (SD 0.54), with an ICC of 0.799 (*p* < 0.001), indicating good agreement across all instruments.

Having established the reliability of the scoring process, performance differences across specific item-level criteria were evaluated. Table 2 demonstrates statistically significant differences among AI chatbots for several item-level mDISCERN components. For Q4 (identification of information sources), a statistically significant difference was observed (H = 55.055, *p* < 0.001), with Grok achieving the highest mean score (3.77 ± 1.28), whereas ChatGPT (1.63 ± 0.42), DeepSeek (1.27 ± 0.76), and Gemini (1.27 ± 0.68) scored lower. For Q5 (clarity of when the information was produced), DeepSeek demonstrated the highest mean score (4.78 ± 0.17; H = 12.357, *p* < 0.001). Q6 (balanced and unbiased information) also differed significantly (H = 21.471, *p* < 0.001), with Gemini demonstrating the highest mean score (4.91 ± 0.19). Significant differences were also observed for Q7 (additional sources of support and information) (H = 50.251, *p* < 0.001), where DeepSeek performed best (4.08 ± 0.68), and for Q8 (acknowledgment of uncertainty; H = 27.587, *p* < 0.001), where DeepSeek again achieved the highest score (4.30 ± 0.54). No significant differences were identified for Q1–Q3.

**Figure 2:**
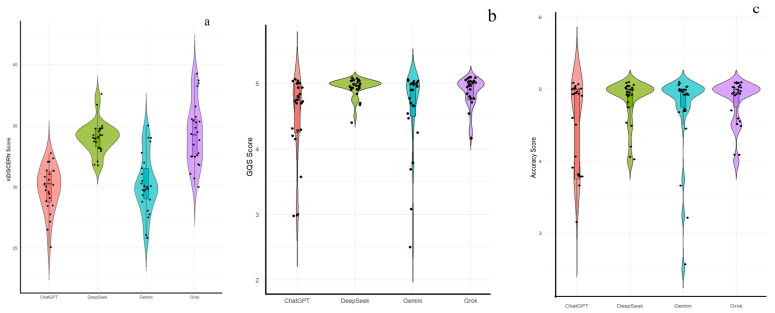
Violin plots illustrating the distribution of total mDISCERN (**a**), Global Quality Score (GQS) (**b**), and accuracy (**c**) scores across ChatGPT, DeepSeek, Gemini, and Grok. Internal box plots indicate the median and interquartile ranges, while the outer shape reflects the probability density of the scores.

Building upon these individual component differences, the overall instrument-level scores were then assessed. Table 3 compares overall mDISCERN, GQS, and accuracy scores across chatbots. Significant differences were observed in mDISCERN scores (*p* < 0.001), with DeepSeek (34.07 ± 1.31; median 34.00 [33.25–34.75]) and Grok (34.29 ± 2.59; 34.25 [32.63–35.75]) outperforming ChatGPT (29.83 ± 1.87; 30.25 [28.88–31.50]) and Gemini (30.23 ± 2.39; 30.00 [28.50–31.38]). GQS scores also differed significantly (*p* < 0.001), with DeepSeek demonstrating the highest quality (4.95 ± 0.13; 5.00 [5.00–5.00]), followed by Grok and Gemini, while ChatGPT scored comparatively lower. Accuracy scores did not differ significantly across chatbots (*p* = 0.072), although DeepSeek and Grok demonstrated slightly higher mean values than ChatGPT and Gemini. The distribution of total mDISCERN, GQS, and accuracy scores is shown in Figure 2. Figure 2a illustrates an upward shift and a narrower distribution of mDISCERN scores for DeepSeek and Grok. Figure 2b highlights that DeepSeek responses are heavily clustered at the maximum GQS value. Figure 2c displays overlapping density profiles for accuracy across all models, which aligns with the non-significant Kruskal–Wallis findings.

**Figure 3:**
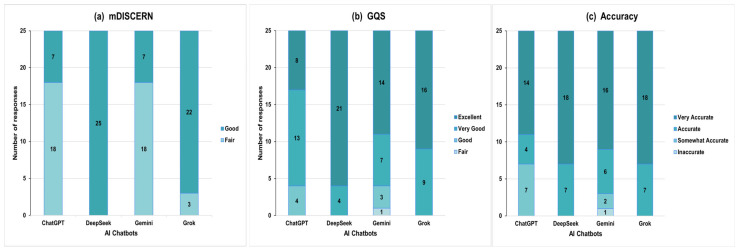
Distribution of categorical mDISCERN (**a**), GQS (**b**), and accuracy (**c**) scores across AI chatbots. Bars represent the number of responses in each category. Values displayed within the bars represent the total number of responses in each category.

To complement the analysis of continuous scores, the responses were stratified into categorical variables for distribution analysis. Figure 3a presents the distribution of mDISCERN categories across AI chatbots. ChatGPT and Gemini showed identical distributions, with 18 (72.0%) responses classified as fair and 7 (28.0%) as good. DeepSeek demonstrated the highest proportion of ‘good’ ratings, with all 25 (100%) responses classified as good, while Grok showed 22 (88.0%) good and 3 (12.0%) moderate responses. Figure 3b shows the distribution of GQS categories across AI chatbots. DeepSeek demonstrated the most favorable distribution, with 21 (84.0%) responses classified as excellent and 4 (16.0%) as very good. Grok also showed predominantly high-quality responses, with 16 (64.0%) excellent and 9 (36.0%) very good responses. Gemini showed 14 (56.0%) excellent, 7 (28.0%) very good, 3 (12.0%) good, and 1 (4.0%) fair responses, whereas ChatGPT showed 8 (32.0%) excellent, 13 (52.0%) very good, 4 (16.0%) good, and no fair responses. Figure 3c shows the distribution of accuracy categories across AI chatbots. ChatGPT showed 14 (56.0%) very accurate, 4 (16.0%) accurate, and 7 (28.0%) somewhat accurate responses, with no inaccurate responses. DeepSeek and Grok each showed 18 (72.0%) very accurate and 7 (28.0%) accurate responses, with no responses categorized as somewhat accurate or inaccurate. Gemini showed 16 (64.0%) very accurate, 6 (24.0%) accurate, 2 (8.0%) somewhat accurate, and 1 (4.0%) inaccurate responses.

Given the significant overall variance observed in the continuous mDISCERN and GQS scores, post hoc analyses were required to isolate specific between-model differences. Table 4 presents pairwise comparisons of mDISCERN and GQS scores among AI chatbots following a significant Kruskal–Wallis test. For mDISCERN, significant differences were observed between ChatGPT–Grok (Z = −5.373, *p* < 0.001), ChatGPT–DeepSeek (Z = −5.524, *p* < 0.001), Gemini–Grok (Z = −4.865, *p* < 0.001), and Gemini–DeepSeek (Z = 5.017, *p* < 0.001). For GQS, only the ChatGPT–DeepSeek comparison showed a significant difference (Z = −3.893, *p* = 0.001). Accuracy comparisons were not conducted due to a non-significant Kruskal–Wallis result.

As a final analytical step, the relationships between the three primary evaluation tools were assessed. Table 5 shows Spearman correlations among mDISCERN, GQS, and accuracy scores. mDISCERN was moderately positively correlated with both GQS (r = 0.499, *p* < 0.01) and accuracy (r = 0.412, *p* < 0.01). The strongest positive correlation was observed between GQS and accuracy (r = 0.769, *p* < 0.01), indicating that higher overall quality scores were associated with higher accuracy scores.

## 4. Discussion

### 4.1. Summary

This study contributes to the limited literature evaluating Arabic-language chatbot responses for patient education and provides condition-specific evidence in the context of migraine. To our knowledge, this study is among the first to provide a comparative evaluation of Arabic-language responses generated by four contemporary LLMs (ChatGPT, DeepSeek, Gemini, and Grok) in addressing migraine-related FAQs. Although overall performance was high across all four chatbots, statistically significant differences were observed in reliability and overall quality. DeepSeek and Grok achieved the highest mDISCERN scores, suggesting superior performance in reliability domains compared with ChatGPT and Gemini. DeepSeek also achieved the highest GQS, indicating higher overall response quality. In contrast, accuracy scores were comparable across models, indicating that the core medical content remained largely consistent despite differences in content presentation and formatting. These findings highlight the potential role of AI chatbots as adjunctive tools in enhancing migraine patient education.

### 4.2. Comparison with Existing Literature

Previous research suggests that chatbot-generated responses may provide accessible and clinically relevant information; however, their quality and reliability are not uniform across platforms [29,30,31,32]. Li et al. evaluated five LLMs (ChatGPT-3.5, ChatGPT-4.0, Google Bard, Meta Llama 2, and Sonnet 2) for migraine patient education and reported generally favorable performance across models, with ChatGPT-4.0 achieving the highest performance, although differences were not statistically significant [16]. Similarly, Garcia et al. found that ChatGPT-4o generated largely acceptable responses to migraine-related clinical questions, although concerns remained regarding source validity and citation reliability [18]. Consistent with these findings, the present study demonstrated high overall accuracy across all chatbots, with no statistically significant differences between models. However, in contrast to Li et al., notable variation was observed in reliability and overall quality, suggesting that factual accuracy alone may not fully capture the value of chatbot responses in patient education. A similar divergence between accuracy and broader informational quality has been reported in other clinical contexts. For instance, Bozgeyik et al. found no significant differences in accuracy, clarity, or consistency between ChatGPT-5 and DeepSeek R1 for meniscus-related queries; however, DeepSeek R1 performed significantly better in comprehensiveness [29]. In the present study, significant differences were observed in both mDISCERN and GQS scores. DeepSeek and Grok achieved higher than ChatGPT and Gemini, reflecting differences in how responses were framed and contextualized rather than in factual correctness. The most pronounced differences were observed in source transparency and reliability-related components, including identification of sources, clarity of information timing, balance and objectivity, provision of additional resources, and acknowledgment of uncertainty. ChatGPT and Gemini were generally comparable in clarity but less consistent in these reliability-related features. Both were weaker in source transparency, while ChatGPT was also less likely to provide additional support resources or acknowledge uncertainty. These findings align with prior literature highlighting the risks of persuasive but unreliable AI-generated medical content. Specifically, Gravel et al. demonstrated that a substantial majority of medical references generated by AI can be entirely fabricated, despite appearing deceptively authentic [33]. Garcia et al. observed similar reference-related hallucinations within migraine-specific queries, noting that the AI frequently swapped publication dates and erroneously included prominent authors to manufacture scientific credibility [18]. These observations are consistent with Zhang and Zhao, who noted that these correctly formatted but fictional citations mislead users and undermine academic rigor [34]. As Yıldız et al. emphasized, the failure of AI chatbots to provide transparent, reliable sources compromises the perceived credibility of the information and risks disseminating misleading health guidance [30]. The lack of clear source attribution raises concerns regarding trustworthiness and limits the suitability of these tools as standalone patient-education resources, particularly for lay users. In this context, acknowledgment of uncertainty and guidance toward appropriate medical consultation are important indicators of safe health communication. In this study, Grok performed better in referencing sources, whereas DeepSeek more consistently acknowledged its limitations as a chatbot, advised users to consult a neurologist, highlighted headache red flags, and clarified that its responses were general in nature and not a substitute for professional medical advice. Notably, these observations contrast with several recent evaluations across diverse medical specialties in which specific models—predominantly ChatGPT and Gemini—demonstrated superior performance. For instance, Tuzlalı et al. reported that ChatGPT-o1 generated the highest-quality and most accurate responses for dental FAQs [35], while Patel and Radcliffe observed that ChatGPT-4o demonstrated superior overall capabilities in providing oncological information [36]. Similarly, ChatGPT-4o was identified as the top-performing model for information quality by Çabuk Çelik and Altunel Kılınç in the context of rheumatology [37], while Kacer found that Gemini achieved the highest reliability scores regarding maternal health guidance [38]. This discrepancy may be attributed to differences in clinical topic, language, model version, prompt design, or evaluation criteria. Language may contribute to LLM performance, as previous studies have reported differences in chatbot output quality across languages [39,40,41,42]. For instance, Sallam et al. reported a significant disparity between Arabic and English in infectious disease queries, with English responses demonstrating consistently superior performance [41]. Furthermore, comparative bilingual evaluations in endocrine and pediatric medication-safety tasks have shown that model performance may vary across languages, reinforcing the observation that multilingual reliability remains uneven in some clinical contexts [39,42]. In contrast, this pattern may not be universal. In ophthalmology queries, Sallam et al. reported excellent performance with comparable trends in both Arabic and English [43]. These findings may suggest that language-based differences in chatbot performance do not follow a uniform trajectory; rather, they are influenced by the clinical topic, model architecture, and evaluation frameworks. While the iterative advancement of newer-generation models may reduce the linguistic gaps observed in earlier benchmarks, this does not eliminate the need for direct evaluation of Arabic-language outputs.

### 4.3. Clinical Implications

Greater reliance on LLMs raises important concerns regarding the reliability and transparency of the information they provide. This issue is clinically relevant because headache is a symptom shared by both primary disorders, such as migraine, and potentially serious secondary conditions, emphasizing the importance of recognizing red flags and appropriate qualifying information. In this context, chatbot responses that lack clear sourcing or sufficient acknowledgment of uncertainty may still appear credible while providing an incomplete basis for patient decision-making. As the use of these technologies continues to expand, more individuals may rely on AI systems for health-related information, highlighting the need to improve transparency, citation practices, and the overall reliability of AI-generated medical content. Accordingly, while AI chatbots may serve as useful tools to support patient education, their responses should be interpreted with caution and should not replace professional medical consultation.

### 4.4. Limitations and Future Directions

Several limitations should be considered when interpreting the findings of this study. First, a cross-sectional design was used, and chatbot responses were assessed at a single time point. The potential for temporal variability should be acknowledged, as the evaluated LLMs are subject to continuous refinement and iterative updates. Because responses to identical queries may fluctuate over time, this cross-sectional analysis provides a time-anchored benchmark rather than a fixed performance metric. The relative performance differences observed between the models may therefore be more informative for clinical and educational guidance than the absolute scores reported at the time of data collection. Second, the analysis was based on a limited set of 25 migraine-related questions, which were primarily general patient-oriented queries rather than complex clinical scenarios. This may have favored broadly acceptable performance and may not fully reflect how these systems perform when addressing more nuanced, ambiguous, or higher-risk questions. Third, newer model versions, paid versions, and specialized modes were not evaluated, which may limit the generalizability of the findings to other system configurations. Fourth, the study focused specifically on Arabic-language outputs, and direct comparison with English responses was not included; therefore, potential cross-language differences in chatbot performance were not assessed. Finally, readability was not evaluated because validated tools specifically suited to Arabic health information remain limited.

Future research should aim to broaden the range of patient-centered questions, including more clinically challenging scenarios, to better represent the diversity of patient concerns related to migraine. It will also be important to evaluate newer and more advanced versions of AI chatbots, as performance may vary across model generations and system configurations. Future studies should include direct comparisons of Arabic and English chatbot responses to determine whether quality, reliability, and accuracy remain consistent across languages. Additionally, further work should focus on developing and validating readability tools suitable for evaluating Arabic health information. Taken together, these efforts may improve the reliability, quality, and clinical usefulness of AI-generated health information.

## 5. Conclusions

Arabic-language chatbot responses to migraine-related questions were broadly similar in medical accuracy but differed significantly in reliability and overall informational quality. These differences appeared to be driven less by factual correctness than by variation in source transparency and the communication of uncertainty. AI chatbots may serve as useful adjuncts in migraine education; however, their outputs should be interpreted with caution and should not replace professional medical consultation. Further studies are needed to improve the reliability and clinical usefulness of Arabic-language AI-generated health information.

## Figures and Tables

**Table 1:** Inter-Rater Comparison and Reliability of Overall Scores.

	Rater 1	Rater 2	Rater 3	Rater 4	ICC	*p*-Value
mDISCERN Score	32.39 (3.66)	31.97 (3.07)	32.46 (3.53)	31.60 (2.37)	0.831	<0.001
GQS Score	4.72 (0.59)	4.74 (0.58)	4.75 (0.58)	4.68 (0.62)	0.816	<0.001
Accuracy Score	4.75 (0.54)	4.74 (0.58)	4.68 (0.57)	4.71 (0.56)	0.799	<0.001

ICC = Intraclass Correlation Coefficient; mDISCERN = Modified DISCERN score; GQS = Global Quality Score; SD = Standard Deviation.

**Table 2:** Comparison of item-level mDISCERN across AI chatbots.

mDISCERN Component Questions	ChatGPT Mean (SD)	DeepSeek Mean (SD)	Gemini Mean (SD)	Grok Mean (SD)	Kruskal–Wallis H	*p*-Value
Q1: Are the aims clear?	4.82 (0.37)	5.00 (0.00)	4.92 (0.35)	4.97 (0.08)	2.318	0.088
Q2: Does it achieve its aims?	4.84 (0.44)	4.96 (0.12)	4.94 (0.11)	4.96 (0.09)	1.753	0.625
Q3: Is it relevant?	4.86 (0.40)	4.96 (0.09)	4.96 (0.09)	4.96 (0.09)	1.923	0.589
Q4: Are the sources of information used to compile the publication clearly identified?	1.63 (0.42)	1.27 (0.76)	1.27 (0.68)	3.77 (1.28)	55.055	<0.001
Q5: Is it clear when the information used or reported in the publication was produced?	4.36 (0.76)	4.78 (0.17)	4.67 (0.68)	4.73 (0.16)	12.357	<0.001
Q6: Is it balanced and unbiased?	4.88 (0.19)	4.75 (0.13)	4.91 (0.19)	4.81 (0.13)	21.471	<0.001
Q7: Does it provide details of additional sources of support and information?	2.35 (0.80)	4.08 (0.68)	2.50 (0.88)	3.50 (0.59)	50.251	<0.001
Q8: Does it refer to areas of uncertainty?	2.09 (1.26)	4.30 (0.54)	2.06 (1.57)	2.56 (1.39)	27.587	<0.001

Values are presented as mean (SD). Statistical comparisons were performed using Kruskal–Wallis Test, with *p* < 0.05 considered statistically significant.

**Table 3:** Comparison of mDISCERN, GQS, and Accuracy Scores Across AI Chatbots.

Metric	ChatGPT Mean (SD), Median (IQR)	DeepSeek Mean (SD), Median (IQR)	Gemini Mean (SD), Median (IQR)	Grok Mean (SD), Median (IQR)	*p*-Value
mDISCERN	29.83 (1.87), 30.25 (28.88–31.50)	34.07 (1.31), 34.00 (33.25–34.75)	30.23 (2.39), 30.00 (28.50–31.38)	34.29 (2.59), 34.25 (32.63–35.75)	<0.001
GQS	4.47 (0.66), 4.75 (4.25–5.00)	4.95 (0.13), 5.00 (5.00–5.00)	4.61 (0.67), 5.00 (4.50–5.00)	4.86 (0.23), 5.00 (4.75–5.00)	<0.001
Accuracy	4.51 (0.61), 5.00 (4.00–5.00)	4.83 (0.32), 5.00 (4.75–5.00)	4.71 (0.62), 5.00 (4.75–5.00)	4.83 (0.31), 5.00 (4.63–5.00)	0.072

Values are presented as mean (SD) and median (interquartile range, IQR). Group comparisons were performed using the Kruskal–Wallis test. A *p*-value < 0.05 was considered statistically significant. Abbreviations: mDISCERN = Modified DISCERN; GQS = Global Quality Score; SD = Standard Deviation; IQR = Interquartile Range.

**Table 4:** Pairwise Comparison of mDISCERN and GQS Among AI chatbots Using Dunn’s Post hoc test.

	mDISCERN			GQS		
	(Z)	(r)	(*p*)	(Z)	(r)	(*p*)
ChatGPT—Gemini	−0.507	−0.051	1.000	−1.607	−0.161	0.649
ChatGPT—Grok	−5.373	−0.537	0.000	−2.589	−0.259	0.058
ChatGPT—DeepSeek	−5.524	−0.552	0.000	−3.893	−0.389	<0.001
Gemini—Grok	−4.865	−0.487	0.000	−0.982	−0.098	1.000
Gemini—DeepSeek	5.017	0.502	0.000	2.286	0.229	0.133
Grok—DeepSeek	0.151	0.015	1.000	1.304	0.130	1.000

Pairwise comparisons were performed using Dunn’s post hoc test following a significant Kruskal–Wallis Test. Z represents the standardized test statistic obtained from the pairwise comparisons. Effect size (r) was calculated using the formula r = Z/√N, where N = 100. *p*-values represent Bonferroni-adjusted significance levels to control for multiple comparisons. Statistical significance was considered at *p* < 0.05. Accuracy pairwise comparisons were not performed because the Kruskal–Wallis test for Accuracy did not show a statistically significant difference across AI bot groups; therefore, post hoc analysis was not applicable.

**Table 5:** Spearman correlation coefficients with 95% confidence intervals among quality scores.

Metric	mDISCERN	GQS	Accuracy
mDISCERN	1	0.499 (0.335–0.633) **	0.412 (0.235–0.563) **
GQS	0.499 (0.335–0.633) **	1	0.769 (0.674–0.839) **
Accuracy	0.412 (0.235–0.563) **	0.769 (0.674–0.839) **	1

Values are presented as Spearman correlation coefficients (r) with 95% confidence intervals. Confidence intervals were calculated using Fisher’s r-to-z transformation. ** *p* < 0.01.

## Data Availability

The data presented in this study are available upon reasonable request from the corresponding author.

## Supplementary Materials

The following supporting information can be downloaded at: https://www.mdpi.com/article/10.3390/jcm15103908/s1, Table S1: Questions and chatbot responses.

## Abbreviations

The following abbreviations are used in this manuscript:

AI	Artificial Intelligence
FAQ	Frequently Asked Question
GQS	Global Quality Scale
ICC	Intraclass Correlation Coefficient
ICHD-3	International Classification of Headache Disorders, 3rd edition
IHS	International Headache Society
IQR	Interquartile Range
IRB	Institutional Review Board
LLM	Large Language Model
mDISCERN	Modified DISCERN
SD	Standard Deviation
SERPs	Search Engine Results Pages
SPSS	Statistical Package for the Social Sciences
