# Supplementary material for: Evaluation of Arabic-Language AI Chatbot Responses to Migraine-Related Questions: A Comparative Cross-Sectional Study
Source: J Clin Med. 2026 May 19;15(10):3908. doi: 10.3390/jcm15103908 (PMC13206779; doi:10.3390/jcm15103908)
Supplement: Supplementary file 1 [file jcm-15-03908-s001.zip › jcm-4286429-supplementary.pdf]

Table S1. Questions and chatbot responses

Chatbot 1 (ChatGPT (4.1)		
الرد	السؤال	SN
**الشقيقة** هي الاسم العربي لمرض يُعرف طبيًا بـ **الصداع النصفي (Migraine)**.<br><br>هي حالة عصبية شائعة تتميز بصداع قوي ومتكرر، وغالبًا يكون في **جانب واحد من الرأس**، وقد يستمر من ساعات إلى أيام.<br><br>**من أعراض الشقيقة:**<br>• ألم نابض أو شديد في الرأس<br>• الغثيان أو القيء<br>• حساسية شديدة للضوء أو الأصوات<br>• تشوش في الرؤية أحيانًا (وميض، خطوط، أو بقع ضوئية)<br>• إرهاق وعدم ارتياح عام<br><br>**أنواع الشقيقة:**<br>• **شقيقة مع أورة**: تسبق الصداع أعراض بصرية أو عصبية.<br>• **شقيقة بدون أورة**: صداع مباشر دون إنذارات.<br><br>**أسبابها المحتملة:**<br>• تغيّرات في كيمياء الدماغ<br>• التوتر والقلق<br>• قلة النوم أو الجوع<br>• بعض الأطعمة (كالشوكولاتة، القهوة، الأجبان)<br>• التغيرات الهرمونية<br><br>**علاجها:**<br>لا يوجد علاج نهائي، لكن يمكن **السيطرة عليها** عبر:<br>• مسكنات خاصة بالصداع النصفي<br>• تجنب المحفزات<br>• تنظيم النوم والغذاء<br>• أدوية وقائية في الحالات الشديدة<br><br>إذا رغبت، أستطيع شرح **العلاج بالتفصيل** أو **الفرق بينها وبين الصداع العادي**.	ما هي الشقيقة؟	1

Chatbot 1 (ChatGPT (4.1)		
الشقيقة (الصداع النصفي) لها عدة أنواع، تختلف حسب الأعراض المصاحبة لها. أهمها:<br><br>1. **الشقيقة بدون أورة**<br>• الأكثر شيوعًا.<br>• صداع نابض غالبًا في جهة واحدة، مع غثيان وحساسية للضوء أو الصوت.<br>2. **الشقيقة مع أورة**<br>• يسبق الصداع أعراض عصبية مؤقتة مثل: ومضات ضوئية، خطوط متعرجة، تنميل، أو صعوبة كلام.<br>• تستمر الأورة عادة من 5 إلى 60 دقيقة.<br>3. **الشقيقة المزمنة**<br>• تحدث 15 يومًا أو أكثر في الشهر لمدة 3 أشهر على الأقل.<br>• قد تكون مع أو بدون أورة.<br>4. **الشقيقة الصامتة (بدون صداع)**<br>• تظهر أعراض الأورة دون حدوث الصداع نفسه.<br>5. **الشقيقة الدهليزية**<br>• تترافق مع دوار شديد، اختلال توازن، أو غثيان، وقد يحدث الصداع أو لا.<br>6. **الشقيقة الشللية (الفلجية)**<br>• نوع نادر.<br>• تسبب ضعفًا مؤقتًا أو شللًا في جانب من الجسم.<br>7. **الشقيقة الشبكية (العينية)**<br>• اضطراب مؤقت في الرؤية في عين واحدة، مثل فقدان جزئي أو ومضات.<br><br>إذا رغبت، أستطيع شرح **أسباب كل نوع، محفزاته، أو طرق العلاج والوقاية.**	ما أنواع الشقيقة؟	2
لا، **لا يوجد فرق** بين **الشقيقة** و**الصداع النصفي** — هما **اسمان لنفس الحالة.**<br><br>🔷**الشقيقة**: التسمية العربية الشائعة<br>🔷 **الصداع النصفي**: ترجمة حرفية للاسم الطبي (Migraine)<br><br>**ما هي الشقيقة؟**<br><br>هي نوع من الصداع العصبي يتميز بـ:<br><br>• ألم نابض غالبًا في **جهة واحدة من الرأس**<br>• قد يكون متوسطًا إلى شديد<br>• يزداد مع الحركة أو المجهود<br>• قد يصاحبه:<br>○ غثيان أو قيء<br>○ حساسية للضوء أو الأصوات<br>○ تشوش بصري أو رؤية وميض (تُسمّى *الأورة*)<br><br>**هل لها أنواع؟**	هل هناك فرق بين الشقيقة والصداع النصفي؟	3

Chatbot 1 (ChatGPT (4.1)		
نعم، من أشهرها:<br><br>• **الشقيقة مع أورة**<br>• **الشقيقة بدون أورة**<br>• **الشقيقة المزمنة**<br><br>إذا رغبت، أستطيع شرح **الأسباب**، **المحفزات الشائعة** (مثل التوتر أو قلة النوم)، أو **طرق العلاج والوقاية**.		
الفرق بين **الشقيقة (الصداع النصفي)** وصداع **الجيوب الأنفية** يكون في **السبب، الأعراض، ومكان الألم**.<br>إليك توضيحًا مبسّطًا:<br><br>---<br><br>## أولًا: الشقيقة (الصداع النصفي)<br><br>**السبب:** اضطراب عصبي يؤثر في طريقة عمل الدماغ والأعصاب والأوعية الدموية.<br><br>**الأعراض الشائعة:**<br><br>• ألم نابض أو خافق<br>• غالبًا في جهة واحدة من الرأس<br>• متوسط إلى شديد<br>• يزداد مع الحركة<br>• غثيان أو قيء<br>• حساسية شديدة للضوء أو الصوت<br>• أحيانًا تسبقها أعراض تُسمّى *الأورة* (زغللة، وميض، تنميل)<br><br>**المدة:** من عدة ساعات إلى 3 أيام<br><br>---<br><br>## ثانيًا: صداع الجيوب الأنفية<br><br>**السبب:** التهاب أو احتقان في الجيوب الأنفية (غالبًا بسبب زكام أو حساسية أو التهاب بكتيري).<br><br>**الأعراض الشائعة:**<br><br>• ألم ضاغط أو ثقيل (ليس نابضًا)<br>• في الجبهة، حول العينين، الأنف، أو الخدين<br>• يزداد عند الانحناء للأمام<br>• انسداد أو سيلان الأنف<br>• إفرازات أنفية سميكة	ما الفرق بين الشقيقة وصداع الجيوب الأنفية؟	4

Chatbot 1 (ChatGPT (4.1)

- أحيانًا حمى وضعف في حاسة الشم

المدة: يستمر طالما الالتهاب موجود

مقارنة سريعة

وجه المقارنة	الشقيقة	صداع الجيوب الأنفية
نوع الألم	نابض	ضاغط
المكان	غالبًا جهة واحدة	الوجه والجبهة
غثيان/قيء	شائع	نادر
انسداد الأنف	لا	نعم
الحساسية للضوء	نعم	لا
يزداد بالانحناء	لا	نعم

ملاحظة مهمة

كثير من الناس يعتقدون أن لديهم صداع جيوب أنفية، بينما هو في الحقيقة **شقيقة**، لأن الشقيقة قد تُسبب ألمًا في الوجه واحتقانًا خفيفًا.

إذا تحب، أقدر أساعدك **تحديدًا** من خلال وصف الأعراض التي تشعر بها، أو أشرح **متى يجب مراجعة الطبيب**.

5	هل صداع الشقيقة خطير؟	صداع الشقيقة (الصداع النصفي) **غالبًا ليس خطيرًا** ولا يسبب تلفًا دائمًا في الدماغ، لكنه قد يكون **مؤلمًا ومزعجًا جدًا** ويؤثر على جودة الحياة.<br><br>**متى لا يكون خطيرًا؟**<br><br>• إذا كان الصداع متكررًا بنفس النمط المعتاد لديك

<table>
<tr><th colspan="3">Chatbot 1 (ChatGPT (4.1)</th></tr>
<tr><td></td><td></td><td>

- يستمر من ساعات إلى يومين
- يصاحبه غثيان، قيء، حساسية للضوء أو الصوت
- يتحسن بالراحة أو الأدوية المخصصة للشقيقة

متى قد يحتاج انتباهًا طبيًا؟ (علامات إنذار 🚨)
راجع الطبيب فورًا إذا كان الصداع:

- **مفاجئًا وشديدًا جدًا** (أسوأ صداع في حياتك)
- مختلفًا تمامًا عن صداعك المعتاد
- مصحوبًا بـ **ضعف في أحد الأطراف، صعوبة كلام، تشوش، فقدان وعي**
- مع **حمّى، تيبس الرقبة، أو تشنجات**
- بدأ بعد **إصابة في الرأس**
- يزداد سوءًا باستمرار أو لا يستجيب للعلاج
- ظهر لأول مرة بعد سن 50

هل للشقيقة مضاعفات؟

- نادرًا جدًا قد ترتبط ببعض المضاعفات، لكن هذا غير شائع
- التأثير الأكبر يكون على الحياة اليومية (العمل، الدراسة، النوم)

الخلاصة:
الشقيقة **ليست خطيرة في معظم الحالات**، لكنها تستحق التشخيص والعلاج المناسبين لتقليل الألم وتكرار النوبات.

إذا أحببت، أستطيع:

- شرح **أسباب الشقيقة**
- اقتراح **طرق الوقاية**
- أو توضيح **أفضل العلاجات المتاحة** حسب حالتك 🌿

</td></tr>
<tr><td>6</td><td>ما هي أعراض الشقيقة؟ وما هي المراحل التي قد تمر بها النوبة؟</td><td>

الشقيقة (الصداع النصفي) هي اضطراب عصبي يتميز بصداع متكرر وقد ترافقه أعراض أخرى. تختلف شدته وأعراضه من شخص لآخر، لكن غالبًا تمر النوبة بأعراض ومراحل معروفة:

أولًا: أعراض الشقيقة

قد تشمل واحدة أو أكثر من التالي:

🧠 أعراض الصداع

- ألم نابض أو خافق، غالبًا في جهة واحدة من الرأس
- يزداد مع الحركة أو المجهود

</td></tr>
</table>

Chatbot 1 (ChatGPT (4.1)

- يستمر من **4 ساعات إلى 72 ساعة**

أعراض مرافقة

- غثيان أو قيء
- حساسية شديدة للضوء (رهاب الضوء)
- حساسية للأصوات أو الروائح
- دوار أو تعب عام
- صعوبة في التركيز

ثانيًا: مراحل نوبة الشقيقة

ليست كل النوبات تمر بجميع المراحل، وقد تختلف من شخص لآخر:

1️⃣ المرحلة التمهيدية (البادرة) – *قبل الصداع بساعات أو أيام*

- تقلبات مزاجية
- تعب أو نعاس
- اشتهاء أطعمة معينة
- تيبس في الرقبة
- كثرة التثاؤب

2️⃣ مرحلة الأورة (الهالة) – *تحدث لدى بعض المرضى فقط*

تسبق الصداع أو ترافقه، وتستمر عادة **20–60 دقيقة**:

- اضطرابات بصرية (ومضات ضوء، خطوط متعرجة، بقع عمياء)
- تنميل أو وخز في الوجه أو اليد
- صعوبة في الكلام أو التفكير

3️⃣ مرحلة الصداع

- ألم متوسط إلى شديد
- يزداد مع النشاط
- غثيان، قيء، وانزعاج من الضوء والصوت

4️⃣ مرحلة ما بعد الصداع (الإنهاك)

- تعب شديد
- ثقل في الرأس
- ضعف التركيز

Chatbot 1 (ChatGPT (4.1)		
		• تحسن تدريجي خلال ساعات أو يوم<br><br>**متى يجب مراجعة الطبيب؟**<br>• إذا كان الصداع **شديدًا أو مختلفًا عن المعتاد**<br>• إذا ترافق مع ضعف مفاجئ، تشوش شديد، أو فقدان وعي<br>• إذا بدأ بعد سن 50<br>• إذا أصبح متكررًا أو يؤثر على الحياة اليومية<br><br>إذا رغبت، أستطيع شرح **الأسباب الشائعة**، **المحفزات**، **أو طرق العلاج والوقاية**🌿
7	ما هي الهالة (الأورة) في الشقيقة؟	**الهالة (الأورة) في الشقيقة** هي مجموعة أعراض عصبية **مؤقتة** تظهر عند بعض مرضى الشقيقة (الصداع النصفي)، وغالبًا **تسبق الصداع** نفسه، وقد ترافقه أحيانًا أو نادرًا تأتي بدونه.<br><br>**ما الذي يشعر به المريض أثناء الهالة؟**<br>أشيعها **الأعراض البصرية**، مثل:<br>• ومضات أو خطوط متعرجة لامعة<br>• بقع عمياء أو تشوش في جزء من مجال الرؤية<br>• رؤية أشكال هندسية أو ألوان غير معتادة<br><br>وقد تظهر أعراض أخرى، مثل:<br>• **تنميل أو وخز** في الوجه أو اليد أو الذراع<br>• **صعوبة مؤقتة في الكلام** أو إيجاد الكلمات<br>• **دوار أو ضعف مؤقت** (أقل شيوعًا)<br><br>**كم تدوم الهالة؟**<br>• عادةً من **5 إلى 60 دقيقة**<br>• تختفي تدريجيًا ثم يبدأ الصداع النصفي (إن حدث)<br><br>**لماذا تحدث؟**<br>يُعتقد أنها ناتجة عن **نشاط كهربائي غير طبيعي مؤقت** ينتشر في قشرة الدماغ، يليه تغير عابر في تدفق الدم إلى مناطق معينة.<br><br>**هل الهالة خطيرة؟**

Chatbot 1 (ChatGPT (4.1)		
- غالبًا **غير خطيرة بحد ذاتها** - لكن إذا كانت الأعراض **جديدة، شديدة، أو مختلفة عن المعتاد** (خصوصًا ضعف مفاجئ أو اضطراب كلام واضح)، فيجب استبعاد أسباب أخرى مثل السكتة الدماغية بمراجعة الطبيب فورًا. **ملاحظة مهمة**<br><br>ليس كل من يعاني من الشقيقة لديه هالة؛ فقط حوالي **20–30٪** من المرضى.<br><br>إذا رغبت، أستطيع شرح **الفرق بين هالة الشقيقة والسكتة الدماغية** أو **كيف تُعالج الشقيقة المصحوبة بهالة**.		
نعم، **الغثيان والقيء من الأعراض الشائعة جدًا للشقيقة (الصداع النصفي)**.<br>كثير من المصابين بالشقيقة يعانون منها، وأحيانًا تكون مزعجة بقدر الصداع نفسه.<br><br>**أعراض مرافقة أخرى للشقيقة قد تشمل:** - حساسية شديدة للضوء أو الأصوات - ألم نابض غالبًا في جهة واحدة من الرأس - دوخة أو تعب شديد - ازدياد الألم مع الحركة أو المجهود - أحيانًا تشوش بصري أو وميض (الهالة) **ملاحظة مهمة:**<br>إذا كان القيء شديدًا أو متكررًا بدون صداع، أو مصحوبًا بحمى، تيبس في الرقبة، أو ضعف مفاجئ في أحد الأطراف، فهنا يجب مراجعة الطبيب فورًا لأن السبب قد لا يكون شقيقة.<br><br>إذا رغبت، أستطيع: - شرح كيفية التفريق بين الشقيقة وأسباب أخرى للغثيان - أو اقتراح طرق للتخفيف من الغثيان أثناء نوبة الشقيقة	هل القيء والغثيان من أعراض الشقيقة؟	8
نعم، **قد يكون لطنين الأذن وآلام الرقبة علاقة بالشقيقة (الصداع النصفي)**، لكن ليست العلاقة واحدة عند الجميع. إليك التوضيح:<br><br>**🔷 طنين الأذن والشقيقة** - بعض مرضى الشقيقة يعانون من **طنين الأذن أو امتلاء أو ضغط بالأذن** أثناء النوبة أو قبلها. - السبب يُعتقد أنه مرتبط بـ **تغيرات عصبية وتدفق الدم** في مناطق قريبة من مراكز السمع. - يوجد نوع خاص يُسمّى **الشقيقة الدهليزية** قد يسبب:	هل طنين الأذن أو آلام الرقبة لها علاقة بالشقيقة؟	9

Chatbot 1 (ChatGPT (4.1)		
○ طنين الأذن<br>○ دوار<br>○ عدم توازن<br>حتى بدون صداع شديد أحيانًا.<br><br>◈ **آلام الرقبة والشقيقة**<br><br>• **ألم الرقبة شائع جدًا مع الشقيقة:**<br>○ قد يكون **عرضًا مرافقًا** للشقيقة<br>○ أو **إنذارًا مبكرًا** قبل بدء الصداع<br>• التوتر في عضلات الرقبة والكتفين قد:<br>○ يحرّض نوبة الشقيقة<br>○ أو يزيد شدتها<br>• أحيانًا يختلط الأمر بينها وبين **صداع التوتر** أو الصداع العنقي.<br><br>◈ **متى ننتبه؟**<br><br>راجع الطبيب إذا كان:<br><br>• الطنين **مستمرًا أو في أذن واحدة فقط**<br>• هناك **ضعف سمع، دوار شديد، خدر، أو ضعف بالأطراف**<br>• ألم الرقبة شديد أو بعد إصابة<br>• الصداع جديد أو مختلف عن المعتاد<br><br>◈ **الخلاصة**<br><br>✔ نعم، يمكن أن يكون طنين الأذن وآلام الرقبة جزءًا من الشقيقة<br>✔ لكن وجودهما لا يعني دائمًا أن السبب هو الشقيقة فقط<br>✔ التقييم الطبي مهم إذا كانت الأعراض غير معتادة أو مستمرة<br><br>إذا رغبت، أستطيع:<br><br>• مساعدتك في التمييز بين **الشقيقة وصداع التوتر**<br>• أو اقتراح **طرق تخفيف آلام الرقبة المرتبطة بالشقيقة**<br>• أو شرح **الشقيقة الدهليزية** بتفصيل أكثر		
الشقيقة (الصداع النصفي) هي اضطراب عصبي شائع، وسببها **ليس واحدًا محددًا** بل تداخل عوامل وراثية وعصبية وبيئية. إليك توضيحًا منظمًا:	ما أسباب الشقيقة؟ وما هي محفزات النوبات؟	10

Chatbot 1 (ChatGPT (4.1)

أولًا: أسباب الشقيقة

لا يوجد سبب واحد قاطع، لكن أهم الآليات المعروفة تشمل:

1. **عوامل وراثية**
   - الشقيقة أكثر شيوعًا عند من لديهم تاريخ عائلي معها.
2. **اضطراب في كيمياء الدماغ**
   - خاصة تغيّرات في مادة **السيروتونين** التي تؤثر على إشارات الألم والأوعية الدموية.
3. **تغيرات في الأوعية الدموية**
   - توسّع وتضيّق غير طبيعي في أوعية الدماغ يساهم في الألم.
4. **فرط حساسية الجهاز العصبي**
   - دماغ مريض الشقيقة يكون أكثر حساسية للمؤثرات (الضوء، الصوت، الروائح).

ثانيًا: محفزات نوبات الشقيقة

المحفزات تختلف من شخص لآخر، ومن أشهرها:

1. محفزات غذائية

- الشوكولاتة
- الجبن المعتّق
- الكافيين (الإفراط أو الانقطاع المفاجئ)
- الأطعمة المصنعة (التي تحتوي على MSG أو نترات)
- الصيام أو تخطي الوجبات

2. عوامل نفسية وعصبية

- التوتر والقلق
- الضغط النفسي المفاجئ أو زوال التوتر بعد فترة ضغط
- الاكتئاب

3. اضطرابات النوم

- قلة النوم
- النوم الزائد
- تغيّر مواعيد النوم

4. عوامل هرمونية (شائعة عند النساء)

- الدورة الشهرية
- الحمل

Chatbot 1 (ChatGPT (4.1)		
		• استخدام أو إيقاف موانع الحمل الهرمونية<br><br>**5. عوامل حسية وبيئية**<br><br>• الضوء الساطع أو الوميض<br>• الأصوات العالية<br>• الروائح القوية (عطور، دخان)<br>• تغيرات الطقس أو الضغط الجوي<br><br>**6. عوامل جسدية**<br><br>• الإجهاد البدني<br>• الجفاف<br>• الجلوس الخاطئ لفترات طويلة<br><br>---<br><br>**نقطة مهمة 🌿**<br><br>ليس كل محفز يسبب نوبة عند الجميع.<br>يفيد كثيرًا **تدوين يوميات الصداع** لمعرفة محفزاتك الشخصية وتجنبها.<br><br>إذا رغبت، أستطيع:<br><br>• شرح الفرق بين الشقيقة والصداع العادي<br>• أو ذكر أعراض الإنذار المبكر للشقيقة<br>• أو طرق الوقاية والعلاج (دوائي وغير دوائي)<br><br>اختر ما يناسبك.
11	هل الشقيقة مرض وراثي ومن هم الأكثر عرضة للإصابة؟	نعم، **الشقيقة (الصداع النصفي)** غالبًا لها **عامل وراثي**، لكنها ليست مرضًا وراثيًا بسيطًا بمعنى جين واحد فقط، بل **تنتج عن تداخل الوراثة مع عوامل بيئية ونمط الحياة**.<br><br>**هل الشقيقة وراثية؟**<br><br>• حوالي **60–80٪** من المصابين بالشقيقة لديهم **تاريخ عائلي** للمرض.<br>• وجود أحد الوالدين مصابًا يزيد احتمال الإصابة، ويكون الاحتمال أعلى إذا كان **كلا الوالدين** مصابين.<br>• توجد أنواع نادرة جدًا (مثل الشقيقة الفالجية العائلية) تكون **وراثية بشكل أوضح**.<br><br>**من هم الأكثر عرضة للإصابة بالشقيقة؟**

Chatbot 1 (ChatGPT (4.1)		
1. **النساء**<br>○ أكثر عرضة من الرجال (حوالي 3 أضعاف)، ويُرجّح أن يكون ذلك بسبب **التغيرات الهرمونية**.<br>2. **من لديهم تاريخ عائلي**<br>○ إصابة أحد أفراد العائلة تزيد الخطر.<br>3. **الأعمار بين 15–55 سنة**<br>○ غالبًا تبدأ في المراهقة أو بداية البلوغ.<br>4. **من يتأثرون بعوامل محفِّزة** مثل:<br>○ التوتر والقلق<br>○ قلة النوم أو اضطرابه<br>○ بعض الأطعمة (الشوكولاتة، الجبن المعتق، الكافيين الزائد)<br>○ التغيرات الهرمونية (الدورة الشهرية)<br>○ الإضاءة القوية أو الأصوات العالية<br>5. **مرضى بعض الحالات الصحية**<br>○ مثل القلق والاكتئاب، أو القولون العصبي.<br><br>**خلاصة**<br><br>● الشقيقة **ليست حتمية وراثيًا**، لكن الوراثة تزيد الاستعداد.<br>● يمكن تقليل النوبات عبر **تجنب المحفزات، تنظيم النوم، وإدارة التوتر**، ومع العلاج المناسب عند الحاجة.<br><br>إذا رغبت، أستطيع توضيح **الفرق بين الشقيقة والصداع العادي** أو **متى يجب مراجعة الطبيب**.		
قد تُحفِّز بعض الأطعمة والمشروبات نوبات **الشقيقة (الصداع النصفي)** لدى بعض الأشخاص، لكن المحفزات تختلف من شخص لآخر. من أكثرها شيوعًا:<br><br>**أطعمة ومشروبات محتملة التحفيز**<br><br>● **الأجبان المعتّقة** (مثل الشيدر، البارميزان، الروكفور) لاحتوائها على *التيرامين*.<br>● **اللحوم المُعالجة** (السلامي، السجق، النقانق) بسبب *النتريت/النترات*.<br>● **الشوكولاتة** (تحتوي كافيين ومواد قد تؤثر على الأوعية الدموية).<br>● **الكافيين**: الإفراط فيه أو الانسحاب المفاجئ عنه (قهوة، شاي، مشروبات طاقة).<br>● **المشروبات الكحولية**، خاصة **النبيذ الأحمر**.<br>● **محليات صناعية** مثل الأسبارتام.<br>● **الأطعمة الغنية بغلوتامات أحادي الصوديوم ((MSG** (بعض الأطعمة الآسيوية الجاهزة، الشيبس).<br>● **أطعمة مخمّرة أو مخللة** (مخللات، صلصة الصويا).<br>● **المكسرات** لدى بعض الأشخاص.<br>● **الحمضيات** (برتقال، ليمون) عند فئة معينة.<br>● **الخبز الطازج جدًا أو المخمّر** (خميرة نشطة).	ما الأطعمة التي قد تحفز نوبات الشقيقة؟	12

<table dir="rtl">
<tr><th colspan="3">Chatbot 1 (ChatGPT (4.1)</th></tr>
<tr><td></td><td></td><td>

نصائح عملية

- احتفظ/ي **بمفكرة للشقيقة** لتسجيل ما تأكله قبل النوبة بـ 24 ساعة.
- جرّب/ي **الاستبعاد التدريجي**: أزل طعامًا واحدًا مشتبهًا به لعدة أسابيع ثم راقِب.
- انتبه/ي أيضًا لعوامل غير غذائية: **قلة النوم، الجفاف، التوتر، تخطي الوجبات**—قد تتفاعل مع الطعام.
- الحفاظ على **مواعيد وجبات منتظمة** وشرب ماء كافٍ يساعد كثيرًا.

إذا رغبت، أستطيع مساعدتك في إعداد **قائمة طعام مناسبة** أو نموذج **مفكرة تتبع** لتحديد محفزاتك بدقة.

</td></tr>
<tr><td>13</td><td>كيف يتم تشخيص الشقيقة؟</td><td>

يتم تشخيص **الشقيقة (الصداع النصفي)** أساسًا **سريريًا**، أي بالاعتماد على وصف الأعراض والتاريخ المرضي، ولا يوجد تحليل أو صورة واحدة تؤكدها بشكل قاطع. ويتم ذلك عبر الخطوات التالية:

1 أخذ التاريخ المرضي بالتفصيل

يسأل الطبيب عن:

- طبيعة الصداع (نابض/ضاغط)
- مكانه (غالبًا في جهة واحدة من الرأس)
- شدته ومدته (من 4 إلى 72 ساعة)
- عدد النوبات وتكرارها
- الأعراض المصاحبة مثل:
  - الغثيان أو القيء
  - الحساسية للضوء أو الصوت
  - تشوش الرؤية أو ظهور أضواء (الأورة)
- العوامل المحفِّزة (قلة النوم، التوتر، بعض الأطعمة، الدورة الشهرية)
- وجود تاريخ عائلي للشقيقة

2 تطبيق معايير تشخيصية (معايير الجمعية الدولية للصداع)

غالبًا يُشخَّص الصداع النصفي إذا:

- حدثت ≥ 5 نوبات صداع
- استمر الصداع 4–72 ساعة
- تميز باثنين على الأقل من:
  - جهة واحدة
  - طبيعة نابضة
  - شدة متوسطة إلى شديدة
  - يزداد مع النشاط البدني
- ترافق مع غثيان/قيء أو حساسية للضوء والصوت

3 الفحص السريري والعصبي

</td></tr>
</table>

<table>
<tr><th colspan="3">Chatbot 1 (ChatGPT (4.1)</th></tr>
<tr><td></td><td></td><td>

يكون طبيعيًا في الشقيقة
الهدف هو استبعاد أسباب أخرى خطيرة

<h3>4️⃣ الفحوصات والصور (عند الحاجة فقط)</h3>
لا تُطلب عادةً إلا إذا وُجدت علامات إنذار مثل:

صداع جديد بعد سن 50
صداع مفاجئ شديد جدًا
تغير في نمط الصداع
ضعف أو خدر دائم
حمى أو تشنجات

وقد تشمل:

تصوير بالرنين المغناطيسي (MRI)
أو الأشعة المقطعية (CT)

<h3>5️⃣ يوميات الصداع (مهمة جدًا)</h3>
قد يُطلب من المريض تسجيل:

وقت الصداع
شدته
مدته
المحفزات
الأدوية المستخدمة

📌 ملاحظة مهمة:
الشقيقة لا تظهر في تحاليل الدم أو الأشعة، لذلك يعتمد التشخيص على الوصف الدقيق للأعراض.
إذا رغبت، أستطيع:

مساعدتك في التفريق بين الشقيقة والصداع التوتري أو العنقودي
أو شرح أنواع الشقيقة
أو خطوات العلاج والوقاية

</td></tr>
<tr><td>14</td><td>ما مدى شيوع الشقيقة؟</td><td>
الشقيقة (الصداع النصفي) شائعة نسبيًا على مستوى العالم. تشير التقديرات إلى أنها تصيب نحو 10–15٪ من السكان.
بعض النقاط المهمة:

أكثر شيوعًا لدى النساء من الرجال (حوالي 3 أضعاف)، ويرتبط ذلك بالتغيرات الهرمونية.

</td></tr>
</table>

Chatbot 1 (ChatGPT (4.1)		
		• غالبًا ما تبدأ في **سن المراهقة أو الشباب**، وتبلغ ذروتها في منتصف العمر.<br>• تُعد من **أسباب الإعاقة الشائعة** بسبب شدة الألم وتأثيرها على الأداء اليومي.<br>• قد تصيب الأطفال أيضًا، وإن كانت بنسبة أقل.<br><br>إذا رغبت، يمكنني توضيح **أسباب الشيوع**، أو **العوامل المحفِّزة**، أو **طرق الوقاية والعلاج**.
15	في أي عمر تبدأ نوبات الشقيقة؟	تبدأ **نوبات الشقيقة (الصداع النصفي)** في أعمار مختلفة، لكن الأكثر شيوعًا هو:<br><br>• **الطفولة المتأخرة أو المراهقة**: يمكن أن تبدأ حتى من عمر **5–10 سنوات**، وأحيانًا أبكر.<br>• **المراهقة وبداية العشرينات**: هذا هو العمر **الأكثر شيوعًا لظهور الشقيقة لأول مرة**.<br>• **مرحلة البلوغ المبكر (20–40 سنة)**: قد تظهر لأول مرة أيضًا، خاصة عند النساء.<br><br>ملاحظات مهمة:<br><br>• عند **الأطفال** قد تختلف الأعراض عن البالغين (ألم أقل وضوحًا، غثيان أو قيء أكثر).<br>• الشقيقة **أكثر شيوعًا عند الإناث** بعد البلوغ، ويرتبط ذلك بالتغيرات الهرمونية.<br>• وجود **تاريخ عائلي** يزيد احتمال ظهورها في سن مبكرة.<br><br>إذا رغبت، أستطيع توضيح **أعراض الشقيقة حسب العمر** أو **الفرق بينها وبين الصداع العادي**.
16	هل يمكن أن يصاب الأطفال بالشقيقة؟	نعم، **يمكن أن يُصاب الأطفال بالشقيقة (الصداع النصفي)**، وهي ليست مقتصرة على البالغين فقط.<br><br>**معلومات مهمة عن الشقيقة عند الأطفال:**<br><br>• قد تبدأ في أي عمر، حتى قبل سن المدرسة.<br>• تكون نوبات الصداع عند الأطفال غالبًا **أقصر مدة** من البالغين.<br>• قد يكون الألم **في جانبي الرأس** وليس جانبًا واحدًا فقط.<br>• ترافقها أحيانًا أعراض مثل:<br>○ الغثيان أو القيء<br>○ حساسية للضوء أو الأصوات<br>○ آلام في البطن أو دوار<br>• بعض الأطفال لا يشتكون من الصداع بوضوح، بل من **ألم بطني متكرر** (ما يُسمى الشقيقة البطنية).<br><br>**متى يُنصح بمراجعة الطبيب؟**<br><br>• إذا كان الصداع شديدًا أو متكررًا<br>• إذا كان يوقظ الطفل من النوم<br>• إذا ترافق مع قيء متكرر، تشنجات، أو تغير في السلوك أو الرؤية<br>• إذا أثّر على الدراسة أو النشاط اليومي<br><br>**العلاج** يشمل:

Chatbot 1 (ChatGPT (4.1)

		• تجنب المحفزات (قلة النوم، الجوع، التوتر)<br>• تنظيم النوم والوجبات<br>• أدوية مسكنة مناسبة لعمر الطفل، وقد يصف الطبيب أدوية خاصة عند اللزوم<br><br>إذا رغبت، أستطيع مساعدتك في **تمييز الشقيقة عن أنواع الصداع الأخرى عند الأطفال** أو شرح **طرق الوقاية** بشكل عملي.
17	هل الشقيقة مرض مزمن؟	نعم، **الشقيقة (الصداع النصفي)** تُعد **مرضًا مزمنًا** في كثير من الحالات، لكن ليس دائمًا بالشكل نفسه عند كل الناس.<br><br>**التوضيح:**<br><br>• الشقيقة هي اضطراب عصبي يميل إلى **التكرار على مدى سنوات**.<br>• عند بعض الأشخاص تكون النوبات **متباعدة وخفيفة** ويمكن السيطرة عليها.<br>• عند آخرين قد تكون **متكررة وشديدة** وتؤثر على الحياة اليومية.<br><br>**متى تُسمّى شقيقة مزمنة؟**<br><br>• إذا حدث الصداع **15 يومًا أو أكثر في الشهر**<br>• ولمدة **3 أشهر متتالية**<br>• وكان منها **8 أيام على الأقل بصفات الشقيقة**<br><br>في هذه الحالة تُسمّى **الشقيقة المزمنة**.<br><br>**هل يمكن الشفاء منها؟**<br><br>• لا يوجد "شفاء نهائي" حتى الآن<br>• لكن يمكن **التحكم بها بشكل ممتاز** عبر:<br>○ أدوية وقائية<br>○ أدوية لعلاج النوبة<br>○ تجنب المحفزات (قلة النوم، التوتر، بعض الأطعمة)<br>○ تنظيم نمط الحياة<br><br>إذا أحببت، أستطيع:<br><br>• شرح **أعراض الشقيقة** بالتفصيل<br>• أو **الفرق بينها وبين الصداع العادي**<br>• أو **طرق العلاج والوقاية** 🌿
18	ما مضاعفات الشقيقة؟	الشقيقة (الصداع النصفي) غالبًا مرض **غير خطير**، لكن قد تترتب عليها **مضاعفات** عند بعض الأشخاص، خاصة إذا كانت شديدة أو متكررة أو غير مُعالجة جيدًا. من أهمها:

Chatbot 1 (ChatGPT (4.1)

🧠 مضاعفات عصبية

- **حالة الشقيقة المستمرة (Status migrainosus):**
نوبة شديدة تستمر أكثر من 72 ساعة وقد تحتاج علاجًا في المستشفى.
- **الشقيقة المزمنة:**
عندما يحدث الصداع ≥ 15 يومًا في الشهر لمدة 3 أشهر أو أكثر.
- **الصداع الناتج عن الإفراط في المسكنات:**
بسبب الاستخدام المتكرر للأدوية المسكنة.

⚠️ مضاعفات مرتبطة بالهالة (Aura)

- **زيادة طفيفة في خطر السكتة الدماغية**
خاصة عند المصابين بشقيقة مع هالة، وبشكل أوضح لدى:
  - النساء
  - المدخنين
  - مستخدمي حبوب منع الحمل الهرمونية
- **اضطرابات بصرية نادرة**
مثل الشقيقة الشبكية، وقد يحدث ضعف مؤقت في الرؤية (نادراً دائم).

♡ مضاعفات عامة مرتبطة بالصحة

- ارتباط أعلى نسبيًا بـ:
  - أمراض القلب والأوعية الدموية (علاقة وليست سببًا مباشرًا)
  - القلق والاكتئاب
  - اضطرابات النوم

😖 تأثيرات على الحياة اليومية

- ضعف الأداء الدراسي أو الوظيفي
- التغيب المتكرر عن العمل أو الدراسة
- تدهور جودة الحياة والعلاقات الاجتماعية

متى تستدعي القلق؟

راجع الطبيب فورًا إذا كان الصداع:

- مختلفًا أو أشد من المعتاد
- مصحوبًا بضعف مفاجئ، اضطراب كلام، أو فقدان وعي
- ظهر لأول مرة بعد سن 50
- ترافق مع حمى أو تيبس في الرقبة

إذا رغبت، أستطيع شرح **طرق الوقاية** أو **العلاج** أو **الفرق بين الشقيقة وأنواع الصداع الأخرى**.

Chatbot 1 (ChatGPT (4.1)		
نعم، **قد تتحسّن الشقيقة (الصداع النصفي) مع التقدّم في العمر عند كثير من الناس، لكن ليس عند الجميع**. الأمر يختلف من شخص لآخر، وهذه أهم النقاط:<br><br>**كيف تتغيّر الشقيقة مع العمر؟**<br><br>• **كثير من المرضى يلاحظون أن النوبات تصبح أقل تكرارًا** أو **أقل شدة** مع مرور السنوات.<br>• عند **النساء**، غالبًا ما تتحسّن الشقيقة بعد **انقطاع الطمث** بسبب استقرار الهرمونات.<br>• عند **الرجال**، قد تقل الشقيقة تدريجيًا مع العمر أيضًا، لكن التحسّن يكون أبطأ أحيانًا.<br><br>**متى قد لا تتحسّن؟**<br><br>• بعض الأشخاص تستمر لديهم الشقيقة لكن **بأعراض مختلفة** (ألم أقل، دون غثيان مثلًا).<br>• في حالات أخرى قد تختفي نوبات الألم لكن **تستمر الأعراض العصبية** (مثل الشقيقة الصامتة).<br><br>**عوامل تؤثر على التحسّن**<br><br>• السيطرة على **التوتر**<br>• انتظام **النوم**<br>• تجنّب **المحفزات** (مثل بعض الأطعمة أو قلة النوم)<br>• العلاج الوقائي المناسب<br><br>**نقطة مهمة**<br><br>إذا ظهر صداع جديد أو تغيّر نمط الصداع **بعد سن الخمسين**، فيجب مراجعة الطبيب للتأكد من عدم وجود سبب آخر.<br><br>إذا أحببت، أستطيع:<br><br>• توضيح الفرق بين الشقيقة وأنواع الصداع الأخرى<br>• أو إعطاء نصائح عملية لتقليل النوبات حسب نمط حياتك	هل تتحسن الشقيقة مع التقدم بالعمر؟	19
نوبات **الشقيقة (الصداع النصفي)** لها نوعان من العلاج:<br>**علاج لإيقاف النوبة عند حدوثها**، و**علاج وقائي لتقليل تكرارها.**<br><br>---<br><br>**أولًا: علاج النوبة عند حدوثها (العلاج الحاد)5**<br><br>يكون الهدف إيقاف الألم والأعراض المصاحبة (الغثيان، الحساسية للضوء والصوت).<br><br>**1) مسكنات الألم**	ما علاج نوبات الشقيقة؟ و متى يجب أخذ العلاج؟	20

Chatbot 1 (ChatGPT (4.1)		
تفيد في النوبات الخفيفة إلى المتوسطة:<br>• باراسيتامول<br>• إيبوبروفين أو نابروكسين<br>• أسبرين<br>(ويُفضّل أخذها مع بداية النوبة)<br><br>**2) أدوية التريبتان (Triptans)**<br><br>تُستخدم للنوبات المتوسطة إلى الشديدة، مثل:<br>• سوماتريبتان<br>• ريزاتريبتان<br>• زولميتريبتان<br><br>تعمل على أوعية الدم في الدماغ وهي فعّالة جدًا عند استخدامها مبكرًا.<br><br>**3) أدوية للغثيان (عند الحاجة)**<br><br>مثل:<br>• ميتوكلوبراميد<br>• دومبيريدون		
**متى يجب أخذ علاج النوبة؟**<br><br>**في أقرب وقت ممكن عند بداية الصداع أو عند أول إحساس بالشقيقة**<br>⚠️ لا يُنصح بالانتظار حتى يشتد الألم، لأن فعالية الدواء تقل مع التأخير.		
**ثانيًا: العلاج الوقائي (الوقاية من النوبات)**<br><br>يُؤخذ يوميًا، وليس عند حدوث الصداع.<br><br>**يُنصح به إذا:**<br>• كانت النوبات ≥ 4 مرات شهريًا<br>• أو كانت النوبات شديدة وتُعطّل الحياة اليومية<br>• أو لم تنجح أدوية النوبات الحادة		

<table>
<tr><th colspan="3">Chatbot 1 (ChatGPT (4.1)</th></tr>
<tr><td></td><td></td><td>
أمثلة على الأدوية الوقائية:

بعض أدوية الضغط: مثل بروبرانولول
أدوية الصرع: مثل توبيراميت
بعض مضادات الاكتئاب
حقن أو أدوية حديثة موجهة للشقيقة (CGRP)

(يُحددها الطبيب حسب الحالة)
<hr>
<h2>نصائح مهمة</h2>

تجنّب الإكثار من المسكنات (أكثر من 10 أيام شهريًا) لتفادي صداع الإفراط الدوائي
حاول تحديد المحفزات (قلة النوم، الجوع، التوتر، بعض الأطعمة)
النوم المنتظم، شرب الماء، وتقليل الكافيين يساعد كثيرًا

<hr>
إذا أحببت، أخبرني:

كم مرة تأتيك النوبات في الشهر؟
هل يصاحبها غثيان أو تشوش بصري؟

بناءً على ذلك أشرح لك الخيار العلاجي الأنسب 🌿
</td></tr>
<tr><td>21</td><td>ما الأدوية الوقائية لنوبات الشقيقة؟</td><td>
الأدوية الوقائية لنوبات الشقيقة (الصداع النصفي) تُستخدم لتقليل عدد النوبات وشدتها ومدتها، ولا تُؤخذ عند حدوث النوبة بل بشكل يومي أو دوري. تُنصح عادةً إذا كانت النوبات متكررة أو شديدة.

أهم المجموعات الدوائية:
<h3>1️⃣ أدوية القلب وضغط الدم</h3>

حاصرات بيتا:
مثل بروبرانولول، ميتوبرولول
حاصرات قنوات الكالسيوم:
مثل فيراباميل
✔ فعّالة عند كثير من المرضى
⚠ تُتجنب في بعض أمراض الربو أو بطء القلب

<h3>2️⃣ أدوية الصرع</h3>
</td></tr>
</table>

Chatbot 1 (ChatGPT (4.1)

- **توبيراميت**
- **فالبروات الصوديوم**

✔ من أكثر الأدوية فاعلية

⚠ قد تسبب آثارًا جانبية مثل تنميل، نعاس، أو زيادة/نقصان وزن

3 مضادات الاكتئاب

- **أميتريبتيلين** (الأشهر)
- **فينلافاكسين**

✔ مفيدة خصوصًا إذا وُجد قلق أو اضطراب نوم

4 أدوية حديثة (مضادات CGRP)

- تُستخدم خصيصًا للوقاية من الشقيقة
- مثل: **إيرينوماب، فريمانيزوماب، غالكانيزوماب**

✔ فعالة جدًا وقليلة الآثار الجانبية

⚠ غالبًا مكلفة وتحتاج وصفة اختصاصي

5 البوتوكس (Botox)

- للحالات **المزمنة** (أكثر من 15 يوم صداع شهريًا)
- يُحقن كل 3 أشهر

متى يُنصح بالعلاج الوقائي؟

- إذا حدثت ≥ 4 نوبات شهريًا
- أو كانت النوبات شديدة ومعيقة
- أو فشل علاج النوبات الحادة

ملاحظات مهمة

- يحتاج الدواء الوقائي **4–8 أسابيع** ليظهر مفعوله
- الاختيار يعتمد على **العمر، الأمراض المرافقة، والآثار الجانبية**
- لا يُنصح بإيقاف الدواء فجأة دون استشارة الطبيب

إذا رغبت، أستطيع **مساعدتك في اختيار الدواء الأنسب حسب حالتك** (العمر، عدد النوبات، أمراض أخرى).

<table>
<tr><th colspan="3">Chatbot 1 (ChatGPT (4.1)</th></tr>
<tr><td>نعم، يمكن تخفيف نوبات الشقيقة (الصداع النصفي) لدى كثير من الناس بطرق منزلية أو بديلة، لكن من المهم توضيح أن هذه الطرق لا تُعد علاجًا شافيًا نهائيًا، بل وسائل للمساعدة في تقليل الشدة أو عدد النوبات، وتختلف فعاليتها من شخص لآخر.

🌿 طرق منزلية مفيدة

• الراحة في مكان هادئ ومظلم: الضوء والضجيج من أكثر محفزات الشقيقة.
• كمادات باردة أو دافئة على الرأس أو الرقبة (جرّب ما يناسبك).
• النوم المنتظم: قلة النوم أو زيادته قد يثير النوبة.
• شرب الماء: الجفاف محفز شائع.
• تنظيم الوجبات: تجنب الجوع الطويل، وبعض الأطعمة قد تحفّز الشقيقة (مثل الشوكولاتة، الأجبان المعتقة، الكافيين الزائد).

🧘 طرق بديلة أو تكميلية

• تمارين الاسترخاء والتنفس العميق.
• اليوغا أو التأمل لتقليل التوتر.
• التدليك خاصة لمنطقة الرقبة والكتفين.
• العلاج بالإبر (الوخز بالإبر): أظهر فائدة لبعض المرضى.
• الأعشاب والمكملات (بحذر وبعد استشارة طبيب):
  ○ الزنجبيل
  ○ المغنيسيوم
  ○ فيتامين 2B (الريبوفلافين)
  ○ عشبة الأقحوان (Feverfew)

📘 نصيحة مهمة

• الاحتفاظ بـ مذكرة للشقيقة لتحديد المحفزات (طعام، توتر، نوم، هرمونات).
• إذا كانت النوبات شديدة، متكررة، أو تترافق مع أعراض غير معتادة (ضعف، تشوش، تنميل)، فيجب مراجعة الطبيب.
• لا تُغني الطرق المنزلية عن العلاج الطبي في الحالات المتوسطة أو الشديدة.

إذا رغبت، يمكنني:

• اقتراح روتين يومي للوقاية
• أو مساعدتك في تحديد محفزاتك المحتملة
• أو شرح متى تكون الأدوية ضرورية

🌸</td><td>هل يمكن علاج الشقيقة بطرق منزلية أو بديلة؟</td><td>22</td></tr>
<tr><td>علاج الشقيقة (الصداع النصفي) أثناء الحمل يحتاج حذرًا لأن بعض الأدوية قد تؤثر على الجنين. الخيارات تُقسَّم عادة إلى غير دوائية ودوائية آمنة نسبيًا، مع تجنّب أدوية معيّنة.</td><td>ما خيارات علاج الشقيقة أثناء الحمل؟</td><td>23</td></tr>
</table>

Chatbot 1 (ChatGPT (4.1)		
## أولًا: خيارات غير دوائية (المفضّلة دائمًا)<br>غالبًا تكون كافية أو تقلّل الحاجة للأدوية:<br>• 🌿**الراحة والنوم المنتظم**<br>• 💧**شرب كميات كافية من السوائل**<br>• 🍽️**تناول وجبات منتظمة** لتجنّب انخفاض السكر<br>• ❄️ أو 🔥 **كمادات باردة أو دافئة** على الرأس أو الرقبة<br>• 🧘**تقنيات الاسترخاء** (تنفّس عميق، يوغا الحمل)<br>• 🚫 تجنّب المحفزات المعروفة للشقيقة (قلة النوم، روائح قوية، أطعمة معيّنة)<br>• 💆**تدليك الرقبة والكتفين**		
## ثانيًا: الأدوية المسموح بها نسبيًا (بعد استشارة الطبيب)<br>• ✅**الباراسيتامول (أسيتامينوفين)**<br>هو الخيار الأول والأكثر أمانًا خلال الحمل.<br>• ⚠️**مضادات الغثيان** (مثل ميتوكلوبراميد) قد تُستخدم إذا كان الغثيان شديدًا، ولكن بوصفة طبية.		
## ثالثًا: أدوية تُستخدم بحذر أو تُتجنّب<br>• ⚠️**مضادات الالتهاب غير الستيرويدية (مثل الإيبوبروفين)**<br>○ قد تُستخدم أحيانًا في **الثلث الثاني فقط** وتحت إشراف طبي<br>○ ❌ تُمنع في الثلث الأول والثالث<br>• ❌**التريبتانات** (مثل سوماتريبتان):<br>لا تُستخدم إلا في حالات خاصّة وتحت إشراف اختصاصي<br>• ❌**الأسبرين بجرعات عالية**<br>• ❌**الإرغوتامين**: ممنوع تمامًا أثناء الحمل		
## متى يجب مراجعة الطبيب فورًا؟<br>🚨 إذا كان الصداع:		

<table>
<tr><th colspan="3">Chatbot 1 (ChatGPT (4.1)</th></tr>
<tr><td></td><td></td><td>

شديدًا جدًا أو مختلفًا عن المعتاد
مصحوبًا بتشوّش رؤية، ضعف، تنميل، أو حمى
مفاجئًا وقويًا جدًا
مستمرًا ولا يتحسّن

<hr>
إذا أحببتِ، أستطيع:

مساعدتك بخطة وقائية للشقيقة أثناء الحمل
أو توضيح ما الذي يتغيّر حسب ثلث الحمل
أو التمييز بين الشقيقة وصداع ارتفاع ضغط الحمل (مهم جدًا)

🌸
</td></tr>
<tr><td>24</td><td>هل يمكن الشفاء من الشقيقة بشكل نهائي؟</td><td>
سؤال مهم، وكثير من المصابين بالشقيقة (الصداع النصفي) يطرحونه 🌿
الإجابة المختصرة:
👉 في أغلب الحالات لا يوجد شفاء نهائي مضمون للشقيقة، لكنها مرض يمكن السيطرة عليه بدرجة كبيرة، وقد تختفي النوبات تمامًا لفترات طويلة لدى بعض الأشخاص.
التفصيل:

الشقيقة تُعد اضطرابًا عصبيًا مزمنًا، وغالبًا لها عامل وراثي.
عند بعض الناس تخفّ حدتها أو تتوقف مع التقدم في العمر، أو بعد تغيّر ظروف معينة (مثل الحمل، انقطاع الطمث، أو تغيير نمط الحياة).
آخرون تستمر لديهم لكن بنوبات أقل وأخف.

ما الذي يساعد على التحكم بها؟

تجنب المحفزات
مثل: قلة النوم، التوتر، الجوع، بعض الأطعمة (الجبن المعتق، الشوكولاتة، الكافيين الزائد).
العلاج الدوائي

أدوية تُؤخذ أثناء النوبة لتخفيف الألم.
أدوية وقائية تُؤخذ بانتظام لتقليل عدد النوبات.

نمط الحياة

نوم منتظم
شرب ماء كافٍ
ممارسة الرياضة الخفيفة
تقنيات الاسترخاء

العلاجات الحديثة
مثل حقن البوتوكس أو أدوية حديثة تستهدف مستقبلات معينة، وقد أحدثت فرقًا كبيرًا لدى بعض المرضى.

</td></tr>
</table>

<table>
<tr><th colspan="3">Chatbot 1 (ChatGPT (4.1)</th></tr>
<tr><td></td><td></td><td>الخلاصة:
الشقيقة قد لا تُشفى نهائيًا في معظم الحالات، لكن يمكن الوصول لمرحلة لا تؤثر فيها على الحياة اليومية إطلاقًا مع العلاج والمتابعة المناسبة.

إذا أحببت، أستطيع أن أخبرك:

• هل الشقيقة عندك مع أو بدون غثيان؟
• كم مرة تأتيك النوبات؟

ذلك يساعدني أعطيك معلومات أدق تناسب حالتك.</td></tr>
<tr><td>25</td><td>كيف تؤثر الشقيقة على حياة المصابين؟</td><td>تؤثر الشقيقة (الصداع النصفي) على حياة المصابين بها بعدة طرق، وغالبًا يكون تأثيرها أعمق من مجرد صداع عابر، ومن أبرز الجوانب:

🧠 التأثير الجسدي

• نوبات صداع شديدة قد تستمر من ساعات إلى أيام.
• أعراض مرافقة مثل الغثيان، القيء، الحساسية للضوء والصوت، وأحيانًا اضطرابات بصرية (الهالة).
• الإرهاق والتعب حتى بعد انتهاء النوبة.

😔 التأثير النفسي

• القلق من موعد النوبة التالية وعدم القدرة على التنبؤ بها.
• زيادة خطر الاكتئاب أو التوتر، خاصة عند تكرار النوبات.
• شعور بالإحباط أو فقدان السيطرة.

🧑‍💼 التأثير على العمل والدراسة

• انخفاض الإنتاجية أو التغيب المتكرر.
• صعوبة التركيز أثناء النوبات أو بعدها.
• قد تؤثر على فرص التقدم الوظيفي إذا كانت النوبات متكررة وشديدة.

🧑 التأثير الاجتماعي

• الانسحاب من الأنشطة الاجتماعية أو العائلية.
• صعوبة الالتزام بالمواعيد بسبب النوبات المفاجئة.
• شعور بعدم التفهّم من الآخرين أحيانًا.

🍽️ نمط الحياة

• الحاجة لتجنب محفزات معينة مثل قلة النوم، بعض الأطعمة، التوتر، أو الإضاءة القوية.</td></tr>
</table>

Chatbot 1 (ChatGPT (4.1)		
		• الالتزام بروتين يومي أكثر انضباطًا لتقليل النوبات.<br><br>💊 **مع العلاج والدعم**<br><br>مع التشخيص الصحيح والعلاج المناسب (دوائي وغير دوائي)، وتعلّم إدارة المحفزات، يمكن لكثير من المصابين **تقليل شدة وتكرار النوبات** وتحسين جودة حياتهم بشكل ملحوظ.<br><br>إذا رغبت، أستطيع شرح **أسباب الشقيقة** أو **طرق التخفيف منها** أو **متى يجب مراجعة الطبيب**.

Chatbot 2 (Google Gemini 3 Flash Mode)		
SN	السؤال	الرد
1	ما هي الشقيقة؟	**الشقيقة** (أو الصداع النصفي - Migraine) هي أكثر من مجرد صداع شديد؛ هي اضطراب عصبي معقد يسبب نوبات من الألم النابض، وغالباً ما يتركز في جانب واحد من الرأس.<br><br>إليك أهم الحقائق حول الشقيقة بشكل مبسط:
		**الأعراض الشائعة**<br><br>لا تقتصر الشقيقة على ألم الرأس فقط، بل يصاحبها عادةً مجموعة من الأعراض:<br><br>• **ألم نابض:** شعور بـ "ضربات" أو "نبض" داخل الرأس.<br>• **الحساسية المفرطة:** انزعاج شديد من الضوء، الأصوات، أو حتى بعض الروائح.<br>• **الغثيان:** قد يصل الأمر إلى القيء في حالات كثيرة.<br>• **الأورة (:Aura)** يختبرها البعض قبل بدء الألم، وهي علامات تحذيرية مثل رؤية ومضات ضوئية، خطوط متعرجة، أو الشعور بتنميل في الوجه واليدين.
		**مراحل نوبة الشقيقة**<br><br>تمر النوبة غالباً بأربع مراحل (ليس بالضرورة أن يمر الجميع بها كلها):<br><br>1. **البادرة (:Prodrome)** تغييرات طفيفة قبل يوم أو يومين (تقلب مزاج، إمساك، رغبة في أكل

<table>
<tr><th colspan="3">Chatbot 2 (Google Gemini 3 Flash Mode)</th></tr>
<tr><td></td><td></td><td>معين).
2. الأورة (Aura): تسبق الألم مباشرة وتستمر لدقائق.
3. النوبة (Attack): وهي وقت الألم الفعلي وقد تستمر من 4 ساعات إلى 3 أيام.
4. ما بعد النوبة (Post-drome): الشعور بالإرهاق أو "الخمول" وكأن الجسم قد خاض معركة.</td></tr>
<tr><td></td><td></td><td>مسببات الشقيقة (المحفزات)
تختلف المحفزات من شخص لآخر، ومن أشهرها:
• التغيرات الهرمونية: خاصة عند النساء.
• النظام الغذائي: مثل تخطي الوجبات، الكافيين، أو الأطعمة التي تحتوي على مضافات مثل "الجلوتامات".
• الضغط النفسي: القلق والتوتر.
• تغيرات النوم: السهر الزائد أو حتى النوم أكثر من اللازم.
• العوامل البيئية: الأضواء الساطعة، الروائح القوية (مثل العطور أو الدخان).</td></tr>
<tr><td></td><td></td><td>كيف يتم التعامل معها؟
على الرغم من عدم وجود علاج نهائي للشقيقة، إلا أنه يمكن السيطرة عليها عبر:
• الأدوية: مسكنات الألم العادية، أو أدوية متخصصة يصفها الطبيب للنوبات الشديدة.
• نمط الحياة: تنظيم النوم، شرب كميات كافية من الماء، وتجنب المحفزات المعروفة.
• العلاج الوقائي: في حال كانت النوبات تتكرر بشكل كبير (أكثر من 4 مرات شهرياً).

ملاحظة هامة: إذا شعرت بصداع مفاجئ وشديد جداً (وصف بأنه "أسوأ صداع في حياتك") أو صاحب الصداع ضعف في الكلام أو الرؤية المفاجئة، يجب التوجه للطوارئ فوراً.

هل تعاني من أعراض محددة تود معرفة ما إذا كانت مرتبطة بالشقيقة، أم أن سؤالك للاطلاع العام؟</td></tr>
<tr><td>2</td><td>ما أنواع الشقيقة؟</td><td>تُعد الشقيقة (الصداع النصفي) اضطراباً عصبياً معقداً، ولا تقتصر فقط على ألم الرأس، بل تختلف أنواعها بناءً على الأعراض المصاحبة لها.

إليك قائمة بأنواع الشقيقة الرئيسية والأكثر شيوعاً:</td></tr>
<tr><td></td><td></td><td>1. الشقيقة بدون هالة (Migraine without Aura)</td></tr>
</table>

<table>
<tr><th colspan="3">Chatbot 2 (Google Gemini 3 Flash Mode)</th></tr>
<tr><td>

هذا هو النوع **الأكثر شيوعاً**، ويُعرف سابقاً باسم "الصداع النصفي العادي".

- **الأعراض:** ألم نابض في جانب واحد من الرأس، غثيان، وحساسية شديدة للضوء والصوت.
- **المدة:** تستمر النوبة عادة من 4 إلى 72 ساعة إذا لم تُعالج.

2. الشقيقة مع هالة (Migraine with Aura)

يُعرف باسم "الصداع النصفي الكلاسيكي". تسبق الألم علامات تحذيرية تسمى "الهالة".

- **أعراض الهالة:** رؤية ومضات ضوئية، خطوط متعرجة، أو فقدان مؤقت للرؤية. قد تشمل أيضاً تنميلاً في اليد أو الوجه، وصعوبة في الكلام.
- **التوقيت:** تظهر هذه العلامات عادةً قبل الصداع بـ 10 إلى 60 دقيقة.

3. الشقيقة المزمنة (Chronic Migraine)

يتم تشخيص هذا النوع عندما يعاني الشخص من الصداع لمدة **15 يوماً أو أكثر في الشهر**، لمدة لا تقل عن 3 أشهر، بحيث تكون 8 أيام منها على الأقل نوبات شقيقة كاملة الأعراض.

4. الشقيقة الدهليزية (Vestibular Migraine)

يؤثر هذا النوع على التوازن.

- **الأعراض:** دوار حاد (دوار الحركة)، فقدان التوازن، وغثيان. المثير للاهتمام أن هذا النوع قد يحدث **دون وجود ألم في الرأس** أحياناً.

5. الشقيقة البطنية (Abdominal Migraine)

ينتشر هذا النوع غالباً لدى **الأطفال**.

- **الأعراض:** ألم شديد في البطن، غثيان، وقيء. عادةً ما يتطور هؤلاء الأطفال ليصابوا بالشقيقة التقليدية عند الكبر.

6. الشقيقة العينية (Retinal Migraine)

نوع نادر وخطير يتطلب استشارة طبية فورية.

- **الأعراض:** فقدان مؤقت للرؤية أو تشوش الرؤية في **عين واحدة فقط**، ويتبع ذلك عادةً صداع نصفي.

7. شقيقة الجذع الدماغي (Basilar-type Migraine)

تنشأ أعراضها من قاعدة الدماغ، وغالباً ما تصيب النساء اليافعات.

- **الأعراض:** دوار، رؤية مزدوجة، طنين في الأذن، وصعوبة في التنسيق الحركي قبل بدء

</td><td></td><td></td></tr>
</table>

<table dir="rtl">
<tr><th colspan="3">Chatbot 2 (Google Gemini 3 Flash Mode)</th></tr>
<tr><td></td><td></td><td>
الصداع.

<hr>

مقارنة سريعة بين الأنواع الشائعة

<table dir="rtl">
<tr><th>النوع</th><th>السمة المميزة</th><th>الفئة الأكثر تأثراً</th></tr>
<tr><td>بدون هالة</td><td>ألم نابض وغثيان</td><td>أغلب المصابين</td></tr>
<tr><td>مع هالة</td><td>اضطرابات بصرية قبل الألم</td><td>25% من المصابين</td></tr>
<tr><td>الدهليزية</td><td>دوخة وفقدان توازن</td><td>من لديهم تاريخ مع دوار الحركة</td></tr>
<tr><td>البطنية</td><td>ألم في المعدة (بدون صداع غالباً)</td><td>الأطفال</td></tr>
</table>

<hr>

ملاحظة هامة: إذا شعرت بصداع مفاجئ وشديد جداً (كأنه انفجار) أو إذا تغير نمط الصداع المعتاد لديك، يجب مراجعة الطبيب فوراً.

هل تود مني مساعدتك في معرفة المثيرات الشائعة التي تؤدي لاندلاع نوبات الشقيقة وكيفية تجنبها؟
</td></tr>
<tr><td>3</td><td>هل هناك فرق بين الشقيقة والصداع النصفي؟</td><td>
في الحقيقة، لا يوجد فرق بينهما؛ فـ "الشقيقة" و"الصداع النصفي" هما مسميان لمرض واحد.

الشقيقة: هو المصطلح الشائع والقديم في التراث العربي الطبي.
الصداع النصفي (Migraine): هو المصطلح العلمي والطبي الحديث.

إليك بعض النقاط السريعة التي توضح طبيعة هذا المرض:

<h3>ما هو الصداع النصفي (الشقيقة)؟</h3>

هو اضطراب عصبي لا يقتصر فقط على ألم الرأس، بل هو "نوبة" صحية متكاملة تتميز بما يلي:

مكان الألم: غالباً ما يصيب جانباً واحداً من الرأس (ومن هنا جاءت التسمية "نصفي")، لكنه قد

</td></tr>
</table>

<table>
<tr><th colspan="3">Chatbot 2 (Google Gemini 3 Flash Mode)</th></tr>
<tr><td></td><td></td><td>
ينتقل أحياناً ليشمل الرأس كاملاً.

- **طبيعة الألم:** يكون على شكل **نبض** (خفقان) قوي.
- **الأعراض المصاحبة:**
  - حساسية شديدة للضوء والأصوات.
  - غثيان ورغبة في القيء.
  - رؤية خطوط متعرجة أو ومضات ضوئية (تسمى "الأورا" أو النسمة) قبل بدء الألم لدى بعض الأشخاص.

لماذا يختلط الأمر على البعض؟

يعتقد البعض أن "الشقيقة" حالة أشد من "الصداع النصفي"، لكن طبياً هما نفس الحالة تماماً، وتختلف شدتها من شخص لآخر ومن نوبة لأخرى.

هل تعاني من أعراض محددة تجعلك تتساءل عن الفرق؟ يمكنني مساعدتك في معرفة المحفزات الشائعة لهذا النوع من الصداع إذا أردت.
</td></tr>
<tr><td>4</td><td>ما الفرق بين الشقيقة وصداع الجيوب الأنفية؟</td><td>
التمييز بين الشقيقة (الصداع النصفي) وصداع الجيوب الأنفية قد يكون مربكاً جداً، لأن كلاهما يسبب ألماً في الوجه والجبهة، بل إن الشقيقة قد تسبب أحياناً "سيلان الأنف".

إليك أهم الفروقات الجوهرية التي تساعدك على التمييز بينهما:

1. نوع الألم ومكانه

<table>
<tr><th>وجه المقارنة</th><th>الشقيقة (الصداع النصفي)</th><th>صداع الجيوب الأنفية</th></tr>
<tr><td>طبيعة الألم</td><td>ألم نابض (مثل دقات القلب) وعادة ما يكون شديداً.</td><td>ضغط ثابت ووزن ثقيل في الوجه.</td></tr>
<tr><td>الموقع</td><td>غالباً في جانب واحد من الرأس، لكنه قد ينتقل.</td><td>حول الوجنتين، الجبهة، أو جسر ال[illegible] الجانبين عادة).</td></tr>
</table>
</td></tr>
</table>

Chatbot 2 (Google Gemini 3 Flash Mode)

**تأثير الحركة**	يزداد سوءاً مع **النشاط البدني** العادي (كالمشي).	يزداد سوءاً عند **الانحناء للأمام** أو الاستلقاء.

2. الأعراض المرافقة (العلامات الفاصلة)

تعتبر هذه الأعراض هي "المفتاح" للتشخيص الصحيح:

- **في الشقيقة:**
  - **الحساسية:** انزعاج شديد من الضوء القوي، الأصوات العالية، أو الروائح.
  - **الجهاز الهضمي:** الشعور بالغثيان أو الرغبة في التقيؤ.
  - **الهالة (Aura):** رؤية ومضات ضوئية أو خطوط زجزاجية قبل بدء الألم.
  - **الإفرازات:** إذا وجد سيلان أنف، فإنه يكون **شفافاً ومائياً**.
- **في صداع الجيوب الأنفية:**
  - **الإفرازات:** وجود مخاط **سميك وملون** (أصفر أو أخضر).
  - **الحرارة:** قد يصاحبه ارتفاع في درجة الحرارة (حمى).
  - **الحواس:** ضعف ملحوظ في حاسة الشم.
  - **أوجاع أخرى:** ألم في الأسنان العلوية أو رائحة فم كريهة نتيجة الالتهاب.

3. مدة النوبة

- **الشقيقة:** تدوم عادة من **4 ساعات إلى 3 أيام**، وتختفي تماماً بين النوبات.
- **صداع الجيوب:** يستمر **لأيام طويلة أو أسابيع** ولا يزول إلا بعلاج الالتهاب الأساسي (غالباً بعد نزلة برد أو إنفلونزا).

معلومة هامة: تشير الدراسات الطبية إلى أن ما يقرب من **80-90%** من الأشخاص الذين يعتقدون أنهم مصابون بصداع الجيوب الأنفية، هم في الواقع يعانون من "شقيقة" تسبب أعراضاً في منطقة الجيوب.

هل تعاني من هذا الألم حالياً؟ وإذا كان الأمر كذلك، هل يزداد سوءاً عندما تنظر إلى شاشة الهاتف أو تتعرض لإضاءة قوية؟

5	هل صداع الشقيقة خطير؟	بشكل عام، **صداع الشقيقة (المهاجرين) بحد ذاته ليس مرضاً يهدد الحياة أو "خطيراً" بالمعنى القاتل،** لكنه يُصنف كحالة عصبية قد تكون "منهكة" وتؤثر بشكل كبير على جودة الحياة.

		Chatbot 2 (Google Gemini 3 Flash Mode)
		ومع ذلك، هناك حالات ومضاعفات معينة تتطلب اهتماماً طبياً، إليك التفصيل:<br><br>**1. متى تكون الشقيقة مقلقة؟**<br><br>تصبح الشقيقة "خطيرة" أو تستدعي القلق في حالات نادرة أو عند ظهور أعراض غير معتادة، مثل:<br>• **الحالة الشقيقية (**:**Status Migrainosus)** وهي نوبة صداع شديدة تستمر لأكثر من 72 ساعة ولا تستجيب للأدوية المعتادة، مما قد يؤدي إلى الجفاف بسبب القيء المستمر.<br>• **السكتة الشقيقية (**:**Migrainous Infarction)** حالة نادرة جداً حيث تحدث سكتة دماغية أثناء نوبة الشقيقة المصحوبة بـ "أورا" (علامات تحذيرية بصرية أو حسية).<br>• **تغير نمط الصداع:** إذا كنت معتاداً على نمط معين وتغير فجأة ليصبح أشد أو يرافقه أعراض جديدة لم تعهدها.<br><br>**2. أعراض "العلم الأحمر" (تستوجب زيارة الطوارئ فوراً):**<br><br>إذا شعرت بأي من الأعراض التالية، فلا تتعامل معها كصداع عادي وتوجه للطبيب فوراً:<br>• صداع مفاجئ وشديد جداً (يوصف بأنه "أسوأ صداع في حياتك" أو الصداع الرعدي).<br>• فقدان مفاجئ للرؤية أو ازدواجية الرؤية.<br>• ضعف أو تنميل في جانب واحد من الجسم.<br>• صعوبة في الكلام أو ارتباك ذهني.<br>• صداع مصحوب بحمى شديدة أو تصلب في الرقبة (قد يشير لالتهاب السحايا).<br>• صداع يبدأ لأول مرة بعد سن الخمسين.<br><br>**3. التأثير على جودة الحياة**<br><br>رغم أنها ليست مميتة، إلا أن خطورتها تكمن في **"الإعاقة المؤقتة"**؛ فهي تمنع الشخص من ممارسة عمله أو حياته الاجتماعية، وقد تؤدي مع مرور الوقت إلى مضاعفات نفسية مثل الاكتئاب والقلق بسبب تكرار الألم.<br><br>**نصيحة:** إذا كانت نوبات الشقيقة تتكرر لديك أكثر من مرتين في الأسبوع، أو إذا كنت تستهلك المسكنات بكثرة، فمن الضروري استشارة طبيب مخ وأعصاب لوضع خطة علاجية وقائية.<br><br>**هل تعاني من أعراض معينة تثير قلقك حالياً، مثل زغللة العين أو تنميل الأطراف؟**
6	ما هي أعراض الشقيقة؟ وما هي المراحل التي قد تمر بها النوبة؟	الشقيقة (أو الصداع النصفي) ليست مجرد صداع عابر، بل هي اضطراب عصبي معقد يمر غالباً بأربع مراحل متميزة، ورغم أن ليس كل المصابين يمرون بجميع هذه المراحل، إلا أن فهمها يساعد في التعامل مع النوبة بشكل أفضل.<br><br>إليك تفصيل لأعراض الشقيقة ومراحلها:

Chatbot 2 (Google Gemini 3 Flash Mode)		
**1. مرحلة البادرة (Prodrome)**<br>تبدأ قبل النوبة بيوم أو يومين، وهي بمثابة "إنذار مبكر" للجسم. من أهم أعراضها:<br>● **تغيرات مزاجية:** الشعور بالكآبة المفاجئة أو الحيوية المفرطة.<br>● **تغيرات جسدية:** تيبس في الرقبة، كثرة التثاؤب، أو الإمساك.<br>● **الرغبة في الطعام:** اشتهاء أنواع معينة من الأطعمة (مثل الشوكولاتة).<br>● **زيادة العطش:** والحاجة المتكررة للتبول.		
**2. مرحلة "الهالة" أو الأورة (Aura)**<br>تحدث لدى حوالي 25% من المصابين فقط، وتسبق الصداع مباشرة أو ترافقه، وتستمر عادةً من 20 إلى 60 دقيقة. تشمل:<br>● **اضطرابات بصرية:** رؤية ومضات من الضوء، بقع ساطعة، أو خطوط متعرجة.<br>● **أعراض حسية:** الشعور بوخز "إبر" في الذراع أو الساق، أو تنميل في جانب واحد من الوجه.<br>● **مشاكل في النطق:** صعوبة في العثور على الكلمات أو التحدث بوضوح.		
**3. مرحلة النوبة (Attack)**<br>وهي المرحلة المركزية التي يظهر فيها الصداع الفعلي، وقد تستمر من 4 ساعات إلى 3 أيام. أعراضها:<br>● **الألم:** غالباً ما يكون في جانب واحد من الرأس، ويُوصف بأنه **ألم نابض** أو خافق.<br>● **الحساسية الشديدة:** انزعاج كبير من الضوء، الأصوات، وحتى بعض الروائح.<br>● **مشاكل هضمية:** غثيان شديد قد يصل إلى القيء.<br>● **تفاقم الألم:** يزداد الوجع سوءاً مع أي نشاط بدني بسيط كالمشي أو صعود الدرج.		
**4. مرحلة ما بعد النوبة (Postdrome)**<br>تسمى أحياناً "مرحلة مخلفات الصداع"، حيث يشعر الشخص بالإرهاق التام بعد انتهاء الألم، وتشمل:<br>● الشعور بالاستنزاف الجسدي والذهني.<br>● دوخة خفيفة أو تشوش في التركيز.<br>● في حالات نادرة، قد يشعر البعض بابتهاج مفاجئ لانتهاء الألم.		
**متى يجب زيارة الطبيب فوراً؟**<br>هناك علامات "حمراء" تستدعي الطوارئ لأنها قد تشير لمشاكل أعمق:		

Chatbot 2 (Google Gemini 3 Flash Mode)		
		1. صداع مفاجئ وشديد جداً (يوصف بأنه أسوأ صداع في حياتك).<br>2. صداع مصحوب بحمى، تيبس شديد في الرقبة، أو تشوش ذهني.<br>3. فقدان مفاجئ للرؤية أو ضعف في أحد جانبي الجسم.<br><br>**هل تود أن أساعدك في إعداد قائمة بالمحفزات الشائعة التي قد تثير نوبات الشقيقة لديك لتجنبها؟**
7	ما هي الهالة (الأورة) في الشقيقة؟	تُعد **الهالة (الأورة - Aura)** من أكثر الظواهر إثارة للاهتمام في عالم الطب، وهي عبارة عن مجموعة من الأعراض العصبية المؤقتة التي تسبق عادةً نوبة الصداع النصفي (الشقيقة) أو ترافقه.<br><br>حوالي **25% إلى 30%** من المصابين بالشقيقة يمرون بهذه التجربة، وتُسمى حالتهم بـ "الشقيقة المصحوبة بهالة".<br><br>إليك تفصيل لمظاهرها وأسبابها:<br><br>**1. أنواع أعراض الهالة**<br><br>لا تقتصر الهالة على الرؤية فقط، بل يمكن أن تشمل حواساً أخرى:<br>● **اضطرابات بصرية (الأكثر شيوعاً):**<br>○ رؤية بقع عمياء (سكوتوما).<br>○ رؤية خطوط متعرجة تشبه "الحصون القديمة".<br>○ ومضات ضوئية أو أضواء ساطعة.<br>○ فقدان مؤقت للرؤية في جانب واحد.<br>● **أعراض حسية:**<br>○ الشعور بـ "تنميل" أو "وخز" (مثل الإبر) يبدأ عادةً في اليد وينتقل إلى الذراع ثم الوجه.<br>● **اضطرابات الكلام:**<br>○ صعوبة في إيجاد الكلمات أو التحدث بوضوح (حبسة كلامية مؤقتة).<br>● **أعراض حركية (نادرة):**<br>○ ضعف في طرف واحد من الجسم (تُعرف بالشقيقة الفالجية).<br><br>**2. الخصائص الزمنية**<br><br>تتميز الهالة بجدول زمني محدد يساعد الأطباء على تشخيصها:<br>● **التدرج:** تبدأ الأعراض تدريجياً على مدار 5 إلى 20 دقيقة.<br>● **المدة:** عادة ما تستمر الهالة لفترة تتراوح بين **20 إلى 60 دقيقة**.<br>● **التتابع:** غالباً ما يتبع الهالة صداع شديد في غضون ساعة، لكن في بعض الأحيان (خاصة عند

Chatbot 2 (Google Gemini 3 Flash Mode)		
كبار السن) قد تحدث الهالة **دون** أن يتبعها صداع.		
**3. لماذا تحدث الهالة؟**<br><br>النظرية العلمية الرائدة حالياً هي ما يسمى **"الاكتئاب المنتشر في القشرة المخية" (Cortical Spreading Depression)**. وهي عبارة عن موجة من النشاط الكهربائي تتبعها موجة من الخمول تنتشر عبر قشرة الدماغ. هذه الموجة هي التي تسبب الأعراض العصبية (مثل الأضواء المتعرجة) قبل أن تحفز أعصاب الألم وتؤدي للصداع.		
**4. متى يجب القلق؟**<br><br>بما أن أعراض الهالة (مثل التنميل أو صعوبة الكلام) تشبه إلى حد كبير أعراض **السكتة الدماغية**، يجب استشارة الطبيب فوراً إذا:<br>1. ظهرت هذه الأعراض لأول مرة في حياتك.<br>2. استمرت الأعراض لأكثر من ساعة.<br>3. حدث ضعف شديد في جانب واحد من الجسم لم تعهده من قبل.		
**ملاحظة مهمة:** المصابون بالشقيقة مع هالة (خاصة النساء) يُنصحون عادةً بتجنب التدخين وبعض أنواع حبوب منع الحمل لأنها قد تزيد من خطر الإصابة بالسكتات الدماغية بشكل طفيف.<br><br>هل تظهر لديك هذه الأعراض حالياً، أم أنك تسأل لفهم الحالة بشكل عام؟ يمكنني مساعدتك في معرفة كيفية التعامل مع النوبة عند بدايتها.		
نعم، يُعتبر **الغثيان والقيء** من الأعراض الشائعة والأساسية للصداع النصفي (الشقيقة)، وليست مجرد أعراض جانبية.<br><br>في الواقع، يعاني حوالي **80%** من المصابين بالشقيقة من الغثيان، بينما يتعرض حوالي **50%** منهم للقيء الفعلي أثناء نوبة الصداع.<br><br>إليك بعض التفاصيل المهمة حول هذه الأعراض:	هل القيء والغثيان من أعراض الشقيقة؟	8
**لماذا يحدث الغثيان مع الشقيقة؟**<br><br>يعتقد الأطباء أن السبب يعود إلى تأثير الشقيقة على الجهاز العصبي، مما يؤدي إلى تباطؤ في عملية		

<table>
<tr><th colspan="3">Chatbot 2 (Google Gemini 3 Flash Mode)</th></tr>
<tr><td>
الهضم (ما يسمى "الركود المعدي"). هذا التباطؤ هو الذي يسبب الشعور باللوعة أو الرغبة في التقيؤ.

خصائص غثيان الشقيقة:
• التوقيت: قد يبدأ الغثيان قبل بدء ألم الرأس (كجزء من "البادرة") أو يظهر مع اشتداد الألم.
• الحساسية: غالباً ما يزداد الغثيان سوءاً عند التعرض للروائح القوية، الأضواء الساطعة، أو الحركة.
• التأثير على العلاج: القيء قد يمنع الجسم من امتصاص الأدوية المسكنة التي تؤخذ عن طريق الفم، مما يجعل السيطرة على النوبة أصعب.

متى يجب استشارة الطبيب فوراً؟
رغم أن الغثيان طبيعي مع الشقيقة، إلا أن هناك حالات تستدعي القلق، مثل:
• إذا كان القيء شديداً لدرجة تمنعك من شرب السوائل (خطر الجفاف).
• إذا كان الصداع مفاجئاً وعنيفاً جداً (يسمى "صداع الرعد").
• إذا رافق القيء تصلب في الرقبة أو حمى.
<hr>
نصيحة سريعة: إذا كنت تعاني من القيء المتكرر أثناء النوبات، قد يقترح عليك الطبيب أدوية تؤخذ عن طريق الحقن أو بخاخات الأنف أو أقذراص تذوب تحت اللسان لتجاوز مشكلة الهضم.

هل تلاحظ أن الغثيان يبدأ لديك قبل الصداع أم بعد أن يشتد الألم؟ يمكنني مساعدتك في معرفة كيفية التعامل مع النوبة بناءً على نمطها.
</td><td></td><td></td></tr>
<tr><td>
نعم، هناك علاقة وثيقة ومثبتة طبياً بين الشقيقة (المهاجرين) وبين كل من آلام الرقبة وطنين الأذن. في الواقع، يرى الكثير من الأطباء أن هذه الأعراض ليست مجرد آثار جانبية، بل هي جزء لا يتجزأ من نوبة الشقيقة لدى الكثير من المرضى.

إليك توضيح لكيفية ارتباط هذه الأعراض بالشقيقة:
<hr>
1. آلام الرقبة والشقيقة
تعتبر آلام الرقبة من أكثر الأعراض المصاحبة للشقيقة شيوعاً، بل إنها أحياناً تسبق الشعور بالصداع.
• العصب الثلاثي التوائم: هناك تداخل في المسارات العصبية بين الرقبة والرأس. عندما تتهيج الأعصاب في أعلى العمود الفقري العنقي، يمكن أن ينتقل الألم إلى الرأس والعكس صحيح.
• التشنج العضلي: الإجهاد والتوتر الناتج عن نوبة الألم يؤدي غالباً إلى تشنج عضلات الرقبة والكتفين، مما يزيد من حدة النوبة.
• خرافة "صداع الرقبة": الكثير من الناس يشخصون أنفسهم بأن لديهم "صداع من الرقبة"، بينما
</td><td>هل طنين الأذن أو آلام الرقبة لها علاقة بالشقيقة؟</td><td>9</td></tr>
</table>

Chatbot 2 (Google Gemini 3 Flash Mode)		
هم في الحقيقة يعانون من شقيقة تظهر آلامها في منطقة الرقبة.<br><br>**2. طنين الأذن والشقيقة**<br><br>يوجد نوع محدد من الشقيقة يرتبط ارتباطاً مباشراً بالأذن والتوازن:<br>• **الشقيقة الدهليزية (:(Vestibular Migraine** في هذا النوع، قد لا يكون الصداع هو العرض الرئيسي، بل يشكو المريض من طنين، دوار، وثقل في الأذن.<br>• **الحساسية الحسية:** خلال نوبة الشقيقة، يصبح الدماغ شديد الحساسية للمدخلات الحسية. وكما ينزعج المريض من الضوء (رهاب الضوء)، قد يترجم الدماغ الإشارات العصبية للأذن كطنين أو صفير.<br>• **التغيرات الوعائية:** التغيرات في تدفق الدم التي تحدث أثناء الشقيقة قد تؤثر على الأذن الداخلية، مما يسبب طنيناً مؤقتاً يزول بزوال النوبة.<br><br>**متى يجب استشارة الطبيب؟**<br><br>على الرغم من أن هذه الأعراض شائعة مع الشقيقة، إلا أنه يفضل مراجعة الطبيب في الحالات التالية:<br>1. إذا كان الطنين مستمراً في أذن واحدة فقط ولا يختفي.<br>2. إذا كان ألم الرقبة مصحوباً بتنميل أو ضعف في الذراعين.<br>3. إذا تغير نمط الصداع المعتاد لديك بشكل مفاجئ.<br><br>**ملاحظة:** علاج الشقيقة الأساسي غالباً ما يؤدي إلى تحسن ملحوظ في آلام الرقبة والطنين المرتبط بها.<br><br>هل ترغب في أن أساعدك في إعداد قائمة بالأسئلة التي يمكنك طرحها على طبيبك لتحديد ما إذا كانت هذه الأعراض مرتبطة بنوع معين من الشقيقة؟		
تُعد الشقيقة (الصداع النصفي) حالة عصبية معقدة لا تقتصر فقط على "ألم في الرأس"، بل هي اضطراب يؤثر على الجهاز العصبي والحواس بشكل عام. حتى الآن، لا يوجد سبب واحد محدد، لكن العلم يرجح تداخل عدة عوامل.<br><br>إليك توضيح شامل للأسباب الكامنة والمحفزات اليومية:<br><br>**أولاً: أسباب الشقيقة (لماذا تحدث؟)**<br><br>الأسباب الحقيقية لا تزال قيد البحث، ولكن التفسيرات العلمية الحالية تركز على:	ما أسباب الشقيقة؟ وما هي محفزات النوبات؟	10

Chatbot 2 (Google Gemini 3 Flash Mode)

- **النشاط العصبي غير الطبيعي:** يُعتقد أن "موجة" من النشاط العصبي تنتشر عبر الدماغ، مما يؤدي إلى تغيرات في تدفق الدم وتنشيط الأعصاب المرتبطة بالألم.
- **اختلال المواد الكيميائية (السيروتونين):** انخفاض مستويات السيروتونين في الدماغ يؤدي إلى توسع الأوعية الدموية والتهاب الأعصاب، مما يسبب الألم النبضي المعروف.
- **العوامل الوراثية:** تلعب الوراثة دوراً كبيراً؛ فإذا كان أحد الوالدين يعاني من الشقيقة، فهناك احتمالية تتراوح بين **50% إلى 75%** أن يصاب بها الأبناء.
- **تغيرات في جذع الدماغ:** تفاعل جذع الدماغ مع العصب الثلاثي التوائم (Trigeminal nerve)، وهو مسار رئيسي للألم، يعتبر أحد المسببات الأساسية.

ثانياً: محفزات نوبات الشقيقة (ما الذي يبدأ الألم؟)

المحفزات ليست أسباباً للمرض نفسه، بل هي "شرارات" تشعل النوبة لدى الأشخاص المصابين أصلاً. تختلف هذه المحفزات من شخص لآخر:

1. المحفزات الهرمونية

خاصة عند النساء، حيث ترتبط النوبات بتذبذب مستويات **الإستروجين** (قبل أو أثناء الدورة الشهرية، أو خلال فترة انقطاع الطمث).

2. المحفزات الغذائية

- **الأطعمة المالحة والمعالجة:** مثل اللحوم الباردة التي تحتوي على النترات.
- **المضافات الغذائية:** مثل محلي "الأسبارتام" أو معزز النكهة "غلوتامات أحادية الصوديوم" (MSG).
- **المشروبات:** الإفراط في الكافيين (أو الانقطاع المفاجئ عنه) والمشروبات الغازية.

3. العوامل البيئية والحسية

- **الأضواء الساطعة:** ضوء الشمس القوي أو الأضواء الوامضة (Flickering lights).
- **الروائح القوية:** مثل العطور، والمنظفات، ودخان السجائر.
- **الأصوات العالية.**

4. نمط الحياة والضغوط

- **التوتر النفسي:** الضغط في العمل أو المنزل يعد من أشهر المحفزات.
- **اضطرابات النوم:** قلة النوم أو حتى النوم الزائد عن الحاجة (خمول عطلة نهاية الأسبوع).
- **المجهود البدني الشاق:** التمارين العنيفة المفاجئة دون تدرج.

نصيحة "مفكرة الصداع"

<table>
<tr><th colspan="3">Chatbot 2 (Google Gemini 3 Flash Mode)</th></tr>
<tr><td></td><td></td><td>بما أن المحفزات تختلف من شخص لآخر، يُنصح دائماً ببدء تدوين "مفكرة للشقيقة"، تسجل فيها:
1. متى بدأت النوبة؟
2. ماذا أكلت قبلها؟
3. كيف كانت جودة نومك؟
4. حالة الطقس أو الضغوط النفسية.

هل تود مني مساعدتك في وضع نموذج بسيط لهذه المفكرة لتتبع نوباتك بشكل أدق؟</td></tr>
<tr><td>11</td><td>هل الشقيقة مرض وراثي ومن هم الأكثر عرضة للإصابة؟</td><td>نعم، تُصنف الشقيقة (أو الصداع النصفي) كمرض عصبي وراثي بشكل كبير. فالدراسات تشير إلى أن الجينات تلعب دوراً أساسياً في تحديد مدى حساسية دماغك لبعض المحفزات.

إليك التفاصيل حول الجانب الوراثي والفئات الأكثر عرضة للإصابة:

1. هل هي وراثية؟
• التاريخ العائلي: أكثر من 80% من المصابين بالشقيقة لديهم أقارب من الدرجة الأولى يعانون من الحالة نفسها.
• نسبة الاحتمالية: إذا كان أحد الوالدين مصاباً، فإن احتمال إصابة الأبناء يصل إلى 50%. أما إذا كان كلا الوالدين مصابين، فقد ترتفع النسبة لتصل إلى 75%.
• آلية الوراثة: لا تورث الشقيقة عبر جين واحد فقط في أغلب الحالات، بل هي نتيجة تفاعل مجموعة من الجينات التي تؤثر على كيفية تواصل الخلايا العصبية ومعالجة الدماغ للألم.
<hr>2. من هم الأكثر عرضة للإصابة؟
هناك فئات معينة تزداد لديها احتمالية الإصابة بناءً على عوامل بيولوجية ونمط الحياة:
• النساء: هن الأكثر عرضة للإصابة بثلاثة أضعاف مقارنة بالرجال، ويرجع ذلك بشكل رئيسي إلى التغيرات الهرمونية (مثل تقلبات الأستروجين خلال الدورة الشهرية، الحمل، أو انقطاع الطمث).
• الفئة العمرية (15 - 50 سنة): رغم أنها قد تبدأ في أي عمر، إلا أنها غالباً ما تظهر لأول مرة في سن المراهقة وتصل لذروتها في الثلاثينيات، ثم تصبح أقل حدة بعد سن الخمسين.
• أصحاب نمط الحياة المجهد: الأشخاص الذين يعانون من مستويات عالية من التوتر النفسي، أو الذين يعانون من اضطرابات في النوم (الأرق أو النوم الزائد).
• المصابون ببعض الحالات الصحية: هناك ارتباط بين الشقيقة وبين حالات مثل القلق، الاكتئاب، واضطرابات النوم.
<hr>محفزات قد تزيد من ظهور النوبات:</td></tr>
</table>

		Chatbot 2 (Google Gemini 3 Flash Mode)
		حتى مع وجود الاستعداد الوراثي، غالباً ما تحتاج الشقيقة إلى "محفز" لبدء النوبة، ومن أشهرها:<br>1. **الأطعمة:** مثل الأجبان المعتقة، الشوكولاتة، والمشروبات التي تحتوي على الكافيين.<br>2. **المحفزات الحسية:** الأضواء الساطعة، الأصوات العالية، والروائح النفاذة (كالعطور أو الدخان).<br>3. **تغيرات الجو:** مثل الرطوبة العالية أو التغير المفاجئ في درجات الحرارة.<br><br>**نصيحة:** إذا كانت النوبات تتكرر لديك بشكل يعيق حياتك اليومية، يُفضل الاحتفاظ بـ "مفكرة للصداع" لتحديد المحفزات الخاصة بك ومناقشتها مع الطبيب.<br><br>**هل تود مساعدتي في إعداد نموذج بسيط لجدول تتبع نوبات الصداع والمحفزات لمشاركته مع طبيبك؟**
12	ما الأطعمة التي قد تحفز نوبات الشقيقة؟	تعد "محفزات الطعام" من أكثر العوامل شيوعاً لنوبات الشقيقة (الصداع النصفي)، لكن من المهم معرفة أن هذه المحفزات تختلف بشكل كبير من شخص لآخر. ما قد يسبب نوبة لشخص ما، قد يكون آمناً تماماً لآخر.<br><br>إليك قائمة بالأطعمة والمشروبات الأكثر شيوعاً التي قد تحفز الشقيقة:<br><br>**1. الأطعمة التي تحتوي على التيرامين (Tyramine)**<br>التيرامين مادة كيميائية تتكون عند تكسر البروتينات في الأطعمة مع مرور الوقت (التعتيق).<br>• **الأجبان المعتقة:** مثل الشيدر، والبارميزان، والجبنة الزرقاء (بلو شيز).<br>• **اللحوم المصنعة:** مثل النقانق، السلامي، والبسطرمة (تحتوي أيضاً على النترات).<br>• **المخللات والمخمرات:** مثل الكرنب المخلل والزيتون.<br><br>**2. الكافيين والكحول**<br>• **الكافيين:** هو سلاح ذو حدين؛ فكمية قليلة قد تخفف الألم، لكن **الإفراط فيه** أو **الانسحاب المفاجئ** منه (مثل عدم شرب قهوة الصباح المعتادة) يعد محفزاً قوياً.<br>• **المشروبات الكحولية:** خاصة النبيذ الأحمر، لاحتوائه على التانينات والفلافونويد.<br><br>**3. المضافات الغذائية**<br>• **مادة MSG (أحادية غلوتامات الصوديوم):** توجد بكثرة في الأكل الصيني، والوجبات السريعة، وبعض أنواع رقائق البطاطس (الشيبس).<br>• **المحليات الصناعية:** مثل "الأسبارتام" الموجود في المشروبات الغازية المخصصة للريجيم (Diet).<br>• **النترات والنتريت:** المواد الحافظة الموجودة في اللحوم الباردة والمجمدة.

<table>
<tr><th colspan="3">Chatbot 2 (Google Gemini 3 Flash Mode)</th></tr>
<tr><td></td><td></td><td>
<h3>4. أطعمة أخرى شائعة</h3>

الشوكولاتة: يعتقد البعض أنها محفز، لكن بعض الدراسات تشير إلى أن الرغبة في أكل الشوكولاتة قد تكون هي "العرض الأول" لبداية النوبة وليست السبب.
الأطعمة الباردة جداً: مثل الآيس كريم، خاصة إذا تم تناولها بسرعة (صداع الآيس كريم).
الحمضيات والمكسرات: قد تؤثر على نسبة صغيرة من المصابين.

<hr>
<h3>نصائح للتعرف على محفزاتك الشخصية:</h3>
<table>
<tr><th>الإجراء</th><th>الهدف</th></tr>
<tr><td>مفكرة الصداع</td><td>سجل ما أكلته قبل بدء النوبة بـ 24 ساعة لتكتشف النمط المتكرر.</td></tr>
<tr><td>تنظيم الوجبات</td><td>الجوع وانخفاض سكر الدم محفز أقوى من نوع الطعام نفسه أحياناً.</td></tr>
<tr><td>شرب الماء</td><td>الجفاف هو أحد أكبر محفزات الشقيقة عالمياً.</td></tr>
</table>
ملاحظة: لا تمنع نفسك من كل هذه الأطعمة دفعة واحدة، بل حاول مراقبة تأثير كل صنف على حدة لضمان استمتاعك بنظام غذائي متنوع.
هل تلاحظ أن نوعاً معيناً من الطعام يرتبط دائماً بصداعك، أم أن نوباتك تأتي بشكل مفاجئ؟ يمكنني مساعدتك في تصميم جدول لمتابعة محفزاتك.
</td></tr>
<tr><td>13</td><td>كيف يتم تشخيص الشقيقة؟</td><td>
تشخيص الشقيقة (الصداع النصفي) ليس عملية تعتمد على فحص دم أو تصوير إشعاعي محدد، بل يعتمد بشكل أساسي على التاريخ الطبي الدقيق ووصف الأعراض للطبيب.
إليك الخطوات المتبعة عادةً للوصول إلى التشخيص:
<h3>1. المعايير السريرية (وصف الألم)</h3>
يعتمد الأطباء على معايير الجمعية الدولية للصداع، حيث يتم تشخيص الشقيقة إذا تكررت النوبات (5 مرات على الأقل) وتوفرت فيها الخصائص التالية:
</td></tr>
</table>

Chatbot 2 (Google Gemini 3 Flash Mode)

- **موقع الألم:** غالباً ما يكون في جانب واحد من الرأس.
- **طبيعة الألم:** نبضي أو خافق (مثل ضربات القلب).
- **الشدة:** ألم متوسط إلى شديد يعيق ممارسة الأنشطة اليومية.
- **المحفزات الحركية:** يزداد الألم سوءاً مع المجهود البدني (مثل المشي أو صعود الدرج).
- **الأعراض المصاحبة:** الغثيان أو القيء، والحساسية الشديدة للضوء والأصوات.

2. التاريخ المرضي المفصل

سيسألك الطبيب أسئلة محددة لتحديد النمط، مثل:

- متى بدأت الصداع؟ وما هي مدة النوبة (عادة من 4 إلى 72 ساعة)؟
- هل تشعر بعلامات تحذيرية قبل الألم (ما يسمى بـ **الهالة** أو Aura)، مثل رؤية ومضات ضوئية أو تنميل في اليد؟
- هل هناك تاريخ عائلي للإصابة بالشقيقة؟

3. الفحص البدني والعصبي

يقوم الطبيب بإجراء فحص للتأكد من سلامة الجهاز العصبي، ويشمل ذلك:

- اختبار ردود الفعل (Reflexes).
- قوة العضلات والتوازن.
- فحص النظر والاستجابة للضوء.

4. الفحوصات التصويرية (للاستبعاد فقط)

لا تُستخدم الأشعة لتشخيص الشقيقة نفسها، ولكن قد يطلب الطبيب **الرنين المغناطيسي (MRI)** أو **الأشعة المقطعية (CT scan)** في حالات معينة لاستبعاد أسباب أخرى للصداع، مثل:

- إذا كان هذا أسوأ صداع شعرت به في حياتك فجأة.
- إذا تغير نمط الصداع المعتاد لديك بشكل جذري.
- وجود أعراض عصبية غير مفسرة (مثل ضعف مفاجئ أو فقدان توازن دائم).

نصيحة "مفكرة الصداع"

أفضل أداة لمساعدة طبيبك هي الاحتفاظ بـ **مفكرة للصداع** لمدة أسبوعين، تدون فيها:

1. وقت بدء الصداع ونهايته.
2. نوع الطعام الذي تناولته قبل النوبة.
3. جودة نومك وحالتك النفسية.
4. الأدوية التي تناولتها ومدى فعاليتها.

هل تود مني مساعدتك في إعداد قائمة بالأسئلة التي يمكنك طرحها على طبيبك في موعدك القادم؟

<table>
<tr><th colspan="3">Chatbot 2 (Google Gemini 3 Flash Mode)</th></tr>
<tr><td></td><td></td><td></td></tr>
<tr><td>14</td><td>ما مدى شيوع الشقيقة؟</td><td>تُعد الشقيقة (الصداع النصفي) واحدة من أكثر الحالات الصحية انتشاراً في العالم، وهي لا تُصنف مجرد "صداع عادي"، بل هي اضطراب عصبي يؤثر على حياة مئات الملايين.

إليك نظرة على مدى شيوعها بالأرقام والحقائق:

1. الانتشار العالمي
• المرتبة: تُصنف الشقيقة كـ ثالث أكثر الأمراض انتشاراً في العالم.
• النسبة المئوية: تشير التقديرات إلى أن حوالي 12% إلى 15% من سكان العالم يعانون من الشقيقة.
• العدد الإجمالي: هذا يعني وجود أكثر من مليار إنسان حول العالم يعيشون مع هذا المرض.

2. التوزيع حسب الجنس
تُصيب الشقيقة النساء بشكل أكبر بكثير من الرجال نتيجة التغيرات الهرمونية:
• النساء: تُصاب 1 من كل 4 نساء تقريباً خلال حياتهن.
• الرجال: يُصاب 1 من كل 11 رجلاً تقريباً.

3. الفئات العمرية
• تبدأ الشقيقة غالباً في سن المراهقة أو الشباب، وتصل لذروتها في "سن الإنتاج" (بين 30 و 50 عاماً).
• لا تقتصر على البالغين فقط، بل تصيب حوالي 10% من الأطفال والمراهقين.

4. الشقيقة المزمنة
بينما يعاني معظم الناس من نوبات متقطعة، هناك حوالي 1% إلى 2% من سكان العالم يعانون من "الشقيقة المزمنة"، وهي الحالة التي يأتي فيها الصداع لمدة 15 يوماً أو أكثر في الشهر الواحد.<hr>لماذا هذه الأرقام مهمة؟

تكمن أهمية هذه الإحصائيات في إظهار مدى "تعطيل" هذا المرض للحياة اليومية؛ حيث تُصنف منظمة الصحة العالمية (WHO) الشقيقة كأحد أكثر الأسباب المؤدية للإعاقة الجسدية وتقليل جودة الحياة عالمياً.

هل تعاني أنت أو شخص تعرفه من أعراض معينة تود التأكد من علاقتها بالشقيقة؟ يمكنني مساعدتك في فهم الفرق بينها وبين أنواع الصداع الأخرى.</td></tr>
<tr><td>15</td><td>في أي عمر تبدأ</td><td>تبدأ نوبات الشقيقة (الصداع النصفي) عادةً في مرحلة الشباب أو المراهقة، ولكن من الممكن أن تظهر في</td></tr>
</table>

<table>
<tr><th colspan="3">Chatbot 2 (Google Gemini 3 Flash Mode)</th></tr>
<tr>
<td></td>
<td>نوبات الشقيقة؟</td>
<td>أي وقت خلال حياة الشخص.

إليك تفصيل للفئات العمرية وكيفية ظهورها:

1. الطفولة (قبل البلوغ)
قد تبدأ الشقيقة لدى الأطفال في عمر مبكر جداً (حتى سن 5 سنوات). غالباً ما تظهر في هذا العمر بشكل غير تقليدي، مثل:
• نوبات الدوار.
• آلام البطن المتكررة (الشقيقة البطنية).
• القيء الدوري.

2. سن المراهقة وبداية العشرينيات
هذا هو العمر الأكثر شيوعاً لبداية نوبات الصداع النصفي التقليدية. تشير الدراسات إلى أن معظم الأشخاص المصابين تبدأ لديهم النوبات قبل سن الـ 40.

3. مرحلة النضج (بعد الـ 40)
من غير الشائع أن تبدأ الشقيقة لأول مرة بعد سن الخمسين. إذا بدأ صداع شديد وجديد في هذا العمر، فعادةً ما يطلب الأطباء فحوصات إضافية للتأكد من عدم وجود أسباب أخرى.
<hr>حقائق هامة حول توقيت النوبات:
• التأثير الهرموني: بالنسبة للإناث، غالباً ما تزداد وتيرة النوبات أو تبدأ مع التغيرات الهرمونية (سن البلوغ).
• الذروة: تصل الشقيقة عادةً إلى أقصى درجات حدتها وتكرارها في الثلاثينيات من العمر.
• التحسن مع العمر: يلاحظ الكثير من المصابين أن النوبات تصبح أقل حدة أو تختفي تماماً بعد سن الـ 50 أو بعد انقطاع الطمث لدى النساء.
<hr>ملاحظة: إذا كنت تسأل بسبب صداع مفاجئ وشديد بدأ مؤخراً، فمن المهم جداً استشارة الطبيب لتشخيص الحالة بدقة.

هل ترغب في معرفة المحفزات الشائعة التي قد تؤدي لبدء هذه النوبات؟</td>
</tr>
<tr>
<td>16</td>
<td>هل يمكن أن يصاب الأطفال بالشقيقة؟</td>
<td>نعم، يمكن للأطفال بالتأكيد أن يصابوا بالشقيقة (الصداع النصفي)، وهي أكثر شيوعاً مما قد يتخيله البعض. في الواقع، تختلف الشقيقة عند الأطفال في بعض الأحيان عن البالغين، مما يجعل تشخيصها يحتاج</td>
</tr>
</table>

Chatbot 2 (Google Gemini 3 Flash Mode)

إلى انتباه دقيق لسلوك الطفل.

إليك أهم ما يجب معرفته عن الشقيقة عند الأطفال:

1. كيف تختلف أعراضها عند الأطفال؟

بينما يشتكي الكبار عادة من ألم في جانب واحد من الرأس، فإن الأطفال غالباً ما يشعرون بالألم في **كامل الرأس أو الجبهة**. ومن الأعراض الشائعة لديهم:

- **ألم نابض:** يشبه النبض أو "الخفقان" في الرأس.
- **الغثيان والقيء:** وهي أعراض شائعة جداً عند الأطفال وقد تكون هي العرض الأساسي (تسمى أحياناً "الشقيقة البطنية").
- **الحساسية:** ينزعج الطفل بشدة من الأضواء الساطعة، الأصوات العالية، وحتى الروائح القوية.
- **تغير السلوك:** قد يتوقف الطفل فجأة عن اللعب، يصبح عصبياً، أو يرغب في النوم في غرفة مظلمة وهادئة.
- **شحوب الوجه:** غالباً ما يبدو الطفل شاحب اللون خلال النوبة.

2. مسببات الشقيقة عند الأطفال

هناك عوامل قد تحفز النوبة لدى الطفل، منها:

- **العوامل الوراثية:** إذا كان أحد الوالدين مصاباً بالشقيقة، فهناك احتمال كبير (يصل لـ 50% أو أكثر) أن يصاب الطفل بها.
- **قلة النوم:** عدم انتظام ساعات النوم أو السهر الزائد.
- **الجفاف:** عدم شرب كميات كافية من الماء.
- **الضغوط النفسية:** القلق من المدرسة، الامتحانات، أو المشاكل العائلية.
- **النظام الغذائي:** تخطي الوجبات، أو تناول أطعمة معينة (مثل الشوكولاتة، الأجبان القديمة، أو الأطعمة التي تحتوي على مادة MSG).

3. نصائح للتعامل مع النوبة في المنزل

1. **الراحة في بيئة هادئة:** بمجرد بدء الألم، اجعل طفلك يستلقي في غرفة مظلمة وباردة بعيداً عن الشاشات والضجيج.
2. **الكمادات الباردة:** وضع قطعة قماش باردة على جبهة الطفل قد يخفف الألم.
3. **النوم:** غالباً ما تنتهي نوبة الشقيقة عند الأطفال بعد نوم عميق.
4. **الأدوية:** يمكن استخدام مسكنات الألم المخصصة للأطفال (مثل الباراسيتامول أو الإيبوبروفين) بعد استشارة الطبيب لتحديد الجرعة المناسبة.

متى يجب زيارة الطبيب فوراً؟

يجب استشارة الطبيب إذا كان الصداع:

- مصحوباً بتصلب في الرقبة أو حمى.
- يحدث بعد إصابة في الرأس.

Chatbot 2 (Google Gemini 3 Flash Mode)

• يوقظ الطفل من النوم بسبب شدة الألم.<br>• يؤدي إلى فقدان التوازن أو ضعف في العضلات.<br><br>**هل تود مني مساعدتك في إعداد "مفكرة صداع" لمراقبة نمط الألم لدى طفلك ومشاركتها مع الطبيب؟**		
نعم، تُصنف **الشقيقة (الصداع النصفي)** طبياً على أنها **مرض عصبي مزمن**.<br><br>إليك توضيح لما يعنيه ذلك وكيفية التعامل معه:<br><br>**لماذا تعتبر مرضاً مزمنًا؟**<br><br>• **طبيعة الحالة:** الشقيقة ليست مجرد "صداع عابر"، بل هي خلل في الجهاز العصبي يؤدي إلى نوبات متكررة قد تستمر مدى الحياة لدى الكثيرين.<br>• **الاستعداد الوراثي:** تلعب الوراثة دوراً كبيراً، مما يجعل الشخص "مهيأً" للإصابة بالنوبات عند التعرض لمحفزات معينة.<br>• **غياب "العلاج النهائي":** لا يوجد حالياً دواء يقضي على الشقيقة تماماً بحيث لا تعود أبداً، ولكن توجد علاجات فعالة جداً للسيطرة عليها.<br><br>**الفرق بين الشقيقة "العارضة" و"المزمنة"**<br><br>يفرق الأطباء بين حالتين:<br><br>1. **الشقيقة العارضة (Episodic):** نوبات تحدث من وقت لآخر (أقل من 15 يوماً في الشهر).<br>2. **الشقيقة المزمنة (Chronic Migraine):** عندما يعاني الشخص من الصداع لمدة **15 يوماً أو أكثر في الشهر**، منها 8 أيام على الأقل تكون نوبات شقيقة صريحة، وذلك لمدة تزيد عن 3 أشهر.<br><br>**هل يعني هذا أن الألم لن يتوقف؟**<br><br>بالطبع لا. الهدف من الطب الحديث هو تحويل الشقيقة من حالة "معطلة للحياة" إلى حالة "مسيطر عليها" من خلال:<br><br>• **العلاجات الوقائية:** أدوية أو حقن (مثل البوتكس أو الأجسام المضادة) تُؤخذ بانتظام لتقليل عدد النوبات وشدتها.<br>• **العلاجات المسكنة:** أدوية تؤخذ فور بدء النوبة لإيقافها.<br>• **تغيير نمط الحياة:** تنظيم النوم، شرب الماء، وتجنب المحفزات (مثل بعض الأطعمة أو التوتر).<br><br>**ملاحظة مهمة:** الكثير من الأشخاص يلاحظون تحسناً كبيراً مع التقدم في السن أو من	هل الشقيقة مرض مزمن؟	17

Chatbot 2 (Google Gemini 3 Flash Mode)		
		خلال الالتزام بخطة علاجية مناسبة، فكونها "مزمنة" لا يعني أنها ستظل بنفس الشدة دائماً.<br><br>هل تعاني من نوبات متكررة حالياً؟ يمكنني مساعدتك في معرفة المحفزات الشائعة أو تزويدك بنصائح حول كيفية تتبع نوباتك لعرضها على الطبيب.
18	ما مضاعفات الشقيقة؟	تُعد الشقيقة (الصداع النصفي) أكثر من مجرد صداع شديد؛ فهي حالة عصبية يمكن أن تؤدي، في حال عدم علاجها أو السيطرة عليها، إلى مضاعفات جسدية ونفسية.<br><br>إليك أهم مضاعفات الشقيقة المحتملة:<br><br>**1. الحالة الشقيقية (Status Migrainosus)**<br>هي نوبة صداع حادة ومستمرة تستمر لـ **أكثر من 72 ساعة**. تكون الآلام فيها شديدة جداً ولا تستجيب للأدوية المعتادة، وغالباً ما تتطلب دخول المستشفى لتلقي السوائل والأدوية عبر الوريد بسبب القيء المستمر والجفاف.<br><br>**2. السكتة الدماغية الشقيقية (Migrainous Infarction)**<br>تحدث هذه المضاعفة النادرة عندما تترافق نوبة الشقيقة مع "أورة" (Aura) أو أعراض بصرية وحسية تستمر لفترة طويلة. في هذه الحالة، يتسبب نقص تدفق الدم إلى الدماغ أثناء النوبة في حدوث سكتة دماغية.<br>• **ملاحظة:** النساء اللواتي يعانين من الشقيقة مع "أورة" واللواتي يدخنّ أو يتناولن حبوب منع الحمل يكنّ أكثر عرضة لهذا الخطر.<br><br>**3. الصداع التحولي (الصداع الناتج عن الإفراط في استخدام الأدوية)**<br>عند استخدام مسكنات الألم (مثل الإيبوبروفين أو التريبتان) لأكثر من 10 إلى 15 يوماً في الشهر، قد يؤدي ذلك إلى حلقة مفرغة حيث يسبب الدواء نفسه صداعاً جديداً بمجرد انتهاء مفعوله، مما يحول الشقيقة من نوبات متقطعة إلى **صداع مزمن**.<br><br>**4. النوبات التشنجية (Migralepsy)**<br>في حالات نادرة، يمكن أن تؤدي نوبة الشقيقة (التي تسبقها أورة) إلى تحفيز نوبة صرع أو تشنجات مباشرة بعد النوبة أو أثنائها.<br><br>**5. مشاكل الصحة النفسية**<br>بسبب الألم المتكرر والتأثير على جودة الحياة، يزداد خطر الإصابة بـ:<br>• **الاكتئاب.**<br>• **القلق العام.**

<table>
<tr><th colspan="3">Chatbot 2 (Google Gemini 3 Flash Mode)</th></tr>
<tr><td></td><td></td><td>

- **الرهاب (خوف دائم من قدوم النوبة التالية).**

6. مضاعفات جسدية أخرى

- **مشاكل هضمية:** مثل الغثيان والقيء المزمن الذي قد يؤدي إلى الجفاف.
- **أعراض بصرية دائمة:** في حالات نادرة جداً، قد تترك النوبات المتكررة تأثيراً طفيفاً مستمراً على الرؤية (ما يعرف بـ "الثلج البصري").

نصيحة هامة: إذا لاحظت تغيراً في نمط الصداع، أو أصبح الصداع هو "الأسوأ في حياتك"، أو ترافق مع ضعف في أطرافك أو صعوبة في الكلام، يجب التوجه للطوارئ فوراً.

هل ترغب في معرفة المزيد عن كيفية الوقاية من تحول الشقيقة إلى صداع مزمن؟

</td></tr>
<tr><td>19</td><td>هل تتحسن الشقيقة مع التقدم بالعمر؟</td><td>

نعم، في أغلب الحالات، **تتحسن الشقيقة (الصداع النصفي) مع التقدم في العمر**. تشير الدراسات الطبية إلى أن وتيرة النوبات وشدتها تميل إلى الانخفاض تدريجياً، خاصة بعد سن الخمسين.

ومع ذلك، فإن هذا التحسن يختلف من شخص لآخر، وتلعب الهرمونات والعوامل الوراثية دوراً كبيراً في هذا المسار.

كيف تتغير الشقيقة عبر مراحل العمر؟

- **مرحلة الشباب (20 - 40 عاماً):** عادة ما تكون هذه الفترة هي "ذروة" المرض، حيث تكون النوبات في أقصى شدتها وتكرارها، وغالباً ما ترتبط بضغوط الحياة والنشاط البدني والهرمونات.
- **بعد سن الخمسين:** يبدأ عدد النوبات في التناقص لدى معظم الناس. تشير الإحصائيات إلى أن حوالي **40%** من المصابين يتوقف لديهم الصداع تماماً بحلول سن الـ 65.
- **لدى النساء (سن اليأس):** يعد انقطاع الطمث نقطة تحول كبرى؛ فبمجرد استقرار مستويات هرمون الإستروجين بعد انتهاء "مرحلة ما حول انقطاع الطمث"، تشعر الكثير من النساء بتحسن ملحوظ أو اختفاء تام للنوبات.

هل يمكن أن تتغير "طبيعة" النوبة؟

أحياناً لا تختفي الشقيقة تماماً، بل **تغير شكلها** عند كبار السن، وهو ما يعرف بـ "تطور الشقيقة":

1. **الشقيقة بلا صداع (:Aura Without Headache)** قد يعاني كبار السن من "الأورة" أو "الهالة" (مثل رؤية ومضات ضوئية أو خطوط زجزاجية) دون أن يتبعها ألم في الرأس.

</td></tr>
</table>

Chatbot 2 (Google Gemini 3 Flash Mode)

		2. **أعراض غير نمطية:** قد تزداد أعراض مثل الدوار أو اضطرابات التوازن بدلاً من الألم النابض التقليدي.
		### متى يكون الصداع في سن متأخرة مقلقاً؟<br>بينما تتحسن الشقيقة عادةً، فإن ظهور صداع **جديد** تماماً بعد سن الخمسين يتطلب استشارة طبية فورية، لأنه قد لا يكون شقيقة بل عرضاً لحالات أخرى (مثل ضغط الدم أو مشاكل الأوعية الدموية).
		**هل تسأل لأنك لاحظت تغيراً في نمط النوبات لديك مؤخراً، أم بخصوص أحد الوالدين؟** يمكنني مساعدتك في معرفة المحفزات الشائعة التي يجب تجنبها في هذه المرحلة.
20	ما علاج نوبات الشقيقة؟ و متى يجب أخذ العلاج؟	علاج نوبات الشقيقة (الصداع النصفي) ينقسم إلى قسمين أساسيين: **علاج دوائي** لإيقاف الألم عند بدء النوبة، و**إجراءات سلوكية** لتخفيف حدتها.<br>### أولاً: العلاج الدوائي للنوبات (العلاج الحاد)<br>تتفاوت الأدوية حسب شدة النوبة، وأهمها:<br>1. **مسكنات الألم البسيطة:** مثل الباراسيتامول، والأسبرين، والإيبوبروفين (Ibuprofen). تُستخدم للنوبات الخفيفة إلى المتوسطة.<br>2. **أدوية "التريبتان" (:Triptans)** مثل "سوماتريبتان". وهي أدوية متخصصة للشقيقة تعمل على تضييق الأوعية الدموية ومنع إشارات الألم في الدماغ. تُعد الأكثر فعالية للنوبات الشديدة.<br>3. **أدوية مضادة للغثيان:** تُؤخذ أحياناً مع المسكنات لأن الغثيان يرافق نوبات الشقيقة غالباً، كما أنها تساعد الجسم على امتصاص المسكنات بشكل أفضل.<br>4. **الفئات الحديثة (:Gepants & Ditans)** مثل "لاسميديتان" أو "ريميجيبانت"، وتُستخدم للأشخاص الذين لا يستجيبون للتريبتان أو يعانون من أمراض القلب.<br>### ثانياً: متى يجب أخذ العلاج؟<br>التوقيت هو العامل الأهم في نجاح العلاج:<br>• **القاعدة الذهبية:** يجب أخذ الدواء **فور الشعور ببدء النوبة** (عندما يكون الألم لا يزال خفيفاً). كلما تأخرت في أخذ الدواء، زادت صعوبة إيقاف الألم.<br>• **في حالة "الهالة" (:Aura)** إذا كنت تعاني من أعراض تسبق الصداع (مثل ومضات ضوء أو تنميل)، يُنصح عادةً بانتظار بدء الصداع الفعلي قبل أخذ أدوية "التريبتان"، بينما يمكن أخذ المسكنات العادية عند بدء الهالة.<br>• **تنبيه هام:** تجنب الإفراط في استخدام المسكنات (أكثر من يومين في الأسبوع)، لأن ذلك قد يؤدي إلى ما يسمى "صداع فرط استخدام الأدوية"، وهو صداع ارتدادي يزداد سوءاً مع الوقت.

Chatbot 2 (Google Gemini 3 Flash Mode)		
		**ثالثاً: نصائح تكميلية أثناء النوبة**<br><br>عند بدء النوبة، يمكن أن تساعدك هذه الخطوات في تقليل شدتها:<br>• **الراحة في غرفة مظلمة وهادئة:** لأن المصاب بالشقيقة غالباً ما يتحسس من الضوء والصوت.<br>• **الكمادات الباردة:** وضع قطعة قماش باردة أو كيس ثلج على الجبهة أو خلف الرقبة.<br>• **شرب الماء:** للتأكد من أن الجفاف ليس سبباً في تفاقم النوبة.<br><br>**ملاحظة:** إذا كانت النوبات تتكرر معك أكثر من 4 مرات في الشهر، فقد تحتاج لمناقشة "العلاجات الوقائية" مع طبيبك، وهي أدوية تؤخذ يومياً لتقليل تكرار النوبات وليس لعلاجها عند حدوثها.<br><br>**هل تود أن أساعدك في إعداد قائمة بالأسئلة التي يمكنك طرحها على طبيبك لتحديد نوع العلاج الأنسب لك؟**
21	ما الأدوية الوقائية لنوبات الشقيقة؟	تُستخدم الأدوية الوقائية للشقيقة (الصداع النصفي) بهدف تقليل عدد النوبات، وشدتها، ومدتها. عادةً ما يوصي الأطباء بهذه الأدوية إذا كانت النوبات تتكرر أكثر من مرتين أو ثلاث مرات في الشهر، أو إذا كانت النوبات شديدة لدرجة تعطل الحياة اليومية.<br><br>تنقسم هذه الأدوية إلى عدة فئات رئيسية، ويتم اختيار الأنسب منها بناءً على الحالة الصحية العامة للمريض:<br><br>**1. أدوية ضغط الدم (حاصرات بيتا وقنوات الكالسيوم)**<br>تعتبر من الخيارات الأولى والأكثر شيوعاً، وتعمل على تنظيم تدفق الدم ونبضات القلب.<br>• **أمثلة:** بروبرانولول (Inderal)، ميتوبرولول، وفيراباميل.<br>• **ملاحظة:** قد لا تناسب من يعانون من الربو أو انخفاض شديد في ضغط الدم.<br><br>**2. مضادات الاكتئاب**<br>تُستخدم بجرعات أقل من تلك المستخدمة لعلاج الاكتئاب، وتعمل على رفع مستوى السيروتونين وناقلات عصبية أخرى تؤثر على الألم.<br>• **أمثلة:** أميتريبتيلين (Amitriptyline) وفينلافاكسين.<br>• **الأعراض الشائعة:** قد تسبب النعاس أو جفاف الفم.<br><br>**3. مضادات التشنج (أدوية الصرع)**<br>تساعد في تهدئة الخلايا العصبية المفرطة في الدماغ والتي تحفز النوبة.

<table>
<tr><th colspan="3">Chatbot 2 (Google Gemini 3 Flash Mode)</th></tr>
<tr><td></td><td></td><td>

- **أمثلة:** توبيراميت (Topamax) وفالبورات الصوديوم (Depakote).
- **تنبيه:** يمنع استخدام "فالبورات" للنساء في سن الإنجاب إلا بضوابط صارمة جداً بسبب خطره على الأجنة.

4. العلاجات الحديثة (مضادات CGRP)

هي فئة متطورة تستهدف بروتيناً معيناً (CGRP) يرتفع مستواه في الدماغ أثناء نوبة الشقيقة.

- **حقن شهرية:** مثل إيرينوماب (Aimovig) وفريمانيزوماب (Ajovy).
- **أقراص فموية (:Gepants)** مثل أتوجيبانت (Qulipta) وريميجيبانت (Nurtec)، وتتميز بآثار جانبية أقل.

5. حقن البوتوكس (Botox)

تُستخدم خصيصاً لحالات **الشقيقة المزمنة** (التي تستمر لـ 15 يوماً أو أكثر في الشهر). تُحقن في عضلات الرأس والرقبة كل 12 أسبوعاً تقريباً.

نصائح عند بدء العلاج الوقائي:

- **الصبر:** قد يستغرق الدواء من 4 إلى 12 أسبوعاً لتبدأ نتائجه في الظهور بشكل واضح.
- **المفكرة:** يُفضل الاحتفاظ بـ "يوميات الصداع" لتسجيل عدد النوبات وشدتها لمساعدة طبيبك في تقييم فاعلية الدواء.
- **التدرج:** يبدأ الطبيب غالباً بجرعة منخفضة ويزيدها تدريجياً لتجنب الآثار الجانبية.

> **ملاحظة هامة:** يجب عدم تناول أي من هذه الأدوية إلا تحت إشراف طبيب متخصص، حيث أن لكل منها موانع استعمال وتفاعلات دوائية معينة.

هل تود مني مساعدتك في إعداد قائمة بالأسئلة التي يمكنك طرحها على طبيبك في زيارتك القادمة بخصوص هذه الأدوية؟

</td></tr>
<tr><td>22</td><td>هل يمكن علاج الشقيقة بطرق منزلية أو بديلة؟</td><td>

نعم، يمكن التعامل مع الشقيقة (الصداع النصفي) من خلال مجموعة واسعة من الطرق المنزلية والبديلة التي أثبتت فعاليتها في تخفيف حدة النوبات أو تقليل تكرارها. تنقسم هذه الطرق إلى إجراءات فورية أثناء النوبة، وتغييرات نمط حياة طويلة الأمد.

1. إجراءات فورية لتخفيف الألم (عند بدء النوبة)

- **البيئة المظلمة والهادئة:** بما أن الحساسية للضوء والصوت من أشهر أعراض الشقيقة، فإن الاسترخاء في غرفة مظلمة وهادئة يساعد كثيراً في تقليل التحفيز العصبي.
- **الكمادات الباردة أو الدافئة:** وضع كمادة باردة على الجبهة أو خلف الرقبة يمكن أن يكون له

</td></tr>
</table>

<table>
<tr><th colspan="3">Chatbot 2 (Google Gemini 3 Flash Mode)</th></tr>
<tr><td>
تأثير مخدر للألم، بينما تساعد الكمادات الدافئة في إرخاء العضلات المتوترة.
• **الزنجبيل:** أظهرت دراسات أن الزنجبيل قد يكون بفعالية بعض الأدوية المسكنة في تخفيف آلام الشقيقة، كما أنه ممتاز لتقليل الغثيان المصاحب لها.
• **الكافيين بكميات معتدلة:** شرب القليل من القهوة أو الشاي في بداية النوبة قد يساعد في تقليص الأوعية الدموية وتخفيف الألم، لكن احذر من الإفراط لأنه قد يسبب صداعاً ارتدادياً.

2. الطب البديل والمكملات الغذائية
• **المغنيسيوم:** نقص المغنيسيوم مرتبط بزيادة نوبات الشقيقة. تناول الأطعمة الغنية به (كالمكسرات والخضروات الورقية) أو المكملات (بعد استشارة الطبيب) قد يقلل من تكرار النوبات.
• **الزيوت العطرية:** تدليك الصدغين بزيت **النعناع** أو استنشاق زيت **اللافندر** (الخزامى) لمدة 15 دقيقة يساعد في تهدئة الجهاز العصبي وتقليل حدة الألم.
• **الوخز بالإبر (Acupuncture):** يعتبر من أكثر العلاجات البديلة المعترف بها طبياً للوقاية من الشقيقة.
• **عشبة "أقحوان زهرة الذهب" (Feverfew):** تستخدم تقليدياً كعلاج وقائي لتقليل عدد مرات حدوث الصداع.

3. تقنيات الاسترخاء ونمط الحياة
• **العلاج بالضغط (Acupressure):** الضغط على النقطة الموجودة بين الإبهام والسبابة (تسمى LI-4) لمدة دقيقتين قد يساعد في تخفيف آلام الرأس.
• **تنظيم النوم:** الالتزام بمواعيد نوم واستيقاظ ثابتة ضروري جداً، حيث إن اضطراب الساعة البيولوجية من أكبر محفزات الشقيقة.
• **اليوجا والتأمل:** تساعد هذه الممارسات في تقليل مستويات التوتر وتخفيف الشد العضلي في الرقبة والكتفين.
• **الترطيب المستمر:** الجفاف هو محفز خفي للصداع؛ لذا احرص على شرب كميات كافية من الماء طوال اليوم.
<hr>
نصيحة هامة: يُنصح دائماً بالاحتفاظ بـ **"مفكرة الصداع"** لتسجيل الأطعمة أو الظروف التي تسبق كل نوبة، مما يساعدك في تحديد المحفزات الخاصة بك وتجنبها.

هل تلاحظ وجود محفزات معينة تزيد من حدة الصداع لديك (مثل أطعمة معينة أو قلة النوم)، لكي أقترح عليك حلولاً أكثر تخصيصاً؟
</td><td></td><td></td></tr>
<tr><td>تُعد الشقيقة (الصداع النصفي) من التحديات الشائعة خلال الحمل، والخبر السار هو أن نوباتها تتحسن غالباً لدى الكثير من النساء بعد الثلث الأول. ومع ذلك، عند اختيار العلاج، يتم التركيز دائمًا على الموازنة بين تخفيف الألم وسلامة الجنين.</td><td>ما خيارات علاج الشقيقة أثناء الحمل؟</td><td>23</td></tr>
</table>

Chatbot 2 (Google Gemini 3 Flash Mode)

إليكِ الخيارات المتاحة والموصى بها وفقاً للإرشادات الطبية لعام 2026:

1. العلاجات غير الدوائية (الخط الأول)

يُفضل دائماً البدء بالحلول الطبيعية لتقليل الحاجة للأدوية:

- **تجنب المحفزات:** الحفاظ على جدول نوم منتظم، وتناول وجبات صغيرة متكررة لتجنب انخفاض سكر الدم، وشرب كميات كافية من الماء.
- **العلاج بالبرودة:** وضع كمادات باردة أو كيس ثلج على الجبهة أو خلف الرقبة عند بدء النوبة.
- **الراحة في بيئة هادئة:** الاسترخاء في غرفة مظلمة وهادئة بعيداً عن الأضواء والضوضاء.
- **تقنيات الاسترخاء:** مثل التنفس العميق، اليوغا المخصصة للحوامل، والارتجاع البيولوجي (Biofeedback).

2. العلاجات الدوائية للنوبات الحادة

إذا كانت الطرق الطبيعية غير كافية، يمكن اللجوء للأدوية **بعد استشارة الطبيب**:

الدواء	الحالة والأمان
**الباراسيتامول (أدول/باندول)**	الخيار الأول والأكثر أماناً طوال فترة الحمل (بأقل جرعة ولأق
**ميتوكلوبراميد ((Reglan**	يُستخدم لعلاج الغثيان المصاحب للشقيقة، ويُعتبر آمناً.
**السوماتريبتان ((Sumatriptan**	قد يصفه الطبيب في الحالات الشديدة إذا لم يستجب الجسم للبار
**مضادات الالتهاب (إيبوبروفين)**	قد تُستخدم بحذر في **الثلث الثاني فقط**، ويجب تجنبها تماماً في والثالث.

3. العلاجات الوقائية

إذا كانت النوبات متكررة وشديدة جداً، قد يقترح الطبيب أدوية وقائية مثل:

- **المغنيسيوم وفيتامين 2B (ريبوفلافين):** مكملات غذائية قد تساعد في تقليل التكرار.
- **حاصرات بيتا (مثل :(Propranolol** تُعتبر من الخيارات الوقائية الآمنة نسبياً تحت إشراف

Chatbot 2 (Google Gemini 3 Flash Mode)		
طبي دقيق.		
⚠ **أدوية يجب تجنبها تماماً:**<br>• **مشتقات الإرغوتامين (:(Ergotamine** لأنها قد تسبب انقباضات في الرحم وتؤثر على تروية الجنين.<br>• **الأدوية التي تحتوي على الأسبرين بجرعات عالية** (إلا إذا وصفه الطبيب بجرعة منخفضة لأسباب أخرى).<br><br>**ملاحظة هامة:** يجب التوجه للطبيب فوراً إذا كان الصداع مفاجئاً وشديداً جداً (كأنه "صدمة برق")، أو إذا ترافق مع تورم في الوجه واليدين أو زغللة في النظر، فقد يكون ذلك مؤشراً على حالة أخرى مثل ما قبل تسمم الحمل.<br><br>**هل تودين أن أساعدكِ في إعداد قائمة أسئلة لمناقشتها مع طبيبكِ في موعدكِ القادم؟**		
سؤالك مهم جداً ويشغل بال الكثيرين، فمرض الشقيقة (الصداع النصفي) ليس مجرد صداع عابر، بل هو حالة عصبية تؤثر على جودة الحياة بشكل كبير.<br><br>بصراحة وموضوعية علمية، **لا يوجد حالياً "علاج نهائي"** بمعنى اختفاء المرض تماماً وإلى الأبد بمجرد تناول دواء معين، لكن الخبر الجيد هو أن العلم قطع شوطاً طويلاً في **"السيطرة الكاملة"** عليه.<br><br>إليك التفاصيل التي توضح كيف يمكن التعايش مع الشقيقة لدرجة تشبه الشفاء:<br><br>**1. مفهوم "الشفاء" في الشقيقة**<br>بدلاً من البحث عن اختفاء المرض للأبد، يركز الأطباء على هدفين أساسيين يحققان راحة مشابهة للشفاء:<br>• **تقليل التكرار:** تحويل الحالة من نوبات أسبوعية إلى نوبات نادرة جداً (مرة كل بضعة أشهر أو سنة).<br>• **تقليل الشدة:** جعل النوبة "خفيفة" جداً بحيث لا تعيقك عن ممارسة حياتك اليومية.<br><br>**2. العوامل التي تؤدي إلى اختفاء الشقيقة تلقائياً**<br>في كثير من الحالات، تختفي الشقيقة أو تتحسن بشكل جذري نتيجة تغيرات بيولوجية:<br>• **العمر:** غالباً ما تتحسن الحالة بشكل كبير بعد سن الخمسين أو عند انقطاع الطمث لدى النساء، نظراً لارتباطها بالهرمونات.<br>• **تغيير نمط الحياة:** اكتشاف "المحفزات" وتجنبها (مثل أنواع معينة من الطعام، قلة النوم، أو	هل يمكن الشفاء من الشقيقة بشكل نهائي؟	24

#	السؤال	Chatbot 2 (Google Gemini 3 Flash Mode)
		التوتر) قد ينهي النوبات تماماً لدى البعض.<br><br>**3. العلاجات الحديثة (ثورة في السيطرة)**<br><br>هناك جيل جديد من الأدوية جعل السيطرة على الشقيقة أمراً ممكناً جداً:<br>• **الأجسام المضادة (:(CGRP** وهي حقن تؤخذ شهرياً أو كل بضع أشهر، وقد أثبتت فعالية مذهلة في منع حدوث النوبات قبل بدئها.<br>• **إبر البوتوكس:** معتمدة طبياً لعلاج الشقيقة المزمنة وتساعد في تقليل عدد أيام الصداع بشكل كبير.<br>• **الأجهزة المحفزة للأعصاب:** أجهزة إلكترونية صغيرة تستخدم لتنظيم الإشارات العصبية وتقليل الألم.<br><br>**4. نصيحة للمستقبل**<br><br>بما أن الشقيقة مرتبطة بجهازك العصبي وحساسيته للمؤثرات، فإن أفضل "شفاء" متاح حالياً هو **الإدارة الذكية**:<br>1. **مذكرات الصداع:** لتحديد المحفزات بدقة.<br>2. **العلاج الوقائي:** إذا كانت النوبات تتكرر أكثر من 4 مرات شهرياً.<br>3. **الرياضة والنوم المنتظم:** يرفعان من عتبة تحمل الدماغ للألم.<br><br>**هل ترغب في أن أساعدك في إعداد قائمة بالأسئلة التي يمكنك طرحها على طبيب الأعصاب في زيارتك القادمة لضمان الحصول على أحدث خطة علاجية؟**
25	كيف تؤثر الشقيقة على حياة المصابين؟	تُعد **الشقيقة (الصداع النصفي)** أكثر من مجرد "صداع شديد"؛ فهي حالة عصبية معقدة تؤثر بشكل عميق على كافة جوانب حياة الشخص المصاب. لا يقتصر الأثر على وقت النوبة فحسب، بل يمتد ليشمل الجوانب النفسية، المهنية، والاجتماعية.<br><br>إليك تفصيل لكيفية تأثير الشقيقة على حياة المصابين:<br><br>**1. التأثير على الأداء المهني والدراسي**<br><br>تعتبر الشقيقة من الأسباب الرئيسية للإعاقة المؤقتة في بيئة العمل، وتتجلى آثارها في:<br>• **الغياب المتكرر:** اضطرار المصاب لأخذ إجازات مفاجئة بسبب شدة الألم وعدم القدرة على مواجهة الضوء أو الضجيج.<br>• **"الحضور الذهني الغائب":** قد يحضر المصاب للعمل لكن بإنتاجية منخفضة جدًا بسبب ضعف التركيز، وتشتت الانتباه، وبطء التفكير الناتج عن الألم أو الأدوية.<br>• **الخوف من فقدان الوظيفة:** القلق الدائم من نظرة الزملاء أو الإدارة بسبب الغيابات المتكررة

Chatbot 2 (Google Gemini 3 Flash Mode)

وغير المتوقعة.

2. الحياة الاجتماعية والعائلية

تؤدي الشقيقة غالباً إلى عزل المصاب عن محيطه:

- **إلغاء المخططات:** يضطر المصابون لإلغاء المناسبات الاجتماعية في اللحظات الأخيرة، مما قد يُفسر خطأً كبرود اجتماعي.
- **الضغط على العلاقات:** قد يشعر الشريك أو أفراد الأسرة بالإحباط أو العبء نتيجة تولي مسؤوليات المصاب أثناء نوباته.
- **الشعور بالذنب:** يعاني الكثيرون من ذنب عدم القدرة على اللعب مع أطفالهم أو المشاركة في اللحظات العائلية الهامة.

3. الصحة النفسية والعاطفية

هناك ارتباط وثيق بين الشقيقة والحالة النفسية:

- **قلق الترقب:** يعيش المصاب في حالة خوف دائم من "متى ستأتي النوبة القادمة؟"، مما يمنعه من الاستمتاع بلحظات الراحة.
- **الاكتئاب والقلق:** المصابون بالشقيقة المزمنة أكثر عرضة للإصابة بالاكتئاب بنسبة تصل إلى الضعف مقارنة بغيرهم.
- **الشعور بعدم الفهم:** لأن الشقيقة مرض "غير مرئي"، يعاني المصابون من شعور بأن الآخرين لا يقدرون حجم معاناتهم الحقيقية.

4. النمط الحياتي والقيود اليومية

تفرض الشقيقة قيوداً صارمة على نمط الحياة لتجنب "المحفزات":

- **الحرمان من الأطعمة:** تجنب أنواع معينة من الطعام أو الشراب قد يحبها المصاب.
- **الحساسية الحسية:** الاضطرار للعيش في غرف مظلمة وهادئة لساعات أو أيام.
- **اضطراب النوم:** تؤثر الشقيقة على جودة النوم، وقلة النوم بدورها تحفز النوبات، مما يخلق حلقة مفرغة.

ملخص التأثيرات

الجانب	نوع التأثير
**الجسدي**	ألم نابض، غثيان، حساسية للضوء، إرهاق شديد بعد النوبة.

Chatbot 2 (Google Gemini 3 Flash Mode)

**المادي**	تكاليف الأدوية، زيارات الأطباء، وفقدان الدخل بسبب التغيب.
**النفسي**	توتر مستمر، شعور بالعجز، وانخفاض تقدير الذات.

هل تعاني أنت أو شخص قريب منك من أعراض معينة وتود معرفة كيفية التعامل مع "المحفزات" البيئية لتقليل وتيرة النوبات؟

Chatbot 3 (Grok 4.1)

SN	السؤال	الرد
1	ما هي الشقيقة؟	الشقيقة (أو الصداع النصفي، المعروف علميًا باسم Migraine) هي اضطراب عصبي مزمن شائع يسبب نوبات متكررة من الصداع الشديد، غالبًا ما يكون نابضًا ويؤثر في جانب واحد من الرأس (قد ينتقل إلى الجانبين أحيانًا). تصيب النساء أكثر من الرجال بثلاث مرات تقريبًا، وتبدأ عادةً في سن المراهقة أو الشباب، وقد تقل شدتها بعد سن الـ50.<br><br>**الأعراض الرئيسية:**<br><br>• صداع شديد نابض، يزداد سوءًا مع الحركة أو النشاط البدني.<br>• غثيان، وقد يصل إلى التقيؤ.<br>• حساسية مفرطة للضوء (الضوء الساطع)، الصوت، أو الروائح.<br>• في بعض الحالات (حوالي 15-30%)، تسبق النوبة "هالة" (أورة) تشمل اضطرابات بصرية مثل رؤية ومضات ضوء، خطوط متعرجة، أو بقع عمياء مؤقتة، أو وخز في الوجه أو الأطراف.<br><br>تستمر النوبة عادةً من 4 إلى 72 ساعة إذا لم تُعالج، وقد تعيق الأنشطة اليومية تمامًا.

Chatbot 3 (Grok 4.1)

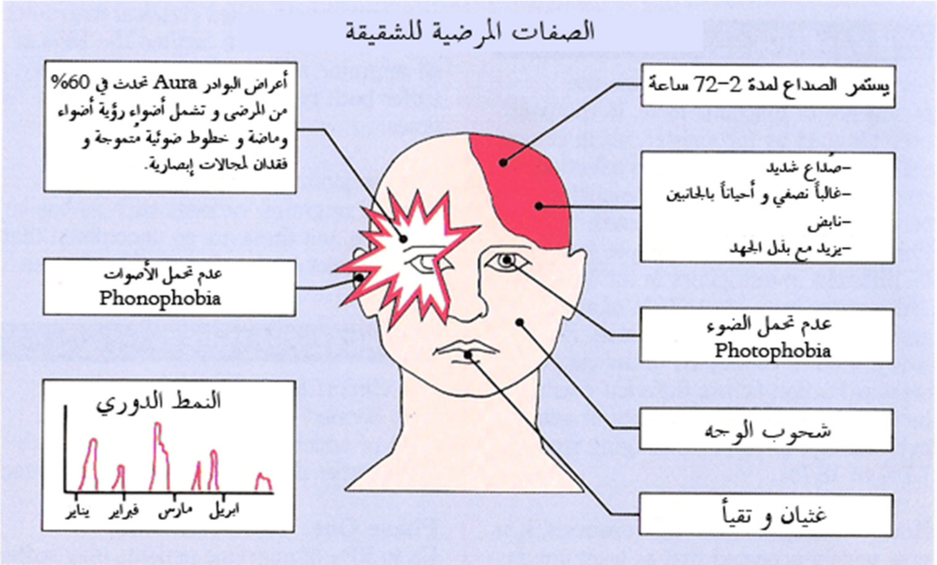

your-doctor.net

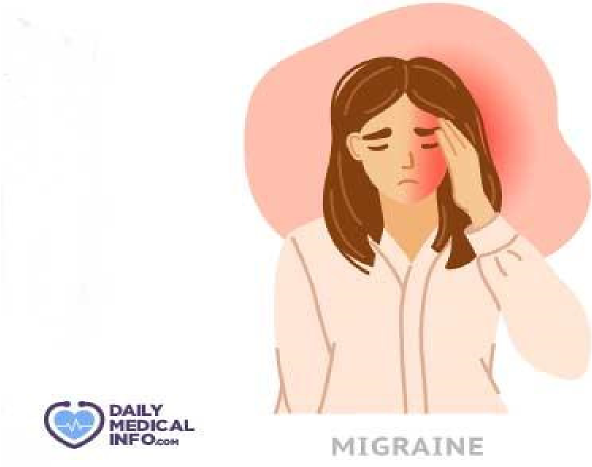

dailymedicalinfo.com

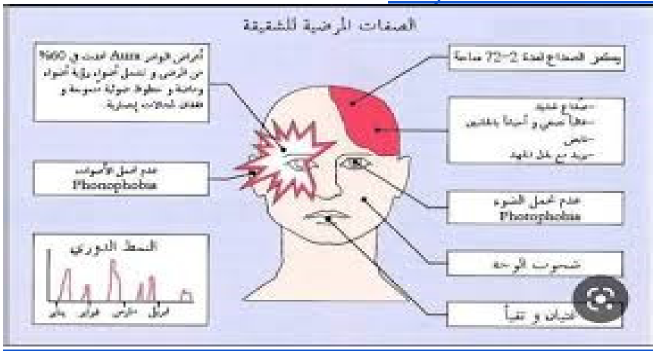

facebook.com

<table>
<tr><th colspan="3">Chatbot 3 (Grok 4.1)</th></tr>
<tr><td>

الأسباب والمحفزات:

السبب الدقيق غير معروف تمامًا، لكنه يتعلق بتغيرات في النشاط الكهربائي للدماغ، واضطرابات في الأوعية الدموية والمواد الكيميائية مثل السيروتونين. هناك عامل وراثي قوي (تسري في العائلات).

محفزات شائعة تشمل:

- الإجهاد النفسي أو الجسدي.
- تغيرات هرمونية (خاصة عند النساء أثناء الدورة الشهرية).
- بعض الأطعمة (مثل الشوكولاتة، الجبن المعتق، الكحول، المضافات الغذائية).
- قلة النوم أو الإفراط فيه، الجوع، أو الإفراط في الكافيين.
- أضواء ساطعة أو أصوات عالية.

العلاج والوقاية:

لا يوجد علاج شافٍ، لكن يمكن السيطرة عليه:

- مسكنات الألم البسيطة (مثل الإيبوبروفين) للنوبات الخفيفة.
- أدوية متخصصة مثل التريبتانات للنوبات الشديدة.
- أدوية وقائية يومية إذا كانت النوبات متكررة (مثل حاصرات بيتا أو مضادات الاكتئاب).
- تغييرات في نمط الحياة: نوم منتظم، تجنب المحفزات، ممارسة الرياضة، وإدارة التوتر.

إذا كنت تعاني من أعراض مشابهة، استشر طبيبًا عصبيًا للتشخيص الدقيق، حيث قد يحتاج الأمر إلى فحوصات لاستبعاد أسباب أخرى.

</td><td></td><td></td></tr>
<tr><td>

الشقيقة (الصداع النصفي) هي اضطراب عصبي شائع يأتي بأشكال متعددة، وتختلف أنواعها بناءً على الأعراض والأسباب. وفقاً لمصادر طبية موثوقة، هناك ثمانية أنواع رئيسية من الشقيقة، مع وصف مختصر لكل منها:

1. **الشقيقة بدون أورة**: النوع الأكثر شيوعاً، يشمل صداعاً نابضاً في جانب واحد من الرأس، متوسط إلى شديد، يستمر ساعات إلى أيام، ويزداد سوءاً بسبب الأضواء الساطعة أو الأصوات العالية أو الروائح القوية.
2. **الشقيقة مع أورة**: تحدث في حوالي ثلث الحالات، مع تجارب حسية مثل تغيرات بصرية (مثل أضواء وميضية)، أو إحساس بالدبابيس والإبر، أو صعوبة في الكلام، تبدأ تدريجياً وتتلاشى، غالباً قبل أو أثناء الصداع.
3. **الشقيقة مع أورة جذع الدماغ**: تُعرف أيضاً بالشقيقة الدهليزية أو البازيلارية، تسبب دوخة، طنين في الأذنين، رؤية مزدوجة، أو فقدان التوازن، تتطور تدريجياً وتتلاشى خلال دقائق.
4. **الشقيقة الشبكية**: أورة بصرية تؤثر على عين واحدة فقط، مثل بقع عمياء أو فقدان الرؤية، تنتشر وتتلاشى خلال دقائق؛ يتطلب تقييماً طبياً لاستبعاد حالات أخرى.
5. **الشقيقة الشللية**: تشبه الشقيقة مع أورة لكنها تشمل ضعفاً عضلياً مؤقتاً في جانب واحد من الجسم، من خفيف إلى شديد، يزول خلال 72 ساعة؛ قد تكون وراثية.
6. **الشقيقة الطمثية**: تحدث وفقاً لدورة الطمث، غالباً قبل أو بعد الحيض، يمكن أن تكون شديدة، وتشمل أي نوع من الشقيقة، تعالج وقائياً بأدوية مثل التريبتان أو وسائل منع الحمل المستمرة.

</td><td>ما أنواع الشقيقة؟</td><td>2</td></tr>
</table>

Chatbot 3 (Grok 4.1)

7. **الشقيقة المزمنة والشقيقة المستمرة**: المزمنة تشمل 15 نوبة أو أكثر شهرياً لشهور؛ المستمرة هي نوبة واحدة تستمر أكثر من 72 ساعة مع أعراض منهكة.<br>8. **الشقيقة البطنية**: غالباً عند الأطفال، تشمل ألماً حلقياً في البطن حول السرة، غثياناً، قيئاً، شحوباً، وفقدان الشهية؛ قد لا تشمل صداعاً، وغالباً تزول مع التقدم في العمر.<br><br>ملاحظة: إذا كنت تعاني من أعراض الشقيقة، استشر طبيباً للتشخيص الدقيق والعلاج المناسب.		
لا، **لا يوجد فرق جوهري** بين "الشقيقة" و"الصداع النصفي"؛ إنهما اسمان مختلفان لنفس الحالة الطبية، والتي تُعرف عالميًا باسم الـ **.Migraine**<br><br>**التوضيح:**<br><br>• **الصداع النصفي** (أو الشقيقة) هو نوع محدد من الصداع الشديد والمتكرر، يتميز عادةً بألم نابض في جانب واحد من الرأس (ليس دائمًا)، مصحوب بأعراض مثل الغثيان، القيء، حساسية مفرطة للضوء والصوت، وقد يسبقها "هالة" (أورة) مثل اضطرابات بصرية.<br>• هذا يختلف عن **الصداع العادي** (مثل صداع التوتر أو الصداع العارض)، الذي يكون أقل شدة، لا يصحبه غثيان عادةً، ولا يعيق الأنشطة اليومية بنفس الدرجة.<br><br>**لماذا يُستخدمان معًا؟**<br><br>• في اللغة العربية الطبية واليومية، يُستخدم "الصداع النصفي" للإشارة إلى الجانبية (نصف الرأس)، بينما "الشقيقة" مصطلح تاريخي أقدم يصف الشعور بـ"شق الرأس" من شدة الألم.<br>• معظم المصادر الطبية الموثوقة (مثل موقع الطبي، ويب طب، ويكيبيديا، ووزارة الصحة السعودية) تُعاملها كمرادفات تمامًا، مثل: "الصداع النصفي أو الشقيقة".<br><br>إذا كنت تعاني من صداع متكرر، يُفضل استشارة طبيب للتشخيص الدقيق، حيث قد يكون هناك أنواع فرعية (مع هالة أو بدونها) تتطلب علاجًا محددًا.	هل هناك فرق بين الشقيقة والصداع النصفي؟	3
**الفرق بين الشقيقة (الصداع النصفي) وصداع الجيوب الأنفية**<br><br>الشقيقة والصداع الناتج عن التهاب الجيوب الأنفية يشتركان في بعض الأعراض مثل الألم في الوجه أو الجبهة، والاحتقان الأنفي، مما يسبب خلطًا شائعًا بينهما. ومع ذلك، دراسات طبية موثوقة (مثل تلك من مؤسسة الصداع النصفي الأمريكية ومايو كلينك) تشير إلى أن **حوالي 90% من الحالات التي يُعتقد أنها صداع جيوب أنفية هي في الواقع شقيقة**، بينما صداع الجيوب الأنفية الحقيقي نادر نسبيًا ويرتبط دائمًا بالتهاب أو عدوى في الجيوب.<br><br>**جدول مقارنة الفرق الرئيسي**<br><br>**الجانب** | **الشقيقة ((Migraine** | **صداع الجيوب الأنفية (Sinus Headache)**	ما الفرق بين الشقيقة وصداع الجيوب الأنفية؟	4

Chatbot 3 (Grok 4.1)

**السبب**	اضطراب عصبي في الدماغ، يتعلق بالأعصاب والأوعية الدموية (وراثي غالبًا، يُحفز بالتوتر أو الطعام أو التغيرات الجوية).	التهاب أو عدوى في الجيوب الأنفية (فيروسية أو بكتيرية، أو حساسية).
**مكان الألم**	غالبًا جانب واحد من الرأس (قد ينتقل)، نبضي أو خافق، يمتد إلى الصدغ أو مؤخرة الرأس.	ضغط مستمر في الوجه: الجبهة، الخدود، حول العينين أو جسر الأنف (غالباً الجانبين).
**شدة الألم**	متوسط إلى شديد، يزداد مع الحركة أو النشاط.	ثابت وضاغط، يزداد عند الانحناء أو الاستلقاء.
**الأعراض المصاحبة**	- غثيان وقيء<br>- حساسية شديدة للضوء والصوت (وربما الروائح)<br>- اضطرابات بصرية (هالات أو وميض)<br>- إفرازات أنفية **شفافة ومائية**	- إفرازات أنفية **سميكة ملونة** (أصفر أو أخضر)<br>- حمى أحيانًا<br>- ضعف في حاسة الشم<br>- لا غثيان أو حساسية للضوء عادةً
**المدة**	4-72 ساعة عادةً، متكررة.	يستمر حتى يُعالج الالتهاب (أيام إلى أسابيع).
**العلاج**	أدوية خاصة بالشقيقة (تريبتانات)، مسكنات، راحة في غرفة مظلمة هادئة.	مضادات احتقان، مضادات حيوية إذا بكتيري، غسول أنفي، علاج الحساسية.

نصائح هامة

- إذا كان الصداع متكررًا بدون علامات عدوى واضحة (مثل حمى أو إفرازات ملونة)، فمن المحتمل أنه شقيقة.
- صداع الجيوب الأنفية الحقيقي يتحسن عادةً بعد علاج الالتهاب، بينما الشقيقة تحتاج علاجًا متخصصًا.
- **استشر طبيبًا** (أخصائي أعصاب أو أنف وأذن وحنجرة) للتشخيص الدقيق، خاصة إذا استمر الصداع أو صاحبه أعراض شديدة، فقد يحتاج الأمر إلى فحوصات مثل التصوير.

هذا التمييز يساعد في الحصول على العلاج المناسب وتجنب التشخيص الخاطئ الشائع.

Chatbot 3 (Grok 4.1)		
لا، صداع الشقيقة (الصداع النصفي أو الميغرين) **ليس خطيراً بحد ذاته** ولا يسبب الوفاة عادةً، بل هو اضطراب عصبي شائع يصيب أكثر من مليار شخص حول العالم سنوياً، ويُصنف كثاني أكبر سبب للإعاقة العالمية بعد السكتة الدماغية حسب منظمة الصحة العالمية (WHO). ومع ذلك، **يمكن أن يكون شديداً ومعيقاً جداً**، حيث يسبب ألماً نابضاً في جانب واحد من الرأس (أو الجانبين)، غثياناً، قيئاً، وحساسية للضوء والصوت والروائح، وقد يستمر من 4 إلى 72 ساعة أو أكثر إذا لم يُعالج.<br><br>**لماذا يُعتبر غير خطير عادةً؟**<br><br>• **ليست نوبات الشقيقة خطيرة**، بل تسبب إزعاجاً شديداً يمنع النشاط اليومي، كما يوضح دليل MSD الطبي وMayo Clinic. معظم الحالات تتحسن بالراحة في غرفة مظلمة وهادئة أو أدوية بسيطة مثل الإيبوبروفين أو الباراسيتامول.<br>• **لا تسبب ضرراً دائماً في الدماغ** في معظم الحالات، رغم وجود دراسات تشير إلى ظهور بقع بيضاء صغيرة (white matter lesions) في الدماغ لدى بعض المصابين المزمنين، لكنها غير مؤذية عادةً ولا تؤثر على الوظائف الإدراكية.<br><br>**المضاعفات النادرة أو المحتملة (غير شائعة لكن تستدعي الانتباه):**<br><br>رغم عدم الخطورة الأساسية، قد تحدث مشكلات إذا تُركت دون علاج أو في حالات خاصة:<br><br>1. **الحالة الشقيقية المستمرة ((Status Migrainosus**: نوبة شديدة تستمر أكثر من 72 ساعة، تسبب جفافاً وإرهاقاً شديداً، وقد تحتاج تدخلاً طبياً في المستشفى.<br>2. **السكتة الشقيقية ((Migrainous Infarction**: نادر جداً (خاصة عند النساء الشابات مع هالة بصرية)، حيث تضيق الأوعية الدموية وتقطع الأكسجين عن الدماغ، مما يشبه السكتة الدماغية.<br>3. **زيادة خطر السكتة الدماغية والقلب**: خاصة مع الشقيقة المصحوبة بهالة (أضواء ومضة أو تشوش بصري)، حيث يرتفع الخطر بنسبة تصل إلى 50% حسب American Migraine Foundation، لكن هذا نادر ومرتبط بعوامل أخرى مثل التدخين أو الحبوب المانعة للحمل.<br>4. **الصداع المرتد**: من الإفراط في مسكنات الألم (أكثر من 10-15 يوماً شهرياً)، مما يزيد التكرار.<br>5. **الشقيقة المزمنة**: أكثر من 15 يوماً شهرياً لـ3 أشهر، تؤدي إلى اكتئاب، قلق، وفقدان إنتاجية.<br><br>**علامات التحذير (استشر الطبيب فوراً إذا ظهرت):**<br><br>• صداع مفاجئ شديد "كالرعد" (thunderclap headache).<br>• أعراض تشبه السكتة: شلل في جانب، صعوبة كلام، فقدان رؤية دائم، تيبس رقبة، حمى، أو تغيرات في الوعي.<br>• نوبات جديدة بعد سن 50، أو مع تاريخ سرطان/ضعف مناعي.<br>• هالة (أورة) تستمر أكثر من ساعة.<br><br>**نصائح للوقاية والعلاج:**<br><br>• **تجنب المحفزات**: التوتر، نقص نوم، جوع، كافيين زائد، أضواء ساطعة، شوكولاتة، جبن قديم.<br>• **علاج منزلي**: راحة، كمادات باردة، شرب سوائل، مسكنات فورية.<br>• **علاج طبي**: مسكنات، تريبتانات (مثل سوماتريبتان)، وقائيات مثل أميتريبتيلين أو بروبرانولول إذا تكررت (أكثر من 4 نوبات شهرياً).<br>• **استشر طبيب أعصاب** للتشخيص الدقيق (لا يوجد اختبار واحد، يعتمد على الأعراض والتاريخ	هل صداع الشقيقة خطير؟	5

<table>
<tr><th colspan="3">Chatbot 3 (Grok 4.1)</th></tr>
<tr><td>العائلي).

ملاحظة هامة: هذه معلومات عامة، ليست بديلاً عن استشارة طبية. إذا كنت تعاني، اذهب إلى طبيب لتقييم حالتك الشخصية.</td><td></td><td></td></tr>
<tr><td>أعراض الشقيقة (الصداع النصفي)

الشقيقة هي حالة عصبية شائعة تسبب صداعًا شديدًا نابضًا، غالبًا في جانب واحد من الرأس، وتترافق مع أعراض أخرى مثل الغثيان، القيء، والحساسية الشديدة للضوء (الضوئية) والصوت (الصوتية). قد تستمر النوبة من 4 إلى 72 ساعة إذا لم تعالج، وتختلف الأعراض من شخص لآخر ومن نوبة لأخرى.

المراحل التي تمر بها نوبة الشقيقة

تمر نوبة الشقيقة عادةً بأربع مراحل رئيسية (وفقًا لمصادر موثوقة مثل Mayo Clinic ومنظمة الصحة العالمية)، لكن ليس كل المصابين يمرون بجميعها:

1. مرحلة البادرة أو التمهيدية (Prodrome) تحدث قبل الصداع بيوم أو يومين، وتكون إشارات تحذيرية مبكرة. تشمل الأعراض الشائعة:
o تغيرات في المزاج (اكتئاب، تهيج، أو فرط نشاط).
o رغبة شديدة في تناول أطعمة معينة.
o تعب عام أو إعياء.
o تصلب في الرقبة.
o كثرة التثاؤب أو العطش أو التبول. هذه المرحلة تحدث لدى معظم المصابين (حوالي 60-80%).
2. مرحلة الهالة أو الأورة (Aura) تحدث لدى حوالي ثلث المصابين فقط، قبل أو أثناء الصداع، وتستمر 20-60 دقيقة. تكون أعراض عصبية مؤقتة وقابلة للعكس، مثل:
o اضطرابات بصرية (ومضات ضوء، خطوط متعرجة، بقع عمياء، أو فقدان رؤية مؤقت).
o تنميل أو وخز في الوجه أو الأطراف.
o صعوبة في الكلام أو التفكير. (قد تحدث الشقيقة بدون هذه المرحلة، وهي الأكثر شيوعًا).
3. مرحلة الصداع أو النوبة الرئيسية (Headache Attack) المرحلة الرئيسية والأكثر ألمًا، تستمر 4-72 ساعة. تشمل:
o ألم نابض أو خافق في جانب واحد (أو كلا الجانبين) من الرأس، يزداد مع الحركة أو النشاط.
o غثيان وقيء.
o حساسية شديدة للضوء، الصوت، أو الروائح.
o دوار أو إرهاق.
4. مرحلة ما بعد النوبة (Postdrome) تحدث بعد انتهاء الصداع، وتستمر ساعات إلى يومين (تُعرف بـ"صداع الكحول" أو "الإرهاق بعد الشقيقة"). تشمل:
o إعياء شديد وتعب.
o صعوبة في التركيز.
o تغيرات مزاجية أو ألم عضلي خفيف.</td><td>ما هي أعراض الشقيقة؟ وما هي المراحل التي قد تمر بها النوبة؟</td><td>6</td></tr>
</table>

Chatbot 3 (Grok 4.1)

		**ملاحظة مهمة:** إذا كانت الأعراض جديدة أو شديدة (مثل فقدان رؤية مفاجئ، ضعف عضلي، أو صعوبة كلام)، استشر طبيبًا فورًا لاستبعاد حالات أخرى خطيرة. الشقيقة مرض مزمن، لكن يمكن السيطرة عليها بالعلاج والتجنب المحفزات (مثل التوتر، قلة النوم، أو بعض الأطعمة).
7	ما هي الهالة (الأورة) في الشقيقة؟	الهالة (أو الأورة، كما تُكتب أحيانًا Aura) في الشقيقة (الصداع النصفي أو Migraine) هي مجموعة من الأعراض العصبية المؤقتة التي تحدث عادةً قبل بدء الصداع الشديد، أو أحيانًا أثناءه، وتعمل كإشارة تحذيرية للنوبة. تحدث الهالة في حوالي 25-30% من حالات الشقيقة، وتُسمى الشقيقة المصحوبة بهالة "الشقيقة مع هالة" (Migraine with Aura)، بينما النوع الأكثر شيوعًا هو بدون هالة.<br><br>**أعراض الهالة الشائعة:**<br><br>• **البصرية** (الأكثر شيوعًا، تصيب حوالي 90% من الحالات): رؤية أضواء وامضة، خطوط متعرجة (zigzag lines)، نقاط لامعة، بقع عمياء مؤقتة (scintillating scotoma)، أو تشويش في الرؤية. تبدأ غالبًا في مركز الرؤية وتنتشر تدريجيًا.<br><br>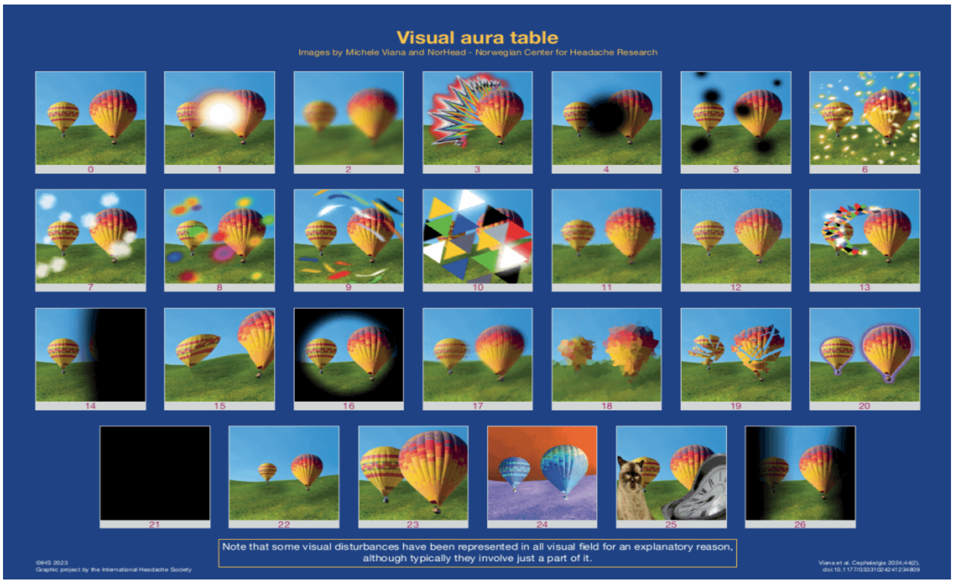<br><br>ihs-headache.org<br><br>en.wikipedia.org

Chatbot 3 (Grok 4.1)

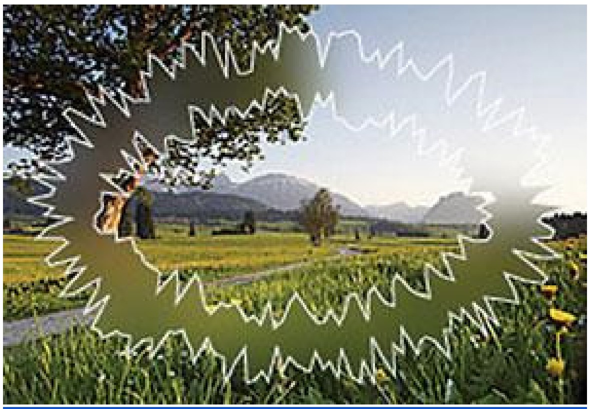

reviewofoptometry.com

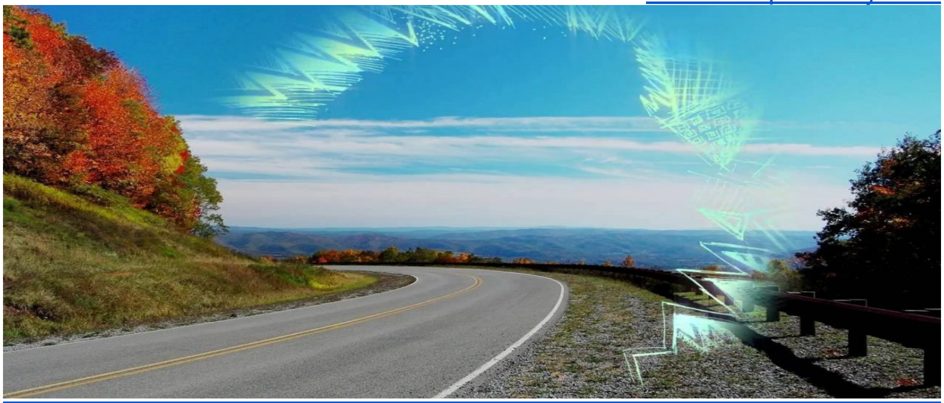

henryehooper.blog

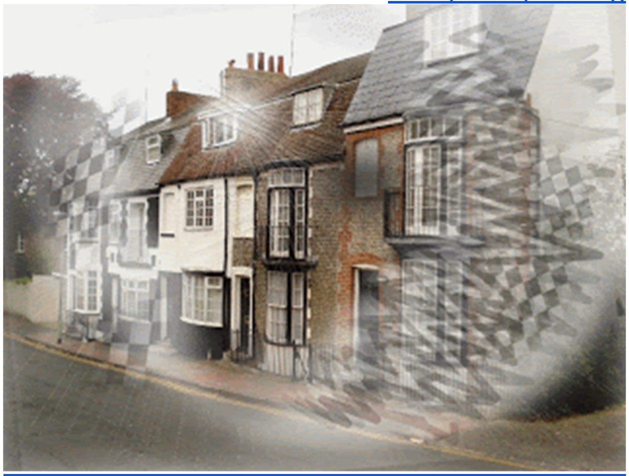

tonibrayermd.com

- **الحسية**: تنميل أو وخز (مثل دبابيس وإبر) في الوجه أو اليدين أو الأطراف، ينتشر تدريجيًا.
- **الكلامية**: صعوبة في الكلام أو العثور على الكلمات.
- **أخرى أقل شيوعًا**: دوار، ضعف عضلي، أو اضطرابات سمعية.

Chatbot 3 (Grok 4.1)		
		تتطور الأعراض تدريجيًا خلال 5-20 دقيقة، وتستمر عادةً من 5 إلى 60 دقيقة، ثم تختفي تمامًا. في بعض الحالات النادرة، تحدث الهالة بدون صداع لاحق (تُسمى "الشقيقة الصامتة").<br><br>**سبب الهالة:**<br><br>ترجع إلى ظاهرة عصبية تُسمى "الاكتئاب المنتشر القشري" (Cortical Spreading Depression)، وهي موجة كهربائية تنتشر في القشرة الدماغية، مما يسبب هذه الاضطرابات المؤقتة.<br><br>إذا كنت تعاني من هذه الأعراض لأول مرة، استشر طبيبًا لاستبعاد أسباب أخرى خطيرة مثل السكتة الدماغية، خاصة إذا كانت الأعراض مفاجئة أو طويلة الأمد. العلاج مشابه للشقيقة العادية، لكن الهالة تساعد في التشخيص المبكر.
8	هل القيء والغثيان من أعراض الشقيقة؟	نعم، **القيء والغثيان** من الأعراض الشائعة جدًا لمرض الشقيقة (المعروف أيضًا بالصداع النصفي أو Migraine).<br><br>يُصيب الغثيان حوالي 60-95% من المصابين بالشقيقة، بينما يحدث القيء في 30-60% من الحالات تقريبًا، وقد يكونان أكثر إزعاجًا من الصداع نفسه في بعض الأحيان. يمكن أن يظهر الغثيان قبل الصداع أو أثناءه أو بعده، وقد يؤدي إلى صعوبة في تناول الأدوية الفموية بسبب تأخر إفراغ المعدة (gastroparesis) المرتبط بالشقيقة.<br><br>**مصادر موثوقة تؤكد ذلك:**<br><br>• **مايو كلينك (Mayo Clinic)**: يُعد الغثيان والقيء من الأعراض الرئيسية للشقيقة، مصحوبًا بحساسية للضوء والصوت.<br>• **المعهد الوطني للاضطرابات العصبية والسكتة الدماغية (NINDS)**: يشمل أعراض الشقيقة الغثيان والقيء إلى جانب الصداع النابض.<br>• **منظمة الصحة العالمية (WHO)**: تصف الشقيقة بأنها تترافق مع الغثيان والقيء في معظم الحالات.<br>• مواقع عربية موثوقة مثل وزارة الصحة السعودية وويب طب: تُدرج الغثيان والقيء كأعراض أساسية للشقيقة.<br><br>إذا كنت تعاني من هذه الأعراض بشكل متكرر، يُفضل استشارة طبيب للتشخيص الدقيق والعلاج المناسب، حيث توجد أدوية فعالة للسيطرة عليها (مثل مضادات الغثيان أو التريبتانات).
9	هل طنين الأذن أو آلام الرقبة لها علاقة بالشقيقة؟	نعم، هناك علاقة وثيقة بين **طنين الأذن** (الرنين أو الصفير في الأذنين) و**آلام الرقبة** من جهة، والشقيقة (الصداع النصفي أو الميغرين) من جهة أخرى، بناءً على دراسات طبية متعددة.<br><br>**طنين الأذن والشقيقة**<br><br>توجد ارتباط قوي بين الشقيقة وطنين الأذن. دراسات منشورة في مجلات طبية مثل PubMed وPMC

Chatbot 3 (Grok 4.1)		
تشير إلى أن:<br><br>• معظم الدراسات تظهر ارتباطاً بين الشقيقة وطنين الأذن، حيث يعاني ما يصل إلى 45% من مرضى الطنين من صداع نصفي كلاسيكي.<br>• خاصة في نوع يُسمى "الشقيقة الدهليزية" (vestibular migraine)، حيث يكون الطنين أحد الأعراض الشائعة، مصحوباً بدوار أو حساسية للصوت.<br>• الآلية المحتملة: تغيرات في تدفق الدم إلى الأذن الداخلية أو تنشيط عصبي مشترك بين الطرق السمعية والشقيقة.<br><br>طنين الأذن قد يحدث أثناء نوبات الشقيقة أو يكون مستقلاً، لكنه أكثر شيوعاً لدى مرضى الشقيقة مقارنة بالأشخاص العاديين.<br><br>**آلام الرقبة والشقيقة**<br><br>آلام الرقبة شائعة جداً لدى مرضى الشقيقة، وغالباً ما تكون عرضاً للشقيقة نفسها وليس سبباً منفصلاً:<br><br>• دراسات تشير إلى أن 75-90% من مرضى الشقيقة يعانون من آلام رقبة، خاصة في الشقيقة المزمنة (أكثر من 87%).<br>• الألم في الرقبة قد يكون في مرحلة الإنذار (prodrome) أو أثناء النوبة، ويزداد مع تكرار النوبات.<br>• الآلية: تقارب الأعصاب في الرقبة العلوية مع مسارات الألم في الشقيقة (trigeminocervical complex).<br>• ملاحظة مهمة: قد يُخلط بينها وبين "الصداع الرقبي المنشأ" (cervicogenic headache)، الذي ينشأ من مشاكل في الرقبة ويسبب صداعاً، لكن في معظم حالات الشقيقة، تكون آلام الرقبة عرضاً ثانوياً.<br><br>إذا كنت تعاني من هذه الأعراض معاً، فمن المحتمل أن تكون مرتبطة بالشقيقة، لكن يجب استشارة طبيب (مثل طبيب أعصاب أو أذن وأنف وحنجرة) لاستبعاد أسباب أخرى مثل مشاكل في الأذن الداخلية أو الفقرات الرقبية. العلاج غالباً يركز على إدارة الشقيقة (أدوية وقائية، تغييرات في نمط الحياة)، مما يحسن الطنين وآلام الرقبة أيضاً.		
**أسباب الشقيقة (الصداع النصفي أو الميغرين)**<br><br>السبب الدقيق للشقيقة غير معروف تمامًا، لكنه اضطراب عصبي معقد يتضمن عوامل **وراثية وبيئية وعصبية**. يُعتقد أن الشقيقة تنجم عن نشاط غير طبيعي في الدماغ يؤثر على الإشارات العصبية والكيماويات والأوعية الدموية في الدماغ، مما يؤدي إلى التهاب مؤلم وتوسع في الأوعية.<br><br>• **العامل الوراثي**: الشقيقة غالبًا ما تسري في العائلات، حيث يزداد خطر الإصابة إذا كان أحد الوالدين أو كلاهما مصابًا.<br>• **عوامل أخرى**: أكثر شيوعًا عند النساء (بسبب تقلبات هرمون الإستروجين)، وتبدأ عادة في سن المراهقة أو البلوغ المبكر.<br><br>**محفزات نوبات الشقيقة**	ما أسباب الشقيقة؟ وما هي محفزات النوبات؟	10

Chatbot 3 (Grok 4.1)		
المحفزات ليست أسبابًا مباشرة، بل عوامل تخفض عتبة الإصابة بنوبة لدى الأشخاص المعرضين وراثيًا. تختلف المحفزات من شخص لآخر، وقد يحتاج الأمر إلى مزيج منها لتحفيز النوبة. يُنصح بتدوين يوميات لتحديد المحفزات الشخصية. أبرز المحفزات الشائعة:<br><br>• **التوتر والضغط النفسي**: أكثر المحفزات شيوعًا (يؤثر على 70% من المصابين).<br>• **التغيرات الهرمونية**: خاصة عند النساء أثناء الدورة الشهرية، الحمل، أو سن اليأس، أو مع حبوب منع الحمل.<br>• **اضطرابات النوم**: قلة النوم، النوم الزائد، أو تغيير جدول النوم.<br>• **الأطعمة والمشروبات**:<br>○ الأجبان المعتقة، اللحوم المعالجة (مثل الهوت دوغ)، الأطعمة المالحة أو المضاف إليها مواد حافظة (مثل النترات أو MSG).<br>○ الشوكولاتة، المحليات الصناعية (مثل الأسبارتام).<br>○ الكحول (خاصة النبيذ الأحمر)، والكافيين الزائد أو الانسحاب منه.<br>• **تخطي الوجبات أو الجوع**: يؤدي إلى انخفاض سكر الدم.<br>• **المحفزات الحسية**: الأضواء الساطعة أو الوميضية، الأصوات العالية، الروائح القوية (مثل العطور أو الدخان).<br>• **التغيرات الجوية**: تغير الضغط الجوي، الحرارة الشديدة، الرطوبة العالية، أو العواصف.<br>• **الإجهاد البدني**: التمارين الشديدة المفاجئة، أو الإرهاق.<br><br>للوقاية، حافظ على نمط حياة منتظم (نوم، وجبات، شرب الماء)، وتجنب المحفزات المعروفة، واستشر طبيبًا لعلاج مناسب إذا تكررت النوبات.		
نعم، **الشقيقة (الصداع النصفي أو الميغرين)** لها مكون وراثي قوي، لكنها ليست مرضًا وراثيًا بحتًا بنمط بسيط مثل الأمراض الوراثية الأحادية الجين (monogenic). معظم الحالات الشائعة تكون متعددة الجينات (polygenic)، حيث تتفاعل عوامل وراثية متعددة مع عوامل بيئية ونمط حياة لتسبب المرض.<br><br>**الدليل على الجانب الوراثي:**<br><br>• تشير الدراسات إلى أن **الوراثة مسؤولة عن 34-60%** من خطر الإصابة بالشقيقة.<br>• إذا كان أحد الوالدين مصابًا، يرتفع خطر إصابة الابن أو الابنة إلى **50-75%**، وإذا كان كلا الوالدين مصابين، يصل الخطر إلى حوالي 75%.<br>• أكثر من نصف المصابين لديهم تاريخ عائلي للمرض.<br>• في أنواع نادرة مثل الشقيقة النصفية العائلية (familial hemiplegic migraine)، تكون وراثية أحادية الجين بنمط سائد.<br><br>**من هم الأكثر عرضة للإصابة؟**<br><br>الأشخاص الأكثر عرضة هم:<br><br>• **النساء**: تصيب النساء بنسبة ثلاث مرات أكثر من الرجال، غالبًا بسبب التغيرات الهرمونية (مثل الدورة الشهرية، الحمل، أو انقطاع الطمث).<br>• **الأشخاص ذوو التاريخ العائلي**: خاصة إذا كان هناك أقارب من الدرجة الأولى مصابون.<br>• **الأعمار بين 10-40 سنة**: تبدأ النوبات عادة في المراهقة وتشتد في الثلاثينيات، ثم تقل بعد ذلك.	هل الشقيقة مرض وراثي ومن هم الأكثر عرضة للإصابة؟	11

Chatbot 3 (Grok 4.1)		
• عوامل أخرى تزيد الخطر: التوتر، اضطرابات النوم، السمنة، الإفراط في الكافيين، والمحفزات البيئية مثل الأضواء الساطعة أو الأطعمة المعينة.<br><br>الشقيقة مرض معقد يتأثر بالجينات والبيئة معًا، وفهم العوامل الوراثية يساعد في التشخيص والعلاج الوقائي. إذا كنت تعاني من أعراض، استشر طبيبًا متخصصًا لتقييم حالتك الشخصية.		
وبات الشقيقة (الصداع النصفي) تختلف محفزاتها من شخص لآخر، وليست كل الأطعمة تسببها للجميع. وفقاً لمصادر طبية موثوقة مثل مؤسسة الشقيقة الأمريكية ومواقع صحية متخصصة، فإن **المحفزات الغذائية الحقيقية نادرة نسبياً**، وغالباً ما تكون الرغبة في بعض الأطعمة (مثل الشوكولاتة) علامة مبكرة للنوبة وليست سبباً لها. ومع ذلك، يبلغ بعض المرضى عن تحفيز نوبات بعد تناول أطعمة معينة، خاصة تلك التي تحتوي على مواد مثل **التيرامين**، **النترات**، أو **الكافيين**.<br><br>**الأطعمة الشائعة التي قد تحفز نوبات الشقيقة:**<br><br>1. **الكحول**، خاصة النبيذ الأحمر (أكثر المحفزات شيوعاً، يبلغ عنه حوالي 33% من المرضى).<br>2. **الشوكولاتة** (يبلغ عنها حوالي 22%، لكن قد تكون رغبة سابقة للنوبة).<br>3. **الأجبان القديمة أو المعتقة** (مثل الشيدر، البارميزان، الجبن الأزرق، الفيتا) بسبب التيرامين.<br>4. **اللحوم المصنعة أو المعالجة** (مثل النقانق، الهوت دوغ، السلامي، البيكون) بسبب النترات والنتريت.<br>5. **الكافيين** (الإفراط فيه أو الانسحاب المفاجئ منه، مثل القهوة أو الشاي).<br>6. **المحليات الصناعية** (مثل الأسبارتام في المشروبات الدايت).<br>7. **الغلوتامات أحادية الصوديوم (MSG)** (موجودة في الأطعمة الآسيوية المعلبة أو البهارات).<br>8. **الأطعمة المخمرة أو المخللة** (مثل الملفوف المخلل، الزيتون، صلصة الصويا).<br>9. **بعض الفواكه** (مثل الحمضيات، الموز، الأفوكادو، الفواكه المجففة).<br><br>**نصائح هامة:**<br><br>• **احتفظ بيومية غذائية**: سجل ما تأكله ومتى تحدث النوبات لتحديد محفزاتك الشخصية.<br>• **لا تقصِ كل شيء دفعة واحدة**: جرب تجنب مجموعة واحدة لأسابيع ولاحظ التغيير.<br>• **الأهم هو الانتظام**: تناول وجبات منتظمة، شرب كمية كافية من الماء، وتجنب تخطي الوجبات، حيث يُعتبر انخفاض السكر في الدم محفزاً قوياً.<br>• استشر طبيباً متخصصاً في الصداع لتقييم حالتك، فقد لا تكون الأطعمة السبب الرئيسي.<br><br>هذه المعلومات مبنية على تقارير المرضى ودراسات، لكن الأدلة العلمية القوية محدودة، والمحفزات فردية جداً.	ما الأطعمة التي قد تحفز نوبات الشقيقة؟	12
يتم تشخيص **الشقيقة** (الصداع النصفي أو الـ Migraine) بشكل أساسي بناءً على التاريخ المرضي والأعراض التي يصفها المريض، بالإضافة إلى الفحص السريري والعصبي، دون الحاجة إلى اختبار مخبري محدد، حيث لا يوجد فحص دم أو تصوير يؤكد التشخيص مباشرة.	كيف يتم تشخيص الشقيقة؟	13

Chatbot 3 (Grok 4.1)

الخطوات الرئيسية في التشخيص:

1. **التاريخ المرضي والأعراض**: يعتمد الطبيب (عادة طبيب أعصاب أو طبيب عام) على وصف المريض للنوبات، ويسأل عن:
   - عدد النوبات السابقة.
   - مدة كل نوبة (عادة 4-72 ساعة إذا لم تعالج).
   - شدة الألم ومكانه (غالبًا في جانب واحد من الرأس، نابض).
   - الأعراض المصاحبة مثل الغثيان، القيء، حساسية للضوء أو الصوت، أو اضطرابات بصرية (أورة).
2. **المعايير التشخيصية الدولية ((ICHD-3**: حسب تصنيف الصداع الدولي (من جمعية الصداع الدولية)، يُشخص الصداع النصفي غير المصحوب بالأورة إذا تحققت:
   - **خمس نوبات على الأقل.**
   - كل نوبة تستمر 4-72 ساعة.
   - الألم يحمل **اثنتين على الأقل** من الخصائص التالية:
     - في جانب واحد من الرأس.
     - نابض أو خافق.
     - متوسط إلى شديد الشدة.
     - يزداد مع النشاط البدني العادي (مثل المشي أو صعود الدرج).
   - مصاحب **لواحد على الأقل** مما يلي:
     - غثيان و/أو قيء.
     - حساسية للضوء (رهاب الضوء) و/أو الصوت (رهاب الصوت).
   - لا يُفسر بمرض آخر.
3. أما الشقيقة المصحوبة بالأورة (حوالي 25-30% من الحالات)، فيكفي نوبتان، مع أعراض عصبية مؤقتة قبل أو أثناء الصداع (مثل رؤية ومضات ضوئية أو خدر في الأطراف).
4. **الفحص السريري والعصبي**: يكون طبيعيًا عادةً في حالات الشقيقة النموذجية، ويساعد في استبعاد أسباب أخرى.
5. **فحوصات إضافية لاستبعاد أسباب أخرى**: لا تُطلب روتينيًا، لكن إذا كانت الأعراض غير نموذجية أو هناك علامات تحذيرية (مثل صداع مفاجئ شديد، أو بعد عمر 50، أو مصحوب بضعف عضلي أو حمى)، قد يطلب الطبيب:
   - تحاليل دم (لاستبعاد الالتهابات أو مشاكل أخرى).
   - تصوير مقطعي محوسب (CT) أو رنين مغناطيسي (MRI) لاستبعاد أورام، نزيف، أو سكتة دماغية.
   - في حالات نادرة: بزل قطني أو مخطط كهربية الدماغ (EEG).

نصائح هامة:

- يُفضل تسجيل يوميات الصداع (تاريخ النوبات، مدتها، المحفزات مثل التوتر أو بعض الأطعمة) لمساعدة الطبيب.
- الشقيقة غالبًا وراثية، فإذا كان هناك تاريخ عائلي، يدعم التشخيص.
- استشر الطبيب فورًا إذا كان الصداع جديدًا، شديدًا جدًا، أو مصحوبًا بأعراض مقلقة مثل فقدان الوعي أو ضعف في جانب الجسم.

التشخيص المبكر يساعد في إدارة الحالة بشكل أفضل، فلا تتردد في زيارة الطبيب إذا تكررت النوبات.

<table>
<tr><th colspan="3">Chatbot 3 (Grok 4.1)</th></tr>
<tr>
<td>14</td>
<td>ما مدى شيوع الشقيقة؟</td>
<td>الشقيقة (الصداع النصفي أو Migraine) هي اضطراب عصبي شائع جداً على مستوى العالم.

الانتشار العالمي:
• يُقدر أن الشقيقة تصيب حوالي 14-15% من سكان العالم، أي أكثر من مليار شخص (حوالي 1.16 مليار حالة في عام 2021، وفقاً لدراسة Global Burden of Disease - GBD 2021).
• هي أكثر شيوعاً لدى النساء (تصيب حوالي 18-19% منهن) مقارنة بالرجال (حوالي 9-10%)، وتكون ذروتها في سن الإنتاجية (30-49 عاماً).
• تعد الشقيقة ثاني أو ثالث أكبر سبب للإعاقة عالمياً (من حيث سنوات العمر المفقودة بسبب الإعاقة - YLDs)، خاصة لدى الشابات.

الانتشار في الدول العربية والشرق الأوسط:
• يتراوح الانتشار بين 2.6% و32% حسب الدراسات المختلفة، مع متوسط يقترب من الانتشار العالمي (حوالي 10-15% في معظم الدراسات الحديثة).
• هي أكثر شيوعاً لدى النساء بنسبة 2-2.5 مرات مقارنة بالرجال، وغالباً ما تكون غير مشخصة أو غير معالجة بشكل كافٍ.
• دراسات حديثة في دول مثل السعودية، الكويت، والعراق تشير إلى انتشار يصل إلى 20-37% في بعض الفئات (مثل الشباب أو الطلاب).

الشقيقة ليست مجرد "صداع عادي"، بل تسبب إعاقة كبيرة وتؤثر على جودة الحياة، وغالباً ما تكون مرتبطة ب عوامل مثل التوتر، اضطراب النوم، والتغيرات الهرمونية. إذا كنت تعاني من أعراضها، يُنصح باستشارة طبيب متخصص للتشخيص والعلاج المناسب.</td>
</tr>
<tr>
<td>15</td>
<td>في أي عمر تبدأ نوبات الشقيقة؟</td>
<td>نوبات الشقيقة (الصداع النصفي أو الmigraine) يمكن أن تبدأ في أي عمر، لكنها غالباً ما تظهر لأول مرة في سن مبكرة.

• تبدأ عند العديد من الأشخاص خلال الطفولة أو المراهقة، حيث يُبلغ عن حالات حتى عند الأطفال الصغار (من عمر 18 شهراً أو أقل من 3 سنوات في بعض الحالات النادرة).
• يحدث النصف الأول من الحالات قبل عمر 12 عاماً، ومعظم الحالات تبدأ قبل عمر 25-35 عاماً.
• يكون الذروة في الانتشار والشدة بين عمر 20-40 عاماً (خاصة في الثلاثينيات)، ثم تقل تدريجياً التكرار والشدة مع التقدم في العمر، خاصة بعد عمر 50 عاماً.
• نادراً ما تبدأ نوبات جديدة بعد عمر 40-50 عاماً، وإذا حدثت في عمر متأخر (مثل بعد 50 أو 60)، يُفضل استشارة الطبيب لاستبعاد أسباب أخرى.

هذه المعلومات مستمدة من مصادر طبية موثوقة مثل Mayo Clinic، American Headache Society، ودراسات إحصائية عن انتشار الشقيقة. إذا كنت تعاني من نوبات، يُنصح بزيارة طبيب أعصاب للتشخيص الدقيق والعلاج المناسب.</td>
</tr>
</table>

<table>
<tr><th colspan="3">Chatbot 3 (Grok 4.1)</th></tr>
<tr>
<td>16</td>
<td>هل يمكن أن يصاب الأطفال بالشقيقة؟</td>
<td>
نعم، يمكن أن يصاب الأطفال بالشقيقة (الصداع النصفي أو Migraine)، وهي حالة شائعة جدًا في مرحلة الطفولة، حيث تُصيب حوالي 3-11% من الأطفال في سن ما قبل المدرسة والمدرسة الابتدائية، وترتفع النسبة إلى 8-15% (أو حتى 28% في بعض الدراسات) بين الأطفال في سن المدرسة الثانوية أو المراهقين. وفقًا لمصادر طبية موثوقة مثل Cleveland Clinic و NCBI (National Center for Biotechnology Information)، يبدأ الصداع النصفي عادةً قبل سن 12 عامًا، وقد سُجلت حالات لدى أطفال أقل من 18 شهرًا، بل وحتى الرضع في بعض الحالات النادرة. الشقيقة ليست مجرد صداع عادي، بل مرض عصبي وراثي يسبب ألمًا شديدًا نابضًا، وغالبًا ما يستمر من 2 إلى 48 ساعة لدى الأطفال (أقصر من البالغين الذين يصل إلى 72 ساعة)، ويؤثر على الحياة اليومية مثل الذهاب إلى المدرسة أو اللعب.

انتشار الشقيقة عند الأطفال (بناءً على دراسات حديثة):

أقل من 7 سنوات: 2.5-3% (متساوٍ بين الذكور والإناث تقريبًا).
7-11 سنة: 4-11% (أكثر شيوعًا لدى الذكور قبل البلوغ).
12-18 سنة: 10-28% (تزداد لدى الإناث بعد البلوغ بنسبة 2-3 أضعاف بسبب التغيرات الهرمونية).
إحصائيات عالمية: حوالي 1 من كل 10 أطفال يعاني من نوبات متكررة، وفقًا لـ American Migraine Foundation ودراسات NIH. في الولايات المتحدة، يفقد الأطفال 329,000 يوم دراسي شهريًا بسببها.

هذه النسب مدعومة بدراسات وبائية واسعة مثل تلك المنشورة في StatPearls (NCBI) وCleveland Clinic، حيث يُقدر أن 50% من الأطفال المصابين يستمرون في الإصابة بالشقيقة في سن البالغين إذا لم يُعالج السبب مبكرًا.

الأعراض عند الأطفال (تختلف قليلاً عن البالغين):

الشقيقة عند الأطفال غالبًا ثنائية الجانبين (في كلا جانبي الرأس أو الجبهة)، وليست جانبًا واحدًا كما في البالغين، وقد تكون مصحوبة بـ:

ألم نابض شديد: يزداد مع الحركة أو النشاط.
غثيان، قيء، إسهال، أو ألم بطن (شائع جدًا، خاصة في "الشقيقة البطنية" عند الصغار).
حساسية للضوء (فوتوفوبيا) أو الصوت (فونوفوبيا): الطفل يبحث عن غرفة مظلمة هادئة.
شحوب الوجه، دوار، إرهاق، أو تهيج قبل النوبة (مرحلة الـ"prodrome" في 67% من الحالات).
أورا (نادرة عند الصغار): ومضات ضوئية، نقاط عمياء، أو تنميل مؤقت.
نوبات قصيرة: 1-24 ساعة، وتنتهي غالبًا بالنوم.

قد لا يصف الطفل الألم جيدًا، فيظهر كبكاء مستمر أو رفض الطعام/اللعب (Mayo Clinic).

الأسباب والمحفزات (وراثية بنسبة عالية):

وراثية: إذا كان أحد الوالدين مصابًا، احتمال إصابة الطفل 50%؛ إذا كان كلا الوالدين، 90% (altibbi.com، WebMD، American Migraine Foundation)). تغييرات في جينات مثل CACNA1A أو 2ATP1A.
محفزات شائعة عند الأطفال (تختلف من طفل لآخر، سجل يوميات لتحديدها):

</td>
</tr>
</table>

Chatbot 3 (Grok 4.1)

محفز	نسبة الشيوع
قلة النوم أو اضطراباته	40-65%
التوتر/القلق/التغييرات المدرسية	25-45%
أطعمة معينة (شوكولاتة، جبن، كافيين، أطعمة مصنعة)	20-30%
تغيرات الطقس/الحرارة	20-40%
صوت عالٍ/أضواء ساطعة/شاشات	20-30%
جوع أو تفويت وجبات	15-25%
روائح قوية أو حركة (سيارة)	10-20%

(مصادر: neuroarabia.com، HealthyChildren.org، Harvard Health).

العلاج والوقاية:

- **علاج النوبة الحادة:**
  - إيبوبروفين أو باراسيتامول فورًا (فعال في 70% إذا أُعطي مبكرًا).
  - تريبتانات (مثل سوماتريبتان) للأكبر من 6 سنوات (معتمدة من FDA).
  - راحة في غرفة مظلمة، كمادات باردة، سوائل، تجنب الشاشات.
- **الوقاية (إذا >2-4 نوبات شهريًا):**
  - تغييرات نمط حياة: نوم منتظم (8-10 ساعات)، وجبات متوازنة، رياضة، تجنب محفزات، إدارة التوتر (يوغا/تأمل).
  - أدوية وقائية: أميتريبتيلين أو توبيرامات (تحت إشراف طبيب أعصاب أطفال).
  - مكملات: ريبوفلافين، مغنيسيوم، CoQ10 (فعالة في بعض الدراسات).
- **متى تزور الطبيب فورًا؟** إذا كان الصداع الأسوأ على الإطلاق، مع حمى، ضعف، تغيرات في الرؤية، أو يوقظ الطفل ليلاً (لاستبعاد أورام أو التهابات، لكن نادر).

الشقيقة ليست خطيرة عضويًا، لكنها تؤثر نفسيًا وتُعيق الدراسة؛ العلاج المبكر يقلل النوبات بنسبة 50-70% (دراسات NIH). استشر طبيب أطفال أو أعصاب لتشخيص دقيق بناءً على تاريخ عائلي ويوميات الصداع.

<table>
<tr><th colspan="3">Chatbot 3 (Grok 4.1)</th></tr>
<tr><td>17</td><td>هل الشقيقة مرض مزمن؟</td><td>
نعم، **الشقيقة (الصداع النصفي أو Migraine)** تعتبر عموماً **مرضاً مزمناً**، حيث إنها اضطراب عصبي يستمر مدى الحياة لدى معظم المصابين، مع نوبات متكررة قد تستمر سنوات أو عقوداً، ولا يوجد علاج شافٍ نهائياً، بل يركز العلاج على السيطرة على النوبات وتقليل تكرارها.

ومع ذلك، هناك تمييز مهم:

• **الشقيقة الانتيابية (Episodic Migraine)**: تحدث النوبات بشكل متقطع (أقل من 15 يوماً في الشهر)، وهي الشكل الأكثر شيوعاً.
• **الشقيقة المزمنة (Chronic Migraine)**: تصنف كذلك إذا حدث الصداع لمدة **15 يوماً أو أكثر في الشهر** لأكثر من 3 أشهر، مع أن تكون **8 أيام على الأقل** منها نوبات شقيقة حقيقية (وليست صداعاً عادياً).

هذا التعريف مقبول عالمياً حسب تصنيف الجمعية الدولية للصداع (ICHD-3)، ومصادر طبية موثوقة مثل ويكيبيديا، ويب طب، الطبي، وMSD Manuals.

الشقيقة غالباً ما تبدأ في سن المراهقة أو الشباب، وتقل شدتها أو تختفي لدى البعض بعد سن الـ50، لكنها تبقى حالة مزمنة لدى الكثيرين. إذا كنت تعاني من أعراض متكررة، يُفضل استشارة طبيب أعصاب لتقييم الحالة ووصف علاج وقائي أو مسكن مناسب.
</td></tr>
<tr><td>18</td><td>ما مضاعفات الشقيقة؟</td><td>
مضاعفات الشقيقة (الصداع النصفي)

الشقيقة أو الصداع النصفي هو حالة عصبية شائعة، وغالباً ما تكون غير خطيرة بحد ذاتها، لكنها قد تؤدي إلى مضاعفات متنوعة، بعضها ناتج عن النوبات المتكررة أو سوء الإدارة، وبعضها نادر وخطير. إليك أبرز المضاعفات بناءً على مصادر طبية موثوقة مثل Mayo Clinic، WebMD، ومواقع عربية مثل ويب طب والطبي:

1. **الصداع المرتد (Medication Overuse Headache أو Rebound Headache)**: يحدث بسبب الإفراط في تناول مسكنات الألم (مثل الإيبوبروفين أو الأسبرين أكثر من 2-3 مرات أسبوعياً)، مما يجعل الأدوية نفسها تسبب صداعاً جديداً ويدخل المريض في دائرة مغلقة.
2. **متلازمة السيروتونين (Serotonin Syndrome)**: حالة نادرة ولكنها قد تكون مهددة للحياة، تحدث عند تفاعل أدوية الشقيقة (مثل التريبتانات) مع مضادات الاكتئاب التي ترفع مستويات السيروتونين، وتسبب أعراضاً مثل الارتباك، الإسهال، الارتعاش، وتسارع ضربات القلب.
3. **الحالة الشقيقية المستمرة (Status Migrainosus)**: نوبة شقيقة تستمر أكثر من 72 ساعة، تسبب ألماً شديداً وغثياناً مستمراً، وقد تؤدي إلى الجفاف وتتطلب دخول المستشفى.
4. **الاحتشاء الشقيقي (Migrainous Infarction)**: مضاعفة نادرة جداً، حيث يحدث سكتة دماغية إقفارية أثناء نوبة شقيقة (خاصة مع الهالة)، وتزداد المخاطر لدى النساء الشابات.
5. **الصداع النصفي المزمن (Chronic Migraine)**: يحدث عندما تتجاوز النوبات 15 يوماً في الشهر لأكثر من 3 أشهر، مما يؤثر بشكل كبير على جودة الحياة والإنتاجية.
6. **مشاكل الجهاز الهضمي**: مثل قرحة المعدة، النزيف، أو آلام البطن بسبب الإفراط في مضادات الالتهاب غير الستيرويدية.
7. **مضاعفات أخرى مرتبطة**:
</td></tr>
</table>

Chatbot 3 (Grok 4.1)

		○ زيادة خطر الإصابة بالاكتئاب، القلق، اضطرابات النوم، وأمراض القلب أو السكتة الدماغية (خاصة في الشقيقة مع هالة).<br>○ نوبات صرع محفزة بالشقيقة (نادرة).<br>○ هالة مستمرة بدون احتشاء.<br><br>معظم هذه المضاعفات يمكن تجنبها بالعلاج الوقائي المناسب، تجنب الإفراط في الأدوية، واستشارة طبيب أعصاب. إذا كنت تعاني من نوبات متكررة أو أعراض جديدة (مثل ضعف عضلي أو اضطرابات بصرية مفاجئة)، استشر الطبيب فوراً لاستبعاد حالات أخرى خطيرة.
19	هل تتحسن الشقيقة مع التقدم بالعمر؟	نعم، في معظم الحالات **تتحسن الشقيقة (الصداع النصفي أو الميغرين)** مع التقدم في العمر، خاصة بعد سن الـ50 أو مع انقطاع الطمث عند النساء.<br><br>**التفاصيل الرئيسية:**<br><br>● **ذروة الإصابة**: يصل الصداع النصفي إلى أعلى شدة وانتشار بين سن الـ20 والـ40 عامًا، حيث يكون أكثر تكرارًا وشدة.<br>● **التحسن مع العمر**: بعد سن الـ40، تبدأ النوبات في الانخفاض تدريجيًا في التكرار والشدة، وتصبح أكثر استجابة للعلاج. بعد سن الـ60، غالبًا ما تقل النوبات بشكل ملحوظ أو تختفي تمامًا لدى الكثيرين.<br>● **دور انقطاع الطمث (المنوبوز)**: عند النساء، يرتبط التحسن غالبًا بانخفاض مستويات الإستروجين المتقلبة. في معظم الدراسات، يتحسن الصداع لدى ثلثي النساء تقريبًا بعد انقطاع الطمث الطبيعي، وقد يختفي تمامًا لدى بعضهن. ومع ذلك، قد يزداد سوءًا مؤقتًا أثناء فترة ما قبل انقطاع الطمث (البريمنوبوز) بسبب تقلبات الهرمونات.<br>● **استثناءات**: ليس التحسن مضمونًا للجميع. قد يستمر الصداع لدى بعض الأشخاص (حوالي ثلث النساء بعد المنوبوز)، أو يبدأ لأول مرة في سن متأخرة، خاصة إذا كان انقطاع الطمث جراحيًا (مثل استئصال المبيضين)، حيث قد يزداد سوءًا في بعض الحالات.<br><br>هذا التحسن مدعوم بدراسات وبائية واسعة، مثل دراسات الانتشار العالمي للمرض، التي تظهر انخفاضًا واضحًا في معدلات الإصابة مع التقدم في السن. ومع ذلك، يُنصح دائمًا باستشارة طبيب أعصاب لتقييم الحالة الشخصية، خاصة إذا كان الصداع مستمرًا أو متغيرًا.
20	ما علاج نوبات الشقيقة؟ و متى يجب أخذ العلاج؟	علاج نوبات الشقيقة (الصداع النصفي) ينقسم إلى نوعين رئيسيين: **علاج النوبة الحادة** (لإيقاف النوبة عند حدوثها) و**علاج الوقاية** (لتقليل تكرار النوبات). لا يوجد علاج شافٍ تمامًا، لكن العلاجات تساعد في السيطرة على الأعراض.<br><br>**علاج النوبة الحادة (عند حدوث الشقيقة):**<br><br>● **مسكنات الألم البسيطة** (للنوبات الخفيفة إلى المتوسطة): مثل الإيبوبروفين (400-600 мг)، النابروكسين، أو الأسبرين (900-1000 мг)، أو الباراسيتامول (أسيتامينوفين). غالبًا ما تكون فعالة إذا أخذت مبكرًا.<br>● **التريبتانات** (للنوبات المتوسطة إلى الشديدة): مثل السوماتريبتان، الريزاتريبتان، أو

Chatbot 3 (Grok 4.1)		
الزولميتريبتان. هذه الأدوية المخصصة للشقيقة تعمل على تضييق الأوعية الدموية وتثبيط الإشارات العصبية، وتُعتبر الأكثر فعالية حسب الإرشادات الحديثة (مثل تلك من الجمعية الأمريكية للصداع).<br>• **أدوية جديدة (جيبانتس أو مثبطات CGRP)**: مثل الريميجيبانت أو الأوبروجيبانت، فعالة خاصة إذا لم تنجح التريبتانات.<br>• **أدوية مضادة للغثيان**: مثل الميتوكلوبراميد، إذا كانت النوبة مصحوبة بغثيان أو قيء.<br><br>**متى يجب أخذ العلاج؟**<br><br>• **الأهم: في أسرع وقت ممكن**، عند بداية الأعراض أو عندما يكون الألم خفيفًا هنوز. التأخير يقلل من فعالية العلاج، حيث يصبح الدماغ أكثر حساسية مع تقدم النوبة.<br>• لا تنتظر حتى يشتد الألم؛ خذ الدواء فور الشعور بالعلامات الأولى (مثل الهالة إن وجدت، أو بداية الخفقان).<br>• إذا كانت النوبات متكررة (أكثر من 4 أيام شهريًا)، استشر الطبيب لعلاج وقائي.<br><br>**علاج الوقاية (إذا كانت النوبات متكررة أو شديدة):**<br><br>يُستخدم يوميًا لتقليل عدد النوبات، مثل:<br><br>• حاصرات بيتا (مثل البروبرانولول).<br>• مضادات الاختلاج (مثل التوبيرامات).<br>• مضادات CGRP (حقن شهرية مثل الإرينوماب).<br>• أحيانًا البوتوكس للحالات المزمنة.<br><br>**نصائح عامة:**<br><br>• تجنب المحفزات (مثل التوتر، نقص النوم، بعض الأطعمة).<br>• احتفظ بيومية لتسجيل النوبات والمحفزات.<br>• **مهم جدًا**: استشر طبيبًا متخصصًا في الصداع (عصبيات) لتحديد العلاج المناسب، حيث يختلف الاستجابة من شخص لآخر، وتجنب الإفراط في المسكنات لتفادي "صداع الإفراط في الدواء".<br><br>المصادر الطبية الموثوقة مثل Mayo Clinic وMSD Manuals تؤكد أن العلاج المبكر هو المفتاح للنجاح. إذا استمرت النوبات، قد يحتاج الأمر إلى فحوصات إضافية.		
الأدوية الوقائية لنوبات الشقيقة (الصداع النصفي) تهدف إلى تقليل تكرار النوبات وشدتها، خاصة إذا كانت النوبات متكررة (أكثر من 4 نوبات شهرياً أو تؤثر على الحياة اليومية). يجب دائماً استشارة الطبيب قبل البدء بأي علاج، حيث يعتمد الاختيار على الحالة الصحية، الأمراض المصاحبة، والآثار الجانبية المحتملة.<br><br>**الأدوية الوقائية الرئيسية (بناءً على الإرشادات الطبية الحديثة مثل Mayo Clinic، AAN، وACP حتى 2025):**<br><br>1. **حاصرات بيتا (Beta-blockers):**<br>○ بروبرانولول (Propranolol)	ما الأدوية الوقائية لنوبات الشقيقة؟	21

Chatbot 3 (Grok 4.1)		
○ ميتوبرولول (Metoprolol)<br>○ تيمولول (Timolol)<br>○ أتينولول (Atenolol) أو نادولول (Nadolol) فعالة جداً وغالباً ما تكون الخيار الأول، خاصة إذا كان هناك ارتفاع في ضغط الدم.<br>2. **مضادات الاختلاج (Antiseizure medications):**<br>○ توبيرامات (Topiramate)<br>○ فالبروات أو ديفالبروكس (Valproate/Divalproex) فعالة، لكن غير موصى بها للنساء الحوامل بسبب مخاطر على الجنين.<br>3. **مضادات الاكتئاب:**<br>○ أميتريبتيلين (Amitriptyline)<br>○ فينلافاكسين (Venlafaxine) مفيدة خاصة إذا كان هناك اكتئاب أو قلق مصاحب.<br>4. **علاجات حديثة تستهدف الببتيد المرتبط بجين الكالسيتونين (CGRP inhibitors):**<br>○ أجسام مضادة أحادية النسيلة (Monoclonal antibodies): إرينوماب (Erenumab)، فريمانيزوماب (Fremanezumab)، غالكانيزوماب (Galcanezumab)، إبتينيزوماب (Eptinezumab) – تعطى عن طريق الحقن الشهري أو كل 3 أشهر.<br>○ غيبانتس (Gepants): أتوغيبانت (Atogepant) أو ريميغيبانت (Rimegepant) – أقراص يومية. هذه العلاجات الحديثة فعالة جداً وآثارها الجانبية أقل، وغالباً ما تُستخدم إذا فشلت الأدوية التقليدية.<br>5. **أخرى:**<br>○ بوتوكس (OnabotulinumtoxinA): للشقيقة المزمنة (أكثر من 15 يوم صداع شهرياً).<br>○ كانديسارتان (Candesartan) أو ليسينوبريل (Lisinopril): من أدوية ضغط الدم.<br>○ فيراباميل (Verapamil): حاصر قنوات الكالسيوم، خاصة للشقيقة مع هالة.<br><br>**نصائح عامة:**<br><br>● تبدأ الفعالية عادة بعد أسابيع إلى أشهر، وقد يحتاج الطبيب إلى تجربة عدة أدوية للعثور على الأنسب.<br>● تجنب الإفراط في مسكنات الألم الحادة لتجنب "صداع الإفراط في الدواء".<br>● دمج العلاج الدوائي مع تغييرات في نمط الحياة (نوم منتظم، شرب ماء كافٍ، تجنب المحفزات مثل التوتر أو بعض الأطعمة) يعزز النتائج.<br><br>هذه المعلومات مبنية على مصادر موثوقة مثل Mayo Clinic وإرشادات الجمعيات الطبية، لكنها ليست بديلاً عن استشارة طبيب متخصص في الصداع أو طبيب أعصاب.		
عم، يمكن تخفيف أعراض الشقيقة (الصداع النصفي) أو تقليل تكرار نوباتها باستخدام طرق منزلية وبديلة، لكنها لا تشفي المرض تمامًا. هذه الطرق غالبًا ما تكون مكملة للعلاج الطبي، وتعتمد فعاليتها على الأدلة العلمية المحدودة من مصادر موثوقة مثل WebMD، Mayo Clinic، و American Migraine Foundation. دائمًا استشر طبيبًا قبل تجربة أي علاج جديد، خاصة إذا كانت النوبات شديدة أو متكررة.<br><br>**طرق منزلية لتخفيف الأعراض أثناء النوبة:**<br><br>● **الراحة في غرفة مظلمة وهادئة:** ابتعد عن الضوء الساطع والأصوات العالية، ونام إن أمكن. هذا	هل يمكن علاج الشقيقة بطرق منزلية أو بديلة؟	22

<table>
<tr><th colspan="3">Chatbot 3 (Grok 4.1)</th></tr>
<tr><td></td><td></td><td>
يقلل من تفاقم الألم.
• **كمادات باردة أو ساخنة**: ضع كيس ثلج أو قماش بارد على الجبهة أو الرقبة لتخدير الألم، أو كمادة ساخنة لإرخاء العضلات.
• **شرب كميات كافية من الماء**: الجفاف يُحفز النوبات، فاشرب الماء بانتظام.
• **تناول الزنجبيل**: شاي الزنجبيل أو مضغ قطعة طازجة يساعد في تقليل الغثيان والألم (دراسات أظهرت فعاليته مشابهة لبعض الأدوية).
• **كمية صغيرة من الكافيين**: فنجان قهوة أو شاي قد يخفف الألم في البداية، لكن تجنب الإفراط لأنه قد يسبب نوبات لاحقة.

طرق بديلة ووقائية لتقليل التكرار:

• **تجنب المحفزات الشائعة**: احتفظ بيومية لتسجيل النوبات والأطعمة/الظروف المرتبطة بها. المحفزات الشائعة تشمل:
  ○ التوتر (مارس الاسترخاء أو اليوغا).
  ○ نقص النوم أو الإفراط فيه (حافظ على جدول نوم منتظم).
  ○ بعض الأطعمة مثل الجبن القديم، الشوكولاتا، الكحول (خاصة النبيذ الأحمر)، والأطعمة المعالجة.
  ○ التغيرات الهرمونية (خاصة عند النساء).
• **المكملات الغذائية** (بإشراف طبي):
  ○ المغنيسيوم (400-600 ملغ يوميًا): يقلل التكرار لدى الكثيرين.
  ○ فيتامين 2B (ريبوفلافين): جرعات عالية تساعد في الوقاية.
  ○ 10CoQ: يقلل الشدة والتكرار.
• **العلاجات البديلة الأخرى**:
  ○ الإبر الصينية (Acupuncture): أدلة جيدة على تقليل التكرار.
  ○ اليوغا أو التأمل: تقلل التوتر وتحسن السيطرة على الألم.
  ○ تدليك الرأس أو الضغط على نقاط معينة (Acupressure).

هذه الطرق فعالة لدى بعض الأشخاص، لكن النتائج تختلف. إذا كانت الشقيقة تؤثر على حياتك اليومية، فكر في العلاج الدوائي الوقائي أو زيارة متخصص في الصداع.
</td></tr>
<tr><td>23</td><td>ما خيارات علاج الشقيقة أثناء الحمل؟</td><td>
خيارات علاج الشقيقة (الصداع النصفي) أثناء الحمل تتطلب حذراً كبيراً لضمان سلامة الأم والجنين. غالباً ما تتحسن الشقيقة خلال الحمل، خاصة في الثلث الثاني والثالث، بسبب استقرار مستويات الهرمونات، لكن في بعض الحالات قد تستمر أو تزداد. **يجب دائماً استشارة الطبيب قبل أي علاج**، حيث يقيم الطبيب الفوائد مقابل المخاطر بناءً على حالتك.

1. العلاجات غير الدوائية (الخيار الأول والأفضل)

يُفضل البدء بها دائماً، وفقاً لإرشادات منظمات مثل الجمعية الأمريكية للصداع وACOG:

• تجنب المحفزات المعروفة (مثل بعض الأطعمة، التوتر، الروائح القوية، نقص النوم).
• الراحة في غرفة مظلمة وهادئة.
• كمادات باردة أو دافئة على الرأس.
• شرب كميات كافية من الماء (الترطيب الجيد).
• نوم منتظم ووجبات منتظمة.
</td></tr>
</table>

Chatbot 3 (Grok 4.1)		
• تمارين استرخاء، بيوفيدباك، يوغا، أو تدليك.<br>• تمارين رياضية خفيفة (مثل المشي).<br>• الإبر الصينية (أكيوبونكتشر) أو العلاج بالتحفيز العصبي غير الغازي (في بعض الحالات).<br><br>هذه الطرق آمنة تماماً وفعالة في كثير من الحالات.<br><br>**2. العلاجات الدوائية للنوبات الحادة (عند الحاجة)**<br><br>• **الباراسيتامول (أسيتامينوفين أو بانادول)**: الخيار الأول والأكثر أماناً في جميع مراحل الحمل. جرعة تصل إلى 1000 мг، ويمكن دمجه مع كافيين قليل (أقل من 200 мг يومياً).<br>• **مضادات الالتهاب غير الستيرويدية (مثل إيبوبروفين)**: قد تكون آمنة في الثلث الثاني فقط (أسابيع 13-28 تقريباً)، وتجنبها في الثلث الأول والثالث.<br>• **سوماتريبتان (من عائلة التريبتان)**: يُعتبر خياراً ثانياً في حال عدم استجابة للباراسيتامول، خاصة أنه الأكثر دراسة ولا يرتبط بزيادة كبيرة في العيوب الخلقية، لكن يُستخدم بحذر وبعد استشارة طبيب.<br>• **أدوية مضادة للغثيان**: مثل ميتوكلوبراميد أو بروكلوربيرازين، آمنة نسبياً.<br>• **حقن الأعصاب الطرفية (نيرف بلوك)**: باستخدام ليدوكائين، خيار آمن في بعض الحالات الشديدة.<br><br>**3. العلاج الوقائي (إذا كانت النوبات متكررة وشديدة)**<br><br>نادر الحاجة، لكن إذا لزم:<br><br>• مكملات مثل المغنيسيوم أو فيتامين 2B (ريبوفلافين).<br>• بعض حاصرات بيتا (مثل بروبرانولول).<br>• مضادات الهيستامين أو حاصرات قنوات الكالسيوم.<br><br>**ما يجب تجنبه تماماً:**<br><br>• الإرغوتامين والديهيدروإرغوتامين (ممنوعة).<br>• بعض الأدوية مثل فالبروات، توبيرامات، أو أدوية CGRP الجديدة (غير آمنة).<br>• الأسبرين بجرعات عالية، أو الأفيونيات إلا في حالات نادرة.<br><br>في النهاية، العلاج فردي، وقد يحتاج الأمر إلى متابعة مع طبيب أعصاب أو متخصص في الصداع. إذا كانت النوبات شديدة أو مصحوبة بأعراض غير عادية (مثل ضعف في الرؤية المفاجئ أو ارتفاع ضغط الدم)، توجهي فوراً إلى الطوارئ لاستبعاد مشاكل أخرى مثل تسمم الحمل.		
لا، **لا يمكن الشفاء من الشقيقة (الصداع النصفي أو الميغرين) بشكل نهائي** في الوقت الحالي، حسب الإجماع الطبي من مصادر موثوقة مثل Mayo Clinic، WebMD، NIH، ومنظمات صحية أخرى.<br><br>الصداع النصفي هو حالة مزمنة عصبية وراثية في الغالب، ولا يوجد علاج يقضي عليها تماماً. ومع ذلك، يمكن السيطرة عليها بشكل فعال جداً لدى معظم المرضى، مما يقلل من تكرار النوبات وشدتها، وقد يؤدي إلى فترات طويلة خالية من الأعراض (تُسمى "هدأة" أو remission)، حيث تختفي النوبات لشهور أو سنوات، خاصة مع التقدم في العمر أو تغييرات هرمونية (مثل بعد انقطاع الطمث عند النساء).	هل يمكن الشفاء من الشقيقة بشكل نهائي؟	24

Chatbot 3 (Grok 4.1)		
**العلاجات المتاحة حالياً (حتى 2026):**<br>• **علاج النوبات الحادة**: أدوية مثل التريبتانات، الجيبانت (مثل ubrogepant أو rimegepant)، أو بخاخات أنفية جديدة.<br>• **العلاج الوقائي**: أجسام مضادة لـCGRP (مثل erenumab أو fremanezumab)، أو أدوية أخرى مثل atogepant، والتي قللت النوبات بنسبة كبيرة لدى الكثيرين.<br>• **علاجات غير دوائية**: تغييرات في نمط الحياة (نوم منتظم، تجنب المحفزات مثل التوتر أو بعض الأطعمة)، أجهزة تحفيز عصبي، أو حتى جراحات في حالات نادرة مقاومة.<br><br>في بعض الحالات النادرة، قد تتحسن الحالة بشكل كبير مع الوقت أو العلاج، لدرجة أنها تبدو "شفيت"، لكن الاستعداد للنوبات قد يبقى.<br><br>إذا كنت تعاني من الشقيقة، استشر طبيباً متخصصاً في الصداع لخطة علاج شخصية، فقد تحسن حياتك بشكل ملحوظ. البحوث مستمرة، وهناك تقدم كبير في العلاجات الجديدة، لكن الشفاء النهائي لم يتحقق بعد.		
الشقيقة (أو الصداع النصفي، المعروف أيضًا باسم الميغرين) هي حالة عصبية مزمنة تسبب نوبات صداع شديدة، غالبًا مصحوبة بغثيان، قيء، حساسية للضوء والصوت، ويمكن أن تستمر من ساعات إلى أيام. تؤثر هذه الحالة بشكل كبير على حياة المصابين، حيث تقلل من جودة الحياة اليومية وتسبب عبئًا جسديًا، نفسيًا، اجتماعيًا، ومهنيًا.<br><br>**التأثير على الأنشطة اليومية والجسدية:**<br>• تعيق النوبات الأداء اليومي البسيط مثل الأعمال المنزلية، القيادة، أو حتى النوم، حيث يعاني أكثر من 50% من المصابين من اضطراب في النوم، وأكثر من 40% من تداخل في القيادة.<br>• حتى بين النوبات، يشعر المصابون بالقلق من تكرار الهجمات، مما يحد من تخطيط الأنشطة اليومية أو الترفيهية.<br><br>**التأثير على العمل والإنتاجية:**<br>• تُعد الشقيقة سببًا رئيسيًا لفقدان أيام العمل أو انخفاض الإنتاجية (الغياب عن العمل أو العمل بكفاءة منخفضة أثناء النوبة).<br>• يفقد المصابون حوالي 14-53% من إنتاجيتهم السنوية، مع تكاليف اقتصادية كبيرة للأفراد والمجتمع، مثل خسارة أيام عمل أو حتى تغيير الوظيفة أو الاستقالة بسبب عدم القدرة على التحمل.<br>• في بعض الحالات، يُجبر المصابون على العمل جزئيًا أو تغيير مسارهم المهني.<br><br>**التأثير على الحياة الاجتماعية والعائلية:**<br>• تمنع النوبات المشاركة في الأنشطة العائلية، الترفيهية، أو الاجتماعية، مما يؤدي إلى عزلة اجتماعية أو شعور بأن "الحياة تمر دون المشاركة فيها".<br>• يؤثر ذلك على العلاقات مع الزوج/الزوجة، الأطفال، أو الأصدقاء، حيث يفوت المصابون مناسبات مهمة أو يلغون خططًا بشكل متكرر.	كيف تؤثر الشقيقة على حياة المصابين؟	25

Chatbot 3 (Grok 4.1)		
		**التأثير على الصحة النفسية:**<br><br>● ترتبط الشقيقة بمعدلات أعلى من الاكتئاب (حوالي 28-50%)، القلق (22-60%)، والتوتر، بسبب الألم المزمن، الخوف من النوبة التالية، والشعور بالعجز.<br>● يمكن أن تؤدي إلى إرهاق نفسي، انخفاض الثقة بالنفس، أو حتى اضطرابات ما بعد الصدمة في بعض الحالات.<br><br>بشكل عام، تُصنف الشقيقة كواحدة من أكثر الأمراض إعاقةً للأشخاص دون سن 50 عامًا، وفقًا لمنظمة الصحة العالمية، حيث تقلل من جودة الحياة بشكل ملحوظ حتى في الأيام غير المصابة بنوبات. ومع ذلك، يمكن تحسين هذه التأثيرات من خلال العلاج المناسب، تجنب المحفزات، وتعديل نمط الحياة. إذا كنت تعاني منها، استشر طبيبًا متخصصًا للحصول على خطة علاجية فعالة.

Chatbot 4 (Deepseek V3.2)		
SN	السؤال	الرد
1	ما هي الشقيقة؟	الشقيقة (Migraine) هي اضطراب عصبي معقد، وليست مجرد صداع عادي. تُعد من أكثر أسباب الصداع شيوعًا وتتميز بنوبات متكررة من الصداع الشديد، غالبًا ما تصاحبها أعراض أخرى.<br><br>**الخصائص الرئيسية للشقيقة:**<br><br>1. طبيعة الصداع:<br>○ يكون عادةً نابضًا (يشعر النبض مع دقات القلب).<br>○ يصيب جانبًا واحدًا من الرأس في أغلب الحالات، لكنه قد ينتشر.<br>○ شدته متوسطة إلى شديدة، تمنع المصاب من ممارسة نشاطاته اليومية.<br>○ يزداد مع النشاط البدني البسيط (كالصعود على الدرج).<br>2. مدة النوبة:<br>○ تستمر من 4 ساعات إلى 3 أيام إذا لم تُعالج.<br>3. الأعراض المصاحبة (قد لا تظهر جميعها):<br>○ الغثيان أو القيء.<br>○ الحساسية الشديدة للضوء (رُهاب الضوء) والضوضاء (رُهاب الصوت)، وأحيانًا الروائ[illegible].

Chatbot 4 (Deepseek V3.2)

- الاضطرابات البصرية أو العصبية: تظهر عند حوالي 25% من المصابين قبل أو أثناء الصداع، وتسمى "أورة" (Aura)، وتشمل:
  - رؤية ومضات ضوئية، خطوط متعرجة، بقع سوداء.
  - تنميل أو وخز في الوجه أو اليدين.
  - صعوبة مؤقتة في الكلام (نادرًا).

4. النمط:
   - تأتي على شكل نوبات تفصلها فترات خالية تمامًا من الأعراض.

ما الذي يميز الشقيقة عن الصداع العادي؟

- الصداع التوتري (العادي): يكون أشبه بضغط أو شد حول الرأس، خفيف إلى متوسط الشدة، ولا يمنع النشاطات اليومية عادةً، ولا يصاحبه غثيان أو حساسية ضوئية شديدة.
- الشقيقة: كما وصفنا أعلاه، تكون أكثر حدة ومصحوبة بأعراض جهازية.

المحفزات الشائعة لنوبات الشقيقة:

تختلف من شخ[illegible] لآخر، وقد تشمل:

- عوامل غذائية (كالجبنة القديمة، الشوكولاتة، الكافيين، المواد الحافظة مثل الغلوتامات أحادية الصوديوم).
- الإجهاد أو الراحة بعد إجهاد.
- التغيرات الهرمونية (مثل فترة الدورة الشهرية لدى النساء).
- اضطرابات النوم (كثرة أو قلة النوم).
- المحفزات الحسية (الأضواء الساطعة، الروائ[illegible] القوية، الأصوات العالية).
- تغيرات الطقس أو الضغط الجوي.

العلاج والإدارة:

1. علاج النوبة الحادة: مسكنات خاصة مثل مجموعة التريبتان (Triptans)، مضادات الالتهاب غير الستيرويدية (مثل الإيبوبروفين أو النابروكسين)، وأدوية للغثيان.
2. العلاج الوقائي: يُعطى لمن يعانون من نوبات متكررة (أكثر من 4 نوبات شهريًا)، ويشمل أدوية مختلفة (حاصلات بيتا، مضادات الاكتئاب، مضادات الصرع، وأدوية حديثة مثل مضادات CGRP).
3. تعديل نمط الحياة: معرفة المحفزات وتجنبها، الانتظام في مواعيد النوم والوجبات،

<table>
<tr><th colspan="3">Chatbot 4 (Deepseek V3.2)</th></tr>
<tr><td>ممارسة الرياضة، وتقنيات إدارة الإجهاد (اليوجا، التأمل).

خلاصة:

الشقيقة هي مرض عصبي حقيقي له أساس جيني غالبًا، ويمكن أن يكون منهكًا جدًا ويؤثر على جودة الحياة. إذا كنت تعاني من أعراض تشبه الشقيقة، من المهم استشارة طبيب (طبيب أعصاب) لتشخي□ دقيق ووضع خطة علاج مناسبة. لا تستهن بالألم المتكرر، فالعلاجات الحديثة قدمت خيارات فعالة جدًا للتحكم بالنوبات.</td><td></td><td></td></tr>
<tr><td>الشقيقة (الصداع النصفي) هو اضطراب عصبي معقد له عدة أنواع فرعية، يمكن تصنيفها بشكل عام كما يلي:

١. حسب الأورة (الهالة) - التصنيف الأساسي

• الشقيقة مع الأورة (الهالة): يأتي الصداع مصحوبًا بأعراض عصبية بصرية أو حسية أو لغوية عابرة تسبق الصداع أو ترافقه (كالضوء الساطع، الخطوط المتعرجة، التنميل، ضعف الكلام). تدوم الأورة عادةً 5-60 دقيقة.
• الشقيقة دون أورة: هو النوع الأكثر شيوعًا، ويأتي الصداع دون علامات تحذيرية عصبية محددة.
• الشقيقة ذات الأورة النموذجية دون صداع: تظهر أعراض الأورة (كالبصرية) ولكن دون أن يتبعها صداع.

٢. حسب التردد والأعراض المميزة

• الشقيقة المزمنة: عندما يحدث الصداع لمدة 15 يومًا أو أكثر شهريًا (لمدة 3 أشهر على الأقل)، مع كون 8 أيام على الأقل منها صفات الشقيقة.
• الشقيقة الحميدة (النوبية): هي الشكل الشائع الذي يأتي على شكل نوبات متقطعة.
• الشقيقة الفالجية: نادرة، تتضمن ضعفًا مؤقتًا في أحد جانبي الجسم (شلل نصفي) كجزء من الأورة.
• شقيقة جذع الدماغ (الشقيقة القاعدية): تؤثر على جذع الدماغ، وتسبب أعراضًا مثل الدوخة، تداخل الكلام، طنين الأذن، أو فقدان التوازن قبل الصداع.</td><td>ما أنواع الشقيقة؟</td><td>2</td></tr>
</table>

Chatbot 4 (Deepseek V3.2)

- الشقيقة الشبكية (العينية): تسبب فقدانًا مؤقتًا للرؤية أو ظهور بقع عمياء في عين واحدة فقط قبل الصداع.

٣. حسب العوامل المحفزة أو الظروف

- شقيقة الدورة الشهرية: مرتبطة بتغيرات الهرمونات قبل أو خلال الدورة الشهرية.
- شقيقة الجهاز الهضمي (البطني): أكثر شيوعًا لدى الأطفال، مع أعراض مثل آلام البطن المتكررة، الغثيان، والقيء دون صداع شديد (قد يتطور لشقيقة نموذجية لاحقًا).
- شقيقة المراهقين أو الأطفال: قد تكون نوباتها أقصر وتصيب جانبي الرأس.

٤. حسب الاستجابة للعلاج أو المسار السريري

- الشقيقة المقاومة للعلاج: لا تستجيب للأدوية القياسية.
- حالة الشقيقة المستمرة: نوبة صداع نصفي شديدة تدوم أكثر من 72 ساعة رغم العلاج.

٥. أنواع نادرة أو خاصة

- متلازمة دورات الصداع النصفي: دورة من الأعراض مثل الصداع، القيء، ونوبات من الألم البطني.
- الشقيقة المصحوبة باختلال وظائف الجهاز العصبي اللاإرادي: مع أعراض بارزة مثل التعرق، احمرار الوجه، التدميع.

ملاحظة مهمة:

التشخي▯ الدقيق لنوع الشقيقة يجب أن يتم بواسطة طبيب أعصاب أو أخصائي، حيث أن الأعراض قد تتداخل مع حالات عصبية أخرى. العلاج يختلف حسب النوع، وقد يشمل أدوية للوقاية، أدوية للنوبة الحادة، وتغييرات في نمط الحياة.

إذا كنت تعاني من أعراض مشابهة، فاستشر الطبيب للحصول على تقييم شخصي وخطة علاج مناسبة.

<table>
<tr><th colspan="3">Chatbot 4 (Deepseek V3.2)</th></tr>
<tr>
<td>3</td>
<td>هل هناك فرق بين الشقيقة والصداع النصفي؟</td>
<td>
نعم، هناك فرق في الاستخدام، لكنهما يشيران إلى الحالة نفسها في الغالب.

الصداع النصفي (Migraine): هو المصطل□ الطبي العلمي المستخدم عالميًا لوصف هذا النوع المحدد من الصداع، الذي يتميز بنوبات متكررة من الألم الشديد النابض عادة في جانب واحد من الرأس (ولكن ليس دائمًا)، ويصاحبه غالبًا أعراض مثل الغثيان، القيء، والحساسية للضوء والصوت.
الشقيقة: هو الاسم العربي التقليدي أو الشائع لنفس الحالة (الصداع النصفي). أصل الكلمة من "الشِق" أي الجانب، مما يعكس سمة الألم في جانب واحد من الرأس التي يشتهر بها هذا الصداع.

خلاصة:
هما مصطلحان للمرض نفسه. "الصداع النصفي" هو الترجمة الحرفية والمصطل□ الطبي الأكثر استخدامًا في المجال الطبي الحديث، بينما "الشقيقة" هو الاسم التاريخي أو الشعبي العربي لنفس الحالة.
ملاحظة مهمة:
ليس كل صداع يحدث في جانب واحد من الرأس هو بالضرورة "صداع نصفي/شقيقة"، فهناك أنواع أخرى من الصداع قد تسبب ألمًا نصفيًا. التشخي □ الصحي□ يعتمد على مجموعة معايير تشمل خصائ □ الألم والأعراض المصاحبة ومدته، ويجب أن يتم من قبل طبيب مخت □.
</td>
</tr>
<tr>
<td>4</td>
<td>ما الفرق بين الشقيقة وصداع الجيوب الأنفية؟</td>
<td>
سؤال ممتاز، حيث يخلط الكثيرون بينهما بسبب تشابه بعض الأعراض. إليك الفروق الرئيسية بين الصداع النصفي (الشقيقة) و صداع الجيوب الأنفية في جدول توضيحي:
<table>
<tr><th>النقطة</th><th>الصداع النصفي (الشقيقة)</th><th>صداع الجيوب الأنفية</th></tr>
<tr><td>المسبب الرئيسي</td><td>اضطراب عصبي وعائي في الدماغ (خلل في الجهاز العصبي والأوعية</td><td>التهاب أو احتقان في الجيوب الأنفية (غالبًا</td></tr>
</table>
</td>
</tr>
</table>

Chatbot 4 (Deepseek V3.2)

	الدموية).	بسبب عدوى، حساسية، أو نزلة برد).
موقع الألم	عادةً في جانب واحد من الرأس (نصفي)، وقد ينتقل.	في مقدمة الوجه، الجبهة، الخدين، حول العينين، أو جسر الأنف (حيث توجد الجيوب).
طبيعة الألم	نابض أو خافق (كأن القلب ينبض في الرأس). غالبًا شديد إلى شديد جدًا.	ألم ثابت وضاغط أو إحساس بالامتلاء والثقل. متوسط إلى شديد.
الأعراض المصاحبة	الكلاسيكية: غثيان، قيء، حساسية شديدة للضوء والصوت والروائ[illegible]. قد يسبقه هالة: اضطرابات بصرية أو حسية.	مرتبطة بالجيوب: انسداد الأنف، إفرازات أنفية سميكة (صفراء/خضراء)، ضغط في الوجه، ألم يزداد عند الانحناء للأمام، قد يكون هناك حمى خفيفة.
مدة النوبة	من 4 ساعات إلى 72 ساعة إذا لم تعالج.	غالبًا يستمر طالما استمر التهاب الجيوب (أيام إلى أسابيع).

Chatbot 4 (Deepseek V3.2)

عدوى الجهاز التنفسي العلوي (البرد)، الحساسية الموسمية، التغير في ضغط الهواء (كالطيران).	الإجهاد، التغيرات الهرمونية، بعض الأطعمة، قلة النوم، الروائ□ القوية، التغيرات الجوية.	المحفزات
علاج السبب: مضادات الاحتقان، مضادات الهيستامين، غسول الأنف الملحي، المضادات الحيوية إذا كان هناك عدوى بكتيرية.	وقائي: أدوية محددة (حاصرات بيتا، إلخ)، تغيير نمط الحياة. للهجمة: مسكنات خاصة (التريبتانات)، مضادات الالتهاب.	العلاج

الخلاصة والتمييز العملي:

1. الأعراض العصبية والحسية: وجود الغثيان، القيء، أو الحساسية للضوء والصوت هو أقوى مؤشر على أنه صداع نصفي وليس صداع جيوب أنفية.
2. أعراض الجيوب الواضحة: وجود حمى، إفرازات أنفية قيحية، وألم يزداد مع الحركة المفاجئة للرأس يشير بقوة لالتهاب الجيوب.
3. تشخي□ خاطئ شائع: أظهرت الدراسات أن غالبية من يعتقدون أنهم يعانون من "صداع الجيوب الأنفية" يكون التشخي□ الصحي□ لهم هو الصداع النصفي، خاصة إذا كان الألم حول العينين والجبهة.
4. الاستجابة للعلاج: إذا تم علاج أعراض الاحتقان ولم يتحسن الصداع، أو إذا تحسن الصداع بأدوية الصداع النصفي، فمن المرج□ أن المشكلة هي شقيقة.

متى يجب زيارة الطبيب؟

- إذا كان الصداع شديدًا ومفاجئًا (مثل "رعدي").
- إذا صاحبه حمى عالية، تصلب في الرقبة، ارتباك، ضعف في الرؤية أو الكلام.

<table>
<tr><th colspan="3">Chatbot 4 (Deepseek V3.2)</th></tr>
<tr><td>

إذا كان هناك ضعف مفاجئ في طرف من الأطراف أو تنميل.
إذا كان الصداع يزداد سوءًا رغم العلاج.
إذا كان يمنعك من ممارسة حياتك الطبيعية بشكل متكرر.

الخطوة الأهم للحصول على التشخي الدقيق والعلاج المناسب هي استشارة طبيب (طبيب أعصاب للشقيقة، أو أخصائي أنف وأذن وحنجرة لالتهاب الجيوب).
</td><td></td><td></td></tr>
<tr><td>
صداع الشقيقة (الصداع النصفي) ليس عادةً مهدداً للحياة، لكنه حالة خطيرة من ناحية تأثيرها على جودة الحياة، ويمكن في بعض الحالات النادرة أن يؤدي إلى مضاعفات خطيرة.

إليك التفصيل:

١. من حيث الخطر المباشر على الحياة (نادر جداً)

نوبة الشقيقة الدماغية (Migrainous Infarction): هي أكثر المضاعفات خطورة، حيث يمكن أن تؤدي نوبة الشقيقة المصحوبة بهالة (أورة) طويلة ومستمرة إلى سكتة دماغية إقفارية (نق تروية). هذا نادر الحدوث، لكنه يعتبر حالة طبية طارئة.
متلازمة "السماح بالقتل" (Status Migrainosus): هي نوبة شقيقة شديدة تستمر لأكثر من ٧٢ ساعة دون توقف، مما يؤدي إلى الجفاف الشديد والإعاقة الكاملة. تتطلب عناية طبية عاجلة في المستشفى لكسر النوبة ومنع المضاعفات.

٢. من حيث الخطورة على الصحة وجودة الحياة (شائع ومهم)

هذا هو الجانب الأكثر شيوعاً وخطورة لمرض الشقيقة:

إعاقة شديدة: تُصنف منظمة الصحة العالمية الشقيقة من بين أكثر الأمراض إعاقةً للإنسان. خلال النوبة، يكون الشخ غير قادر على العمل، الدراسة، أو ممارسة الأنشطة اليومية.
تأثير نفسي واجتماعي: الألم المزمن والمفاجئ يؤدي إلى القلق، الاكتئاب، العزلة الاجتماعية، والتغيب المتكرر عن العمل أو المدرسة.
مشاكل دوائية: الإفراط في استخدام مسكنات الألم لعلاج النوبات (أكثر من ١٠-١٥ يومًا في الشهر) يمكن أن يؤدي إلى صداع الارتداد (الصداع الدوائي)، مما يفاقم المشكلة ويجعل الصداع يومياً تقريباً.
أمراض مصاحبة: توجد علاقة بين الشقيقة وزيادة خطر الإصابة ببعض الأمراض الأخرى مثل:
اضطرابات المزاب (الاكتئاب والقلق).

</td><td>هل صداع الشقيقة خطير؟</td><td>5</td></tr>
</table>

<table>
<tr><th colspan="3">Chatbot 4 (Deepseek V3.2)</th></tr>
<tr><td>

- اضطرابات النوم.
- أمراض القلب والأوعية الدموية (على المدى الطويل، وخاصة لدى المدخنات اللاتي يستخدمن حبوب منع الحمل الهرمونية).

متى يجب طلب العناية الطبية على الفور (علامات خطر)؟

اتصل بالطوارئ أو اذهب إلى المستشفى إذا صاحب الصداع أي من الأعراض التالية، خاصة إذا كانت جديدة أو مختلفة عن نوباتك المعتادة:

- صداع مفاجئ شديد جداً ("أسوأ صداع في حياتك").
- صداع مصحوب بـ حمى، تصلب الرقبة، تشوش ذهني، نوبات صرع، ازدواج الرؤية، أو ضعف/تنميل مفاجئ في أي جزء من الجسم.
- صداع بعد إصابة في الرأس.
- زيادة مفاجئة في وتيرة أو شدة الصداع.
- ظهور أعراض جديدة (خاصة إذا كنت فوق سن ٥٠).

الخلاصة:

الشقيقة مرض عصبي مزمن خطير من حيث تأثيره المدمر على حياة المريض اليومية ورفاهيته، ويحتاج إلى تشخي□ دقيق وخطة علاجية شاملة من طبيب متخص□ (عادةً طبيب أعصاب).

نصيحة: إذا كنت تعاني من صداع متكرر، لا تهمله ولا تعتمد فقط على المسكنات. راجع طبيباً للحصول على:
١. تشخي□ دقيق (للتأكد من أنه شقيقة وليس نوعاً آخر من الصداع).
٢. علاج وقائي لتقليل وتيرة وشدة النوبات.
٣. علاج abortive فعال وآمن لكسر النوبة عند بدايتها.
٤. خطة إدارة شاملة تشمل نمط الحياة (النوم، الطعام، الرياضة، إدارة التوتر).

</td><td></td><td></td></tr>
<tr><td>

أعراض الشقيقة ومراحل النوبة

أعراض الشقيقة الشائعة

</td><td>ما هي أعراض الشقيقة؟ وما هي المراحل التي قد تمر بها النوبة؟</td><td>6</td></tr>
</table>

<table>
<tr><th colspan="3">Chatbot 4 (Deepseek V3.2)</th></tr>
<tr><td>تختلف أعراض الشقيقة من شخ[illegible] لآخر، لكنها غالبًا تشمل:

الأعراض الأساسية:

• ألم نابض أو خافق في جانب واحد من الرأس (أو كليهما)
• حساسية شديدة للضوء والصوت والروائ[illegible]
• غثيان وقيء
• اضطرابات بصرية (هالات)
• دوخة ودوار

أعراض أخرى محتملة:

• تصلب الرقبة
• تغيرات في المزاج
• زيادة التبول
• صعوبة في التركيز والتحدث
• خدر أو تنميل في الوجه أو الأطراف

مراحل نوبة الشقيقة النموذجية

1. مرحلة البادرة (Prodrome)

• تبدأ قبل 24-48 ساعة من الصداع
• أعراض: تغيرات في المزاج، التعب، تصلب الرقبة، زيادة التبول، الرغبة الشديدة في أطعمة معينة</td><td></td><td></td></tr>
</table>

Chatbot 4 (Deepseek V3.2)

2. مرحلة الهالة (Aura)

- تحدث في حوالي 25% من المصابين
- تظهر قبل الصداع أو أثناءه
- أعراض بصرية: رؤية بقع، خطوط متعرجة، ومضات ضوئية
- أعراض حسية: تنميل أو وخز في الوجه أو الأطراف
- صعوبة في الكلام مؤقتة
- تستمر عادة 20-60 دقيقة

3. مرحلة الصداع (Headache)

- تستمر من 4 إلى 72 ساعة
- ألم نابض متوسط إلى شديد
- يزداد الألم مع النشاط البدني
- يرافقه غثيان وحساسية للمنبهات الحسية

4. مرحلة ما بعد الصداع (Postdrome)

- تستمر حتى 48 ساعة بعد زوال الألم
- يشعر الشخ □ بالإرهاق والضعف
- صعوبة في التركيز
- شعور عام بعدم الراحة

ملاحظة: لا يمر جميع المصابين بهذه المراحل الأربع كاملة، وقد تختلف التجربة من نوبة لأخرى ومن شخ □ لآخر.

إذا كنت تعاني من أعراض الشقيقة، فمن المهم استشارة طبيب لتشخي □ دقيق

Chatbot 4 (Deepseek V3.2)		
وخطة علاج مناسبة.		
"الهالة" في الصداع النصفي (الشقيقة) تشير إلى مجموعة من الاضطرابات الحسية أو العصبية التي تسبق نوبة الصداع النصفي في كثير من الأحيان (حوالي 25-30% من الحالات). الهالة هي عَرَض مميز للصداع النصفي مع هالة (Migraine with Aura).<br><br>**خصائص الهالة:**<br><br>1. توقيتها: تظهر الهالة عادة قبل بدء الصداع، وتستمر من 5 دقائق إلى 60 دقيقة، ونادراً ما تتجاوز الساعة.<br>2. طبيعتها: هي اضطرابات عصبية مؤقتة وقابلة للانعكاس، لا تسبب ضرراً دائماً للدماغ.<br><br>**أنواع وأعراض الهالة الشائعة:**<br><br>● هالة بصرية (الأكثر شيوعاً):<br>○ رؤية flashes من الضوء، نجوم، خطوط متعرجة أو متلألئة.<br>○ ظهور نقاط عمياء أو مناطق فقدان جزئي للرؤية (scotoma).<br>■ هالة حسية:<br>○ شعور بالوخز أو الخدر (تنميل) يبدأ غالباً في اليد، ثم ينتقل صعوداً إلى الذراع ثم الوجه، وخاصة حول الفم.<br>● هالة كلامية أو لفظية:<br>○ صعوبة مؤقتة في التحدث أو اختيار الكلمات، أو تشويش في الكلام.<br>● هالة حركية (نادرة):<br>○ ضعف أو شلل مؤقت في أحد أطراف الجسم أو جانب الوجه (تتطلب استبعاد أسباب خطيرة مثل السكتة الدماغية).<br><br>**آلية حدوث الهالة (النظرية العلمية السائدة):**<br><br>تُعرف بنظرية "الانتشار الاكتئابي القشري" (Cortical Spreading Depression - CSD)، وهي موجة من النشاط الكهربائي العصبي المفرط تليها حالة من الكبت/التثبيط، تنتشر ببطء عبر	ما هي الهالة (الأورة) في الشقيقة؟	7

<table>
<tr><th colspan="3">Chatbot 4 (Deepseek V3.2)</th></tr>
<tr><td>قشرة الدماغ (خاصة المنطقة البصرية). هذه الموجة تؤدي إلى:

• انخفاض مؤقت في تدفق الدم.
• تغيير في عمل الخلايا العصبية.
• ما يسبب الأعراض البصرية والحسية المرتبطة بالهالة.

أهمية التمييز:

من الضروري جداً التمييز بين هالة الصداع النصفي والأعراض العصبية المفاجئة التي قد تشير إلى حالات طارئة خطيرة، مثل السكتة الدماغية أو نوبات الصرع. يجب طلب الرعاية الطبية الفورية إذا:

• ظهرت الأعراض لأول مرة بعد سن الأربعين.
• كانت الهالة أطول من ساعة.
• كانت الأعراض مفاجئة وشديدة (صداع كصاعقة).
• صاحبها ضعف مفاجئ في الأطراف، أو تدلي في الوجه، أو اضطراب شديد في التوازن أو الكلام.
• حدثت الهالة دون أن يتبعها صداع.

الخلاصة: الهالة في الصداع النصفي هي علامة تحذيرية عصبية مؤقتة تسبق الصداع عادةً، وتنتج عن اضطراب كهروكيميائي في الدماغ. التشخي الدقيق من قبل الطبيب (غالباً طبيب الأعصاب) ووضع خطة علاج مناسبة أمران حاسمان لإدارة الحالة.</td><td></td><td></td></tr>
<tr><td>نعم، القيء والغثيان من الأعراض الشائعة جداً المصاحبة لنوبات الشقيقة (الصداع النصفي)، وخاصة الشقيقة مع الأورة أو بدون الأورة.

في الواقع، يعتبر الغثيان والقيء من المعايير التشخيصية للشقيقة وفقاً للتصنيف الدولي لاضطرابات الصداع. غالباً ما يكون الغثيان أحد أكثر الأعراض إزعاجاً للمريض، ويمكن أن يكون القيء في بعض الأحيان شديداً لدرجة أن المريض لا</td><td>هل القيء والغثيان من أعراض الشقيقة؟</td><td>8</td></tr>
</table>

Chatbot 4 (Deepseek V3.2)

يستطيع تناول الطعام أو الدواء.

تفصيل أكثر:

1. نسبة الحدوث: يعاني ما يصل إلى 80% من مرضى الشقيقة من الغثيان، ويعاني حوالي 50% منهم من القيء خلال النوبات.
2. التوقيت: قد يظهر الغثيان والقيء قبل بدء الصداع، أو أثنائه، أو حتى بعده.
3. الأعراض المرافقة الشائعة: بالإضافة إلى الصداع الشديد النابض (غالباً في جانب واحد من الرأس)، قد تشمل النوبة أعراضاً أخرى مثل:
   - الحساسية المفرطة للضوء (رهاب الضوء).
   - الحساسية المفرطة للصوت (رهاب الصوت).
   - الحساسية المفرطة للروائ□ (رهاب الروائ□).
   - الدوخة.
   - اضطرابات بصرية أو حسية في حالة الشقيقة المصحوبة بأورة (مثل رؤية الأضواء الوامضة أو الشعور بتنميل في الوجه أو اليدين).
4. تأثير على الحياة: يمكن أن يؤدي الغثيان والقيء إلى تفاقم معاناة المريض، حيث يمنعانه من تناول أدوية علاج النوبة (مثل المسكنات أو التريبتانات) أو يقلل من امتصاصها وفعاليتها إذا تقيأ بعد تناولها.

متى يجب استشارة الطبيب بشكل عاجل؟

إذا كانت هذه هي المرة الأولى التي تصاب فيها بنوبة صداع شديدة مصحوبة

بقيء، أو إذا ترافق القيء مع أعراض عصبية خطيرة مثل:

- ضعف مفاجئ في أحد جانبي الجسم.
- صعوبة في الكلام أو الفهم.
- تشوش في الرؤية.
- تيبس في الرقبة مع حرارة عالية.
- فقدان للوعي.

Chatbot 4 (Deepseek V3.2)

		• أو إذا كان القيء عنيفاً ومستمراً دون توقف.<br><br>في هذه الحالات، يجب استبعاد أسباب أخرى أكثر خطورة مثل النزف الدماغي أو التهاب السحايا.<br><br>الخلاصة: القيء والغثيان أعراض كلاسيكية للشقيقة، وغالباً ما تكون جزءاً لا يتجزأ من النوبة. إذا كنت تعاني منها بشكل متكرر، فمن المهم مراجعة طبيب أعصاب لتشخي [illegible] الحالة بدقة ووضع خطة علاجية مناسبة قد تشمل أدوية للوقاية من النوبات وأخرى لعلاجها عند الحدوث، بما في ذلك أدوية للتحكم في الغثيان والقيء.
9	هل طنين الأذن أو آلام الرقبة لها علاقة بالشقيقة؟	نعم، كل من طنين الأذن وآلام الرقبة يمكن أن يكون لهما علاقة بالشقيقة (الصداع النصفي)، وهذه العلاقة معروفة في الأدبيات الطبية. إليك التفاصيل:<br><br>**1. طنين الأذن والصداع النصفي:**<br><br>• الصداع النصفي المصحوب بأورة (Migraine with Aura): في بعض الحالات، قد يظهر طنين الأذن كجزء من "الأورة" التي تسبق نوبة الصداع النصفي. الأورة هي أعراض عصبية مؤقتة (مثل اضطرابات بصرية، تنميل، أو طنين) تحدث قبل أو أثناء الصداع.<br>• الصداع النصفي الدهليزي: هناك نوع فرعي من الصداع النصفي يرتبط بأعراض دهليزية مثل الدوخة، عدم التوازن، وطنين الأذن، حتى دون صداع شديد في بعض الأحيان.<br>• آلية محتملة: يُعتقد أن التغيرات في نشاط الدماغ والأوعية الدموية خلال نوبة الصداع النصفي قد تؤثر على الجهاز السمعي، مما يؤدي إلى طنين مؤقت أو مزمن لدى بعض المرضى.<br><br>**2. آلام الرقبة والصداع النصفي:**<br><br>• الصداع النصفي العنقي: كثيراً ما يُبلغ مرضى الصداع النصفي عن آلام في الرقبة قبل أو أثناء أو بعد النوبة. في بعض الحالات، قد يكون ألم الرقبة جزءاً من "الأورة" أو العلامة

Chatbot 4 (Deepseek V3.2)		
		الأولى لنوبة الصداع.<br>• التشابك العصبي: هناك تداخل بين الأعصاب التي تعصب منطقة الرقبة وتلك المسؤولة عن نقل أحاسيس الصداع النصفي في الدماغ (مثل العصب ثلاثي التوائم). هذا قد يسبب إحالة الألم بين المنطقتين.<br>• توتر العضلات: نوبات الصداع النصفي قد تسبب توتراً عضلياً في الرقبة والكتفين، مما يؤدي إلى ألم ثانوي. والعكس صحي□: مشاكل الرقبة (مثل اعتلال الفقرات العنقية) قد تحفز أو تفاقم نوبات الصداع النصفي لدى بعض الأشخاص.<br><br>**3. تشخيص متلازمات مترابطة:**<br><br>• بعض الحالات مثل متلازمة الصداع النصفي العنقي (Cervicogenic Migraine) تعتبر فيها مشاكل الرقبة محفزاً رئيسياً للصداع النصفي.<br>• طنين الأذن المرتبط بالصداع النصفي قد يتم تشخيصه عندما يتزامن الطنين مع النوبات، خاصة إذا استبعدت أسباب أخرى للأذن.<br><br>**4. التوصيات:**<br><br>• إذا كنت تعاني من هذه الأعراض، فمن المهم استشارة طبيب أعصاب أو أخصائي آلام رأس وعنق لتقييم حالتك بدقة.<br>• قد يشمل العلاج أدوية الصداع النصفي الوقائية (مثل حاصلات بيتا، مضادات الاكتئاب)، بالإضافة إلى علاجات مساعدة مثل العلاج الطبيعي للرقبة، وإدارة التوتر، وتعديل نمط الحياة.<br>• قد يساعد تدوين مذكرات الصداع لتتبع العلاقة بين الأعراض في تحديد المحفزات والأنماط.<br><br>ملاحظة مهمة: يجب استبعاد الأسباب الأخرى لطنين الأذن (مثل مشاكل الأذن الداخلية، التعرض للضوضاء) وآلام الرقبة (مثل الإصابات، الانزلاق الغضروفي) عبر الفح □ الطبي قبل اعتبارها مرتبطة بالشقيقة فقط.<br><br>باختصار، العلاقة موجودة وعلمياً معترف بها، وتشير إلى الطبيعة المعقدة للصداع النصفي الذي يؤثر على عدة أنظمة في الجسم. التشخي □ الدقيق يساعد في توجيه العلاج المناسب.
10	ما أسباب الشقيقة؟ وما هي محفزات النوبات؟	الشقيقة (الصداع النصفي) هي حالة عصبية معقدة، والأسباب الدقيقة للإصابة بها غير مفهومة تماماً، لكن يُعتقد أنها ناتجة عن مزيج من العوامل الوراثية والبيئية التي تؤثر على نشاط الدماغ والأعصاب والأوعية الدموية.

Chatbot 4 (Deepseek V3.2)

الأسباب الرئيسية المحتملة:

1. العوامل الوراثية: تلعب الوراثة دوراً كبيراً. فإذا كان أحد الوالدين يعاني من الشقيقة، تزداد احتمالية إصابة الأبناء بها.
2. نشاط الدماغ غير الطبيعي: يُعتقد أن التغيرات في جذع الدماغ وتفاعله مع العصب الثلاثي التوائم (المسؤول عن الإحساس في الوجه) يؤدي إلى إطلاق مواد كيميائية (مثل السيروتونين) تسبب التهاب الأوعية الدموية في أغشية الدماغ، مما يؤدي إلى الألم.
3. اختلال توازن النواقل العصبية: التغيرات في مستويات بعض المواد الكيميائية في الدماغ، وخاصة السيروتونين، تُعد من المحركات الأساسية.

محفزات نوبات الشقيقة الشائعة:

يمكن أن تختلف المحفزات بشكل كبير من شخ لآخر، وأهمها:

1. المحفزات الغذائية:

- تخطي وجبات الطعام أو الجفاف.
- بعض الأطعمة: الجبن القديم، الشوكولاتة، المواد الحافظة مثل (الغلوتامات أحادية الصوديوم) في الأطعمة المصنعة، والأسبرتام.
- المشروبات: الكحول (خاصة النبيذ الأحمر)، والإفراط في الكافيين أو الانسحاب منه.

2. المحفزات الحسية:

- الأضواء الساطعة أو الوامضة (مثل شاشات الكمبيوتر).
- الأصوات العالية أو الضجيج المستمر.
- الروائ القوية (العطور، الدخان، المواد الكيميائية).

3. المحفزات النفسية والهرمونية:

- التوتر والإجهاد: أو حتى فترة الاسترخاء بعد التوتر ("صداع نهاية الأسبوع").
- التغيرات الهرمونية لدى النساء: تقلبات هرمون الاستروجين قبل أو خلال الدورة الشهرية، أو أثناء الحمل أو انقطاع الطمث.
- قلة النوم أو الإفراط فيه، واضطرابات النوم.

4. المحفزات الجسدية والبيئية:

- الإرهاق الجسدي الشديد.

Chatbot 4 (Deepseek V3.2)		
• تغيرات الطقس والضغط الجوي.<br>• التدخين أو الجلوس في أماكن مغلقة مليئة بالدخان.<br>• بعض الأدوية: مثل حبوب منع الحمل، وأدوية علاج ضغط الدم.<br><br>**نصائح مهمة:**<br><br>• التتبع: أفضل طريقة لفهم محفزاتك الشخصية هي الاحتفاظ بمذكرة للصداع تدون فيها وقت النوبة، ومدتها، وشدتها، وما أكلته، ونوعية نومك، وأي أحداث مررت بها قبلها.<br>• الاستشارة الطبية: التشخي □ الصحي□ من قبل طبيب (عادة طبيب أعصاب) ضروري لاستبعاد أسباب أخرى وللوصول للعلاج المناسب الذي قد يشمل أدوية للوقاية وأخرى لعلاج النوبة الحادة.<br>• نمط الحياة: يمكن أن يساعد الحفاظ على روتين منتظم في النوم والوجبات، وشرب كمية كافية من الماء، وممارسة الرياضة بانتظام، وتقنيات إدارة التوتر (مثل التأمل)، في تقليل وتيرة النوبات.<br><br>تذكر أن هذه المعلومات هي للتعليم العام، ولا تغني عن استشارة الطبيب المخت □ لتشخي □ حالتك ووضع خطة علاجية مناسبة لك.		
نعم، الشقيقة (الصداع النصفي) لديها أساس وراثي قوي، لكنها لا تنتقل بنمط وراثي بسيط. يمكننا التفصيل كالتالي:<br><br>**الجانب الوراثي:**<br><br>• ليست مرضًا وراثيًا بالمعنى الكلاسيكي (مثل بعض الأمراض التي تنتقل بجين واحد)، ولكنها حالة ذات استعداد وراثي.<br>• إذا كان أحد الوالدين مصابًا بالشقيقة، تزداد احتمالية إصابة الأبناء بـ 30-40%.<br>• إذا كان كلا الوالدين مصابين، تزداد النسبة إلى 50-75%.<br>• يُعتقد أن مجموعة من الجينات (متعددة الجينات) تتفاعل مع العوامل البيئية لإحداث النوبة (نموذج "الجينات + المحفزات").<br><br>**الفئات الأكثر عرضة للإصابة:**<br><br>1. الجنس: النساء أكثر إصابة من الرجال بـ 3 أضعاف (بسبب التقلبات الهرمونية، خاصة ارتباط النوبات بدورة الطمث).<br>2. العمر: غالبًا تبدأ في مرحلة المراهقة والشباب (بين 18-44 سنة)، وقد تتحسن بعد سن اليأس لدى النساء.	هل الشقيقة مرض وراثي ومن هم الأكثر عرضة للإصابة؟	11

<table>
<tr><th colspan="3">Chatbot 4 (Deepseek V3.2)</th></tr>
<tr><td></td><td></td><td>3. التاريخ العائلي: وجود قريب من الدرجة الأولى (أب، أم، أخ، أخت) مصاب يزيد المخاطر.
4. الحالات الطبية: المصابون بالاكتئاب، القلق، الصرع، متلازمة القولون العصبي، وبعض أمراض الأوعية الدموية.
5. العوامل الهرمونية: النساء بشكل عام، خاصة أثناء التغيرات الهرمونية كالدورة الشهرية، الحمل، أو انقطاع الطمث.
6. نمط الحياة والبيئة:
○ التعرض المتكرر للضغوط النفسية.
○ اضطرابات النوم (قلة أو كثرة النوم).
○ بعض الأطعمة والمشروبات (كالجبن المعتق، النبيذ الأحمر، المحليات الصناعية).
○ التغيرات المناخية والروائ□ القوية.
○ الإجهاد البدني أو العقلي المفرط.

ملاحظة مهمة:

الاستعداد الوراثي لا يعني حتمية الإصابة، ولكن وجوده يجعل الشخ □ أكثر تفاعلًا مع المحفزات البيئية والسلوكية. لذا، تجنب هذه المحفزات يمكن أن يقلل بشكل كبير من تواتر وشدة النوبات حتى لدى من لديهم استعداد وراثي.

نصيحة: إذا كنت تعاني من أعراض الصداع النصفي المتكررة، فمن المهم استشارة طبيب أعصاب لتشخي □ دقيق وخطة علاجية مناسبة تتناسب مع حالتك.</td></tr>
<tr><td>12</td><td>ما الأطعمة التي قد تحفز نوبات الشقيقة؟</td><td>نعم، هناك أطعمة ومشروبات معروفة بأنها قد تحفز نوبات الشقيقة (الصداع النصفي) لدى بعض الأشخاص. من المهم ملاحظة أن المحفزات تختلف من شخ □ لآخر، وليس بالضرورة أن تسبب هذه الأطعمة نوبة لدى كل مصاب.

المحفزات الغذائية الشائعة تشمل:

1. الأطعمة والمشروبات المحتوية على "التيرامين": وهي مادة طبيعية تتكون عند تخمر أو تقدّم عمر الأطعمة.
○ الأجبان المعتقة: مثل الجبنة الزرقاء، الشيدر، الجودة، البارميزان، والفيتا.
○ اللحوم المصنعة والمقددة: مثل السلامي، النقانق، الهوت دوغ، واللحم المقدد (بيكون) (غالباً بسبب النترات والنيتريت أيضاً).
○ الأطعمة المخمرة أو المخللة: مثل مخلل الملفوف، صلصة الصويا، والتوفو المخمر.</td></tr>
</table>

Chatbot 4 (Deepseek V3.2)

2. الكافيين: يمكن أن يكون محفزاً أو مسكناً للصداع حسب الكمية والفرد.
   - الإفراط في تناول القهوة، الشاي، المشروبات الغازية، ومشروبات الطاقة.
   - انسحاب الكافيين (عدم الحصول على الجرعة المعتادة) هو محفز شائع جداً.
3. الكحول: خاصة النبيذ الأحمر، نظراً لاحتوائه على التيرامين والتانينات والكبريتيت. كما أن البيرة والمشروبات الكحولية الأخرى قد تكون محفزة.
4. المحليات الصناعية: أشهرها الأسبارتام، الموجود في العديد من المشروبات الغازية "الدايت" والحلويات الخالية من السكر.
5. الغلوتامات أحادية الصوديوم (MSG): وهي محسن للنكهة يستخدم في المطاعم (خاصة الصينية) والأطعمة المصنعة، والوجبات السريعة، والمكعبات الجاهزة.
6. الأطعمة الباردة جداً: مثل المثلجات أو المشروبات المثلجة، خاصة إذا تم تناولها بسرعة بعد مجهود أو في يوم حار.
7. بعض الفواكه والمكسرات:
   - الحمضيات (كالبرتقال والليمون والجريب فروت) لدى بعض الأشخاص.
   - الفول السوداني وزبدة الفول السوداني.
   - الأفوكادو.
   - البصل (أقل شيوعاً).
8. الشوكولاتة: وهو من أكثر المحفزات المذكورة، رغم أن بعض الدراسات تشير إلى أن الرغبة الشديدة في تناول الشوكولاتة قد تكون جزءاً من أعراض "البادرة" التي تسبق النوبة وليس المحفز بحد ذاته.

نصائ□ مهمة لإدارة المحفزات الغذائية:

1. حافظ على مذكرة للصداع: سجّل الأطعمة التي تناولتها وتوقيت نوبات الشقيقة. هذا هو أفضل طريقة لتحديد المحفزات الشخصية لك بدقة، بدلاً من تجنب جميع الأطعمة المذكورة.
2. انتظام الوجبات: تخطي الوجبات أو الجوع هو محفز قوي للشقيقة لدى الكثيرين، وأحياناً أقوى من الطعام نفسه. حافظ على انتظام مواعيد الوجبات وتناول وجبات متوازنة.
3. ترطيب الجسم: الجفاف محفز رئيسي. اشرب كميات كافية من الماء على مدار اليوم.
4. التدريج في التجنب: إذا لاحظت أن نوعاً معيناً من الطعام يسبب النوبة بشكل متكرر، حاول تجنبه لمدة شهر ولاحظ إذا كان هناك تحسن. لا تبدأ بتجنب قوائم طويلة من الأطعمة مرة واحدة.
5. استشر طبيباً مختصاً: استشر طبيب الأعصاب أو أخصائي التغذية. يمكنهم مساعدتك في وضع خطة غذائية متوازنة وتقديم التشخي □ الدقيق والعلاج المناسب لحالتك.

تذكر أن التوتر، قلة النوم، التغيرات الهرمونية، الروائ□ القوية، والأضواء الساطعة هي أيضاً محفزات شائعة جداً وقد تكون أكثر تأثيراً من الغذاء لدى الكثيرين. الإدارة الشاملة لنمط الحياة هي المفتاح.

Chatbot 4 (Deepseek V3.2)		
تشخي □ الشقيقة (الصداع النصفي) يعتمد بشكل أساسي على التاريخ الطبي التفصيلي والفح □ السريري، ولا يوجد فح □ مخبري أو تصويري وحيد يؤكد التشخي □. إليك الخطوات الرئيسية التي يتبعها الأطباء:<br><br>**1. التاريخ الطبي التفصيلي (الجزء الأهم)**<br><br>يسأل الطبيب عن طبيعة الألم والأعراض المصاحبة، بما في ذلك:<br><br>● موقع وطبيعة الألم: هل هو في جانب واحد من الرأس؟ هل هو نابض؟<br>● شدة ومدة النوبة: من ساعات إلى 3 أيام عادة.<br>● الأعراض المصاحبة:<br>○ الغثيان والقيء.<br>○ الحساسية للضوء أو الصوت (وربما الروائ□).<br>● الأعراض البادرية (إن وجدت):<br>○ أورة (Aura): أعراض عصبية تسبق الصداع (تستمر 5-60 دقيقة)، مثل:<br>■ اضطرابات بصرية (رؤية أضواء وامضة، خطوط متعرجة، بقع عمياء).<br>■ تنميل أو ضعف في جانب واحد من الجسم.<br>■ صعوبة في الكلام.<br>● مُحفّزات الصداع: مثل التوتر، قلة النوم، بعض الأطعمة، التغيرات الهرمونية لدى النساء.<br>● تاريخ عائلي: وجود تاريخ عائلي للشقيقة يزيد من الاحتمال.<br><br>**2. الفحص السريري والعصبي**<br><br>يقوم الطبيب بإجراء فح □ عام وعصبي كامل لاستبعاد أسباب أخرى للصداع،	كيف يتم تشخيص الشقيقة؟	13

Chatbot 4 (Deepseek V3.2)

وفح □:

- الرؤية وحركة العينين.
- ردود الفعل.
- التناسق العضلي.
- الإحساس.

3. معايير التشخيص (وفقًا للتصنيف الدولي لاضطرابات الصداع - 3-ICHD)

يشخّ □ الطبيب الشقيقة إذا توافقت الأعراض مع المعايير، مثل:

الشقيقة دون أورة:

- 5 نوبات على الأقل تستمر 4-72 ساعة (بدون علاج).
- اثنان على الأقل من الخصائ □ التالية:
  1. ألم في جانب واحد من الرأس.
  2. ألم نابض.
  3. ألم متوسط إلى شديد الشدة.
  4. يتفاقم بالحركة أو النشاط البدني الروتيني.
- عرض واحد على الأقل من:
  1. غثيان و/أو قيء.
  2. حساسية للضوء والصوت.

الشقيقة مع أورة:

- نوبتان على الأقل من الأورة العابرة المصحوبة بالصداع.

Chatbot 4 (Deepseek V3.2)

4. الفحوصات المساعدة (لاستبعاد أسباب أخرى)

لا تُستخدم لتأكيد الشقيقة، ولكن إذا كانت الأعراض غير نمطية أو هناك علامات خطيرة، قد يطلب الطبيب:

- التصوير بالرنين المغناطيسي (MRI) للدماغ.
- التصوير المقطعي المحوسب (CT Scan).
- فحوصات دم لاستبعاد عدوى أو اضطرابات أخرى.

5. مذكرة يومية للصداع

قد يطلب منك الطبيب تسجيل تفاصيل النوبات لعدة أسابيع، تشمل:

- توقيت وطول النوبة.
- الأعراض والشدة.
- المحفزات المحتملة.
- الأدوية المستخدمة واستجابتها.

علامات الخطر التي تستدعي فحوصات إضافية

إذا كان الصداع مصحوبًا بأي مما يلي، يجب البحث عن أسباب ثانوية:

- بداية مفاجئة لصداع شديد للغاية (صداع الرعد).
- ظهور صداع جديد بعد سن 50.
- تغير في نمط الصداع.
- أعراض عصبية مستمرة (ضعف، تشوش ذهني، نوبات صرعية).

Chatbot 4 (Deepseek V3.2)		
• صداع يتبع إصابة في الرأس.<br>• وجود حمى، تصلب الرقبة، أو طف□ جلدي.<br><br>**من الذي يشخص الشقيقة؟**<br><br>• طبيب الأسرة أو الباطنية.<br>• طبيب الأعصاب (خاصة في الحالات المعقدة).<br><br>الهدف من التشخي □ الدقيق هو وضع خطة علاج مناسبة تشمل:<br><br>1. علاج النوبة الحادة (مسكنات، أدوية محددة للتريبتانات).<br>2. العلاج الوقائي (إذا كانت النوبات متكررة أو شديدة).<br>3. إدارة المحفزات ونمط الحياة.<br><br>إذا كنت تعاني من أعراض مشابهة، راجع طبيبًا للحصول على تشخي □ دقيق وخطة علاج شخصية.		
الصداع النصفي (الشقيقة) هو مرض عصبي شائع جدًا على مستوى العالم. إليك نظرة على مدى شيوعها:<br><br>**على المستوى العالمي:**<br><br>• الانتشار العالمي: وفقًا لمنظمة الصحة العالمية (WHO)، يُصاب حوالي 1 من كل 7 أشخاص بالشقيقة، مما يجعلها ثالث أكثر الأمراض انتشارًا في العالم.<br>• الأرقام: يُقدّر أن حوالي 1 مليار شخ □ حول العالم يعانون منها.	ما مدى شيوع الشقيقة؟	14

Chatbot 4 (Deepseek V3.2)

- العبء العالمي: تحتل الشقيقة المرتبة الثانية بين أسباب سنوات العجز عالميًا (بعد آلام الظهر)، مما يبرز تأثيرها الكبير على جودة الحياة والإنتاجية.

حسب الجنس:

- النساء أكثر عرضة للإصابة بها بثلاثة أضعاف مقارنة بالرجال، وذلك بشكل رئيسي بسبب العوامل الهرمونية (مثل التقلبات المرتبطة بالدورة الشهرية، الحمل، وانقطاع الطمث).
- التقديرات: تصيب حوالي 18-25% من النساء، وحوالي 6-10% من الرجال.

حسب العمر:

- غالبًا ما تبدأ في سن البلوغ (أو قبلها في بعض الحالات).
- تكون أكثر نشاطًا وعنفًا في الفترة العمرية بين 35 و45 سنة.
- قد تتحسن وتقل وتيرتها بعد سن اليأس لدى العديد من النساء.

في العالم العربي والشرق الأوسط:

- النسب مشابهة للنسب العالمية تقريبًا، مع بعض الاختلافات الطفيفة بين الدول.
- أشارت دراسات في بعض دول المنطقة (مثل السعودية، الأردن، عُمان) إلى انتشار يتراوح بين 10% إلى 15% عمومًا، مع ارتفاع النسبة بين النساء.
- غالبًا ما يكون هناك نق▯ في التشخي▯ بسبب قلة الوعي أو اعتبارها مجرد "صداع عادي"، مما يعني أن الأرقام الفعلية قد تكون أعلى.

<table>
<tr><th colspan="3">Chatbot 4 (Deepseek V3.2)</th></tr>
<tr><td>

عوامل مهمة مرتبطة بالشيوع:

1. الوراثة: للتاريخ العائلي دور كبير (إذا كان أحد الوالدين مصابًا، تزداد احتمالية الإصابة).
2. الحالة الاجتماعية والاقتصادية: تؤثر على إمكانية الوصول للتشخي□ والعلاج المناسبين.
3. نمط الحياة: الإجهاد، اضطرابات النوم، بعض الأطعمة يمكن أن تكون محفزات.

الخلاصة:

الشقيقة ليست حالة نادرة أبدًا، بل هي حالة شائعة جدًا تؤثر على مئات الملايين بشكل منتظم، وتسبب ألمًا شديدًا وعجزًا مؤقتًا للمصابين. زيادة الوعي بها وفهمها كمرض عصبي حقيقي وليس مجرد "صداع بسيط" هو أمر بالغ الأهمية للتعامل المناسب معها وتقليل عبئها على الأفراد والمجتمعات.

إذا كنت تعاني أنت أو أحد معارفك من أعراض تشبه الشقيقة (صداع نابض في جانب واحد، حساسية للضوء والصوت، غثيان، إلخ)، فمن المهم استشارة طبيب أعصاب أو طبيب رعاية أولية للحصول على تشخي□ دقيق وخطة علاج مناسبة.

</td><td></td><td></td></tr>
<tr><td>نوبات الشقيقة (الصداع النصفي) يمكن أن تبدأ في أي عمر، لكن هناك أنماط عمرية شائعة:</td><td>في أي عمر تبدأ نوبات الشقيقة؟</td><td>15</td></tr>
</table>

#		Chatbot 4 (Deepseek V3.2)
		الفترات الأكثر شيوعاً للبداية:<br><br>• المراهقة والبلوغ المبكر: تُعد الفترة الأكثر شيوعاً لبداية نوبات الشقيقة، خاصة مع التغيرات الهرمونية.<br>• مرحلة الطفولة: يمكن أن تبدأ في عمر مبكر، حتى في الأطفال قبل سن المدرسة، وقد تظهر بأعراض مختلفة مثل آلام البطن المتكررة أو الدوار.<br>• سن البلوغ: غالباً ما تبلغ ذروتها بين سن 25 و55 عاماً.<br><br>ملاحظات مهمة:<br><br>1. الاختلاف حسب الجنس: تكون الشقيقة أكثر شيوعاً لدى النساء (تبدأ غالباً مع بداية الدورة الشهرية أو التغيرات الهرمونية).<br>2. العوامل الوراثية: وجود تاريخ عائلي يزيد من احتمالية البداية في عمر مبكر.<br>3. أعراض الطفولة: قد لا تظهر كصداع شديد، بل كدوار، ألم بطني متكرر، أو "غثيان السيارة".<br><br>نصيحة: إذا ظهرت أعراض تشبه الشقيقة في أي عمر (حتى لدى الأطفال)، يُنص[illegible] باستشارة طبيب أعصاب أو طبيب أسرة للتشخي [illegible] الدقيق ووضع خطة علاج مناسبة، حيث أن التشخي [illegible] والعلاج المبكرين يساعدان في تحسين جودة الحياة بشكل كبير.
16	هل يمكن أن يصاب الأطفال بالشقيقة؟	نعم، يمكن أن يُصاب الأطفال بالشقيقة (الصداع النصفي). في الواقع، الشقيقة هي حالة عصبية شائعة نسبيًا في مرحلة الطفولة والمراهقة.<br><br>بعض النقاط المهمة حول الشقيقة عند الأطفال:<br><br>1. الانتشار: يُصاب حوالي 3-10% من الأطفال بالشقيقة. قد تبدأ في سن مبكرة، حتى في مرحلة ما قبل المدرسة، وتزداد معدلاتها مع التقدم في العمر، خاصة خلال فترة البلوغ.<br>2. اختلاف الأعراض: قد تختلف أعراض الشقيقة لدى الأطفال عن البالغين في بعض

<table>
<tr><th colspan="3">Chatbot 4 (Deepseek V3.2)</th></tr>
<tr><td></td><td></td><td>النواحي:
○ مدة النوبة: غالبًا ما تكون أقصر، فقد تستمر من ساعة إلى 72 ساعة.
○ موقع الألم: قد يكون الألم في جانبي الرأس (ثنائي الجانب) بدلاً من جانب واحد، خاصة عند الأطفال الصغار.
○ أعراض بارزة: قد تكون الغثيان والقيء أو الدوار أكثر وضوحًا من الصداع نفسه. قد يظهر الطفل شاحبًا، خاملاً، أو يتجنب الضوء والضجة.
○ الأورة: قد تحدث "الأورة" (أعراض حسية أو بصرية قبل النوبة) ولكن يصعب على الطفل الصغير وصفها.
3. الأشكال الشائعة في الطفولة: هناك أنواع خاصة من الشقيقة أو المتلازمات المرتبطة بها التي تظهر في الطفولة، مثل:
○ القيء الدوري: نوبات متكررة من القيء الشديد.
○ الدوار الحميد الطفولي: نوبات مفاجئة من الدوار وفقدان التوازن تستمر دقائق ثم تزول.
○ الآلام البطنية النصفية: ألم متكرر في البطن مع غثيان أو قيء دون صداع بالضرورة.
4. المحفزات: تشبه محفزات الشقيقة لدى الأطفال تلك التي لدى البالغين، وتشمل: قلة النوم، الجفاف، تخطي الوجبات، التوتر، بعض الأطعمة (مثل الجبن والشوكولاتة والمواد الحافظة)، الضوء الساطع، والضجيج.
5. التشخي ⍰ والعلاج: يعتمد التشخي ⍰ بشكل رئيسي على التاريخ الطبي التفصيلي وفح ⍰ الطفل العصبي. العلاج يشمل:
○ تجنب المحفزات: تنظيم النوم والوجبات وشرب الماء.
○ العلاج الحاد: أدوية مسكنة ومضادة للغثيان عند بدء النوبة (يجب وصفها من قبل الطبيب).
○ العلاج الوقائي: في الحالات المتكررة أو الشديدة، قد يصف الطبيب أدوية أو مكملات غذائية (مثل المغنيسيوم) للوقاية.
○ العلاج غير الدوائي: تعليم الطفل تقنيات الاسترخاء والتحكم في التوتر.

متى يجب استشارة الطبيب؟
يجب استشارة طبيب الأطفال أو طبيب الأعصاب للأطفال إذا كان الطفل يشكو من صداع متكرر، خاصة إذا كان:

• يوقظه من النوم.
• يزداد سوءًا أو يصاحبه قيء.
• يؤثر على أدائه المدرسي أو نشاطاته اليومية.
• يتغير نمطه أو طبيعته فجأة.

الخبر السار هو أن العديد من الأطفال تتحسن حالتهم مع التقدم في العمر، ويمكن السيطرة على النوبات بشكل فعال بمساعدة طبية مناسبة.</td></tr>
<tr><td>17</td><td>هل الشقيقة مرض مزمن؟</td><td>نعم، الصداع النصفي (الشقيقة) يعتبر عادةً مرضًا مزمنًا، لكن هذه الإجابة تحتاج إلى بعض</td></tr>
</table>

Chatbot 4 (Deepseek V3.2)

التفصيل:

ما معنى "مزمن" في حالة الشقيقة؟

- مزمن في السياق الطبي يعني حالة صحية طويلة الأمد أو متكررة، وليس بالضرورة أن تكون الأعراض مستمرة كل يوم.
- الشقيقة تتميز بنوبات متكررة من الصداع الشديد، تتراوح من عدة مرات في الشهر إلى عدة مرات في السنة، وقد تهدأ لفترات ثم تعود.

أنواع الشقيقة من حيث التكرار:

1. الشقيقة العرضية: وهي الأكثر شيوعًا، حيث تحدث النوبأ أقل من 15 يومًا في الشهر.
2. الشقيقة المزمنة (حسب التعريف الطبي الدقيق): عندما يحدث الصداع لمدة 15 يومًا أو أكثر في الشهر (لمدة 3 أشهر على الأقل)، يكون 8 منها على الأقل ذات خصائ شقيقة. هذا النوع أقل شيوعًا لكنه أكثر إعاقة.

النقاط المهمة:

- لا يوجد علاج جذري يشفي من المرض تمامًا في معظم الحالات، ولكن تُدار الحالة بنجاح كبير.
- الهدف من العلاج: تقليل تكرار النوبات، وشدتها، ومدتها عند حدوثها، وتحسين جودة الحياة.
- مع التقدم الطبي، تتوفر اليوم خيارات علاجية وقائية وعلاجية فعالة جدًا، مثل الأدوية الخاصة بالشقيقة (مضادات CGRP)، والتي غيرت حياة الكثير من المرضى.
- يمكن أن تتغير طبيعة الشقيقة مع الوقت، فقد تتحسن لدى البعض مع التقدم في العمر، أو قد تختفي لفترات طويلة.

الخلاصة:

الشقيقة هي حالة عصبية مزمنة، لكن هذا لا يعني أن المصاب محكوم عليه بمعاناة دائمة بدون تحسن. مع التشخي الصحي، وخطة علاج مناسبة تشمل الأدوية وتعديل نمط الحياة (إدارة المحفزات، النوم، التوتر، النظام الغذائي)، يمكن لمعظم المصابين بالشقيقة إدارة حالتهم بنجاح

<table>
<tr><th colspan="3">Chatbot 4 (Deepseek V3.2)</th></tr>
<tr><td></td><td></td><td>والعيش حياة طبيعية ونشطة.

من المهم استشارة طبيب أعصاب متخص□ في الصداع للحصول على التشخي□ الدقيق وخطة العلاج الشخصية المناسبة لك.</td></tr>
<tr><td>18</td><td>ما مضاعفات الشقيقة؟</td><td>مضاعفات الشقيقة (الصداع النصفي) يمكن أن تتراوح من خفيفة إلى شديدة، وقد تشمل ما يلي:

1. المضاعفات قصيرة المدى أو أثناء النوبة:
• الغثيان والقيء: مما قد يؤدي إلى الجفاف.
• الحساسية الشديدة للضوء والصوت والروائ□: مما يجعل المريض غير قادر على ممارسة الأنشطة اليومية.
• اضطرابات بصرية أو حسية (الهالة): مثل رؤية أضواء وامضة أو نقاط عمياء أو شعور بتنميل في الوجه أو الأطراف (عادةً ما تختفي بعد النوبة).
• الدوخة والدوار: وفي بعض الحالات قد تؤدي إلى فقدان التوازن.
• الصداع المستمر: الذي قد يستمر من 4 ساعات إلى 3 أيام دون علاج فعال.

2. المضاعفات طويلة المدى أو المزمنة:
• تحول الشقيقة إلى صداع يومي مزمن: خاصةً عند الإفراط في استخدام مسكنات الألم.
• التأثير على جودة الحياة: مثل اضطرابات النوم، القلق، الاكتئاب بسبب تكرار النوبات.
• الإعاقة الوظيفية: حيث قد تؤثر النوبات المتكررة على العمل، الدراسة، أو العلاقات الاجتماعية.

3. المضاعفات النادرة والخطيرة:
• السكتة الدماغية (الجلطة): تزداد احتمالية الإصابة بها لدى مرضى الشقيقة المصحوبة بهالة، خاصةً عند التدخين أو استخدام حبوب منع الحمل الهرمونية.
• نوبة الصداع النصفي المستمرة (Status Migrainosus): نوبة شديدة تستمر لأكثر من 72 ساعة وقد تتطلب دخول المستشفى.
• تلف الشبكية: في حالات "الصداع النصفي العيني" النادر الذي قد يؤدي إلى فقدان مؤقت أو دائم للرؤية في عين واحدة.
• متلازمة الأورة المستمرة دون نوبة: حيث تستمر أعراض الهالة (كالرؤية المشوشة) لأكثر</td></tr>
</table>

Chatbot 4 (Deepseek V3.2)		
من أسبوع دون صداع.<br>• المشكلات القلبية الوعائية: قد تزيد الشقيقة من خطر ارتفاع ضغط الدم وأمراض القلب.<br><br>**4. مضاعفات مرتبطة بالعلاج:**<br><br>• الصداع الارتدادي: بسبب الإفراط في استخدام المسكنات (أكثر من 10-15 يومًا شهريًا).<br>• آثار جانبية دوائية: مثل مشاكل في المعدة من المسكنات، أو تعب من الأدوية الوقائية.<br><br>**نصائح للوقاية من المضاعفات:**<br><br>• الالتزام بالعلاج الوقائي الموصوف من الطبيب.<br>• تجنب المثيرات المعروفة (كبعض الأطعمة، التوتر، قلة النوم).<br>• عدم الإفراط في استخدام مسكنات الألم لتجنب الصداع الارتدادي.<br>• المتابعة الدورية مع طبيب الأعصاب خاصةً إذا تغيرت طبيعة النوبات.<br>• التحكم في عوامل الخطر القلبية (مثل التدخين، الضغط، الكوليسترول).<br><br>إذا واجهت أيًا من الأعراض التالية مفاجئة وشديدة مع الصداع النصفي، فاطلب الرعاية الطبية فورًا:<br><br>• صداع حاد جدًا مختلف عن المعتاد.<br>• ضعف أو تنميل مفاجئ في جانب واحد من الجسم.<br>• صعوبة في الكلام أو فقدان البصر.<br>• ارتفاع درجة الحرارة أو تيبس الرقبة.<br><br>العلاج المناسب والمتابعة الطبية يمكن أن تقلل بشكل كبير من هذه المضاعفات وتحسن جودة الحياة.		
مرض الشقيقة (الصداع النصفي) له مسار متفاوت جداً بين الأشخاص، لكن<br>هناك بعض الأنماط العامة المتعلقة بالتقدم في العمر:	هل تتحسن الشقيقة مع التقدم بالعمر؟	19

Chatbot 4 (Deepseek V3.2)

بالنسبة للعديد من المصابين:

- تميل النوبات إلى التحسن مع التقدم في العمر، خاصة بعد سن اليأس لدى النساء. يعتبر ذروة نشاط المرض عادة بين سن 20 و50 سنة.
- قد تقل حدة النوبات وتكرارها مع التقدم في العمر لدى نسبة كبيرة من المرضى.

لكن هذا ليس قانوناً عاماً، وهناك استثناءات وتفاصيل مهمة:

1. اختلاف حسب النوع: قد يتحسن الصداع النصفي مع تقدم العمر، لكن الصداع النصفي المصحوب بهالة (أورة) قد يستمر لفترة أطول في الحياة.
2. تغير في طبيعة النوبات: قد تختلف الأعراض. مثلاً، قد تختفي مرحلة الصداع الشديد لدى كبار السن ويتبقى فقط الأعراض الأخرى (كالدوخة، اضطرابات بصرية، إرهاق) مما قد يجعل التشخي▯ صعباً.
3. استمرارية لدى البعض: نسبة من المرضى (خصوصاً النساء) قد تستمر معاناتهم من النوبات حتى بعد سن الستين.
4. صداع جديد في سن متأخرة: ظهور صداع نصفي جديد لأول مرة بعد سن الخمسين أمر غير معتاد ويستدعي مراجعة طبية فورية لاستبعاد أسباب أخرى.

عوامل تؤثر على المسار:

- التغيرات الهرمونية (مثل انقطاع الطمث) غالباً ما تؤدي إلى تحسن ملحوظ لدى النساء.
- العلاجات الوقائية وتغيير نمط الحياة يمكن أن تساهم في تقليل التكرار والحدة على المدى الطويل.
- الحالات الصحية الأخرى التي تظهر مع التقدم في العمر قد تؤثر على الشقيقة.

خلاصة:

Chatbot 4 (Deepseek V3.2)		
بينما هناك ميل عام نحو التحسن مع التقدم في العمر، خاصة بعد منتصف العمر، إلا أن التجربة تبقى فردية. لا يمكن ضمان اختفاء النوبات تماماً لدى الجميع. الاستمرار في المتابعة مع طبيب أعصاب أو مخت [illegible] أمر ضروري لتعديل الخطة العلاجية بما يتناسب مع كل مرحلة عمرية وللتأكد من أن أي تغيير في طبيعة الصداع ليس عرضاً لمشكلة صحية أخرى.<br><br>إذا كنت تعاني من الشقيقة، فإن التقدم في العمر يعطي أملاً معقولاً بتحسن الأعراض، لكن الإدارة المستمرة للحالة تظل مهمة.		
**علاج نوبات الشقيقة (الصداع النصفي)**<br><br>**العلاجات المتاحة:**<br><br>**1. علاجات الأعراض الحادة (تُؤخذ أثناء النوبة):**<br>• مسكنات بسيطة: باراسيتامول، إيبوبروفين، نابروكسين (يفضل تناولها مبكراً عند بدء الأعراض)<br>• التريبتانات: سوماتريبتان، ريزاتريبتان، إليتريبتان (للنوبات المتوسطة إلى الشديدة)<br>• مشتقات الإرغوت: إرغوتامين، ديهيدروإرغوتامين<br>• مضادات الغثيان: ميتوكلوبراميد، دومبيريدون (غالباً مع المسكنات لتحسين امتصاصها)<br>• مضادات CGRP: ريميجيبانت، أوبروجيبانت (أدوية حديثة)<br><br>**2. العلاجات الوقائية (للوقاية من النوبات المتكررة):**<br>• حاصرات بيتا: بروبرانولول، ميتوبرولول<br>• مضادات الاكتئاب: أميتريبتيلين، فينلافاكسين<br>• مضادات الاختلاج: توبيراميت، فالبروات	ما علاج نوبات الشقيقة؟ و متى يجب أخذ العلاج؟	20

		Chatbot 4 (Deepseek V3.2)
		• حاصرات قنوات الكالسيوم: فلوناريزين<br>• حقن البوتوكس (للشقيقة المزمنة)<br>• مضادات CGRP الوقائية: إرينوماب، جالكانيزوماب (حقن شهرية/ربع سنوية)<br><br>**متى يجب أخذ العلاج؟**<br><br>1. خلال النوبة: يُفضل تناول علاج الأعراض الحادة مبكراً، عند ظهور أولى علامات الصداع أو الأورة البصرية/الحسية.<br>2. العلاج الوقائي: يُوصف عندما:<br>◦ تحدث النوبات 4 مرات شهرياً أو أكثر<br>◦ تدوم النوبات أكثر من 24 ساعة<br>◦ تكون النوبات شديدة وتتعارض مع الحياة اليومية<br>◦ تفشل علاجات النوبة الحادة<br><br>**متى يجب التوجه للطوارئ؟**<br><br>استشر طبيباً فوراً إذا واجهت:<br><br>• صداعاً مفاجئاً شديداً كـ"الرعد"<br>• صداع مصحوب بحمى، تيبس الرقبة، ارتباك، نوبات صرع<br>• ضعف مفاجئ في الأطراف، صعوبة في الكلام<br>• أول نوبة صداع نصفي بعد سن 50<br>• تغيير في نمط الصداع المعتاد<br><br>ملاحظة مهمة: يجب أن يتم التشخيـ والعلاج تحت إشراف طبي، حيث تختلف الخطة العلاجية حسب نوع الشقيقة وشدتها وتكرارها، والأمراض الأخرى المصاحبة. الاستخدام المتكرر لمسكنات الألم (أكثر من 10-15 يوم/شهر) قد يؤدي إلى صداع ارتدادي.
21	ما الأدوية الوقائية لنوبات الشقيقة؟	توجد عدة مجموعات من الأدوية الوقائية التي توصف للتقليل من تكرار نوبات الشقيقة (الصداع النصفي) وشدتها ومدتها. يجب استشارة طبيب أعصاب أو طبيب مختـ لتشخيـ حالتك ووصف الدواء المناسب لك، حيث يعتمد الاختيار على عوامل مثل شدة النوبات، الأعراض المصاحبة، الحالات المرضية الأخرى، والأدوية التي تتناولها.

Chatbot 4 (Deepseek V3.2)		
**المجموعات الدوائية الرئيسية للوقاية من الشقيقة:**<br><br>**1. أدوية خفض ضغط الدم (حتى لو لم يكن المريض مصاباً بارتفاع ضغط الدم)**<br><br>• حاصرات بيتا: مثل بروبرانولول (Propranolol) و ميتوبرولول (Metoprolol). تعتبر غالباً الخط الأول في الوقاية.<br>• حاصلات قنوات الكالسيوم: مثل فيراباميل (Verapamil).<br><br>**2. مضادات الاكتئاب**<br><br>• مضادات الاكتئاب ثلاثية الحلقات: مثل أميتريبتيلين (Amitriptyline) و نورتريبتيلين (Nortriptyline). تُستخدم بجرعات أقل بكثير من تلك المستخدمة لعلاج الاكتئاب.<br>• مثبطات استرداد السيروتونين والنورإبينفرين (SNRIs): مثل فينلافاكسين (Venlafaxine).<br><br>**3. أدوية مضادة للصرع (مضادات الاختلاج)**<br><br>• توبيراميت (Topiramate)<br>• فالبورات الصوديوم (Sodium Valproate) أو دي فالبوريكس (Divalproex sodium). (يجب تجنبها أثناء الحمل).<br><br>**4. أدوية حديثة ومتخصصة للشقيقة**<br><br>• مضادات مستقبلات الببتيد المرتبط بجين الكالسيتونين (CGRP):<br>○ أجسام مضادة وحيدة النسيلة تُعطى بالحقن (مرة شهرياً أو كل 3 أشهر): مثل إرينوماب (Erenumab)، فرينيزوماب (Fremanezumab)، جالكانيزوماب (Galcanezumab)، إبتينيزوماب (Eptinezumab).<br>○ مضادات CGRP الفموية (مستقبلات): مثل ريميجيبانت (Rimegepant) وأتوجيبانت (Atogepant). يستخدم الأخير خصيصاً للوقاية.<br><br>**5. أدوية أخرى**<br><br>• السم العصبي البوتولينيوم (البوتوكس - OnabotulinumtoxinA): معتمد فقط للشقيقة المزمنة (15 يوم صداع فأكثر شهرياً، 8 منها تشبه الشقيقة)، ويعطى كحقن في مناطق محددة من الرأس والرقبة كل 3 أشهر.<br>• بعض حاصرات المستقبلات الأدرينالية: مثل كلونيدين (Clonidine)، لكنها أقل شيوعاً.		

<table>
<tr><th colspan="3">Chatbot 4 (Deepseek V3.2)</th></tr>
<tr><td>
مبادئ مهمة للعلاج الوقائي:

1. الهدف: تقليل وتيرة النوبات بنسبة 50% على الأقل، وليس التخل □ منها تماماً في كل الحالات.
2. التدرج في الجرعة: يبدأ العلاج بجرعة منخفضة ثم تزداد تدريجياً للوصول للجرعة الفعالة.
3. الصبر مطلوب: قد يستغرق ظهور التحسن الكامل من 6 إلى 8 أسابيع أو أكثر من بدء الدواء بالجرعة الفعالة.
4. المتابعة: يجب المتابعة الدورية مع الطبيب لتقييم الاستجابة وتعديل الخطة.
5. الالتزام: الالتزام بالدواء يومياً وفق وصفة الطبيب ضروري للنجاح.
6. العلاج غير الدوائي: جزء أساسي من الخطة الوقائية، ويشمل:
○ تجنب المحفزات الشخصية (كبعض الأطعمة، قلة النوم، الجفاف، التوتر).
○ ممارسة التمارين الرياضية المنتظمة.
○ تقنيات الاسترخاء وإدارة الإجهاد.
○ الانتظام في مواعيد النوم والوجبات.

تحذير هام: لا تبدأ أو توقف أي علاج دوائي بدون استشارة الطبيب. قد يكون لبعض هذه الأدوية آثار جانبية أو تفاعلات مع أدوية أخرى أو موانع استعمال حسب حالتك الصحية.

للتشخي □ الدقيق وخطة علاج وقائي شخصية، يرجى مراجعة طبيب مخت □ في اضطرابات الصداع.
</td><td></td><td></td></tr>
<tr><td>
نعم، يمكن لبعض العلاجات المنزلية والبديلة أن تساعد في التخفيف من أعراض الشقيقة (الصداع النصفي) أو تكرارها، لكنها عادةً لا تكون علاجاً شاملاً بحد ذاتها، خاصة للحالات الشديدة. يجب استشارة الطبيب أولاً لتشخي □ الحالة ووضع خطة علاجية مناسبة.

العلاجات المنزلية والطبيعية الشائعة:

1. الراحة في غرفة مظلمة وهادئة: الضوء والضوضاء قد يفاقمان النوبة.
2. الكمادات الباردة أو الدافئة: وضع كمادة باردة على الجبهة أو الرقبة قد يخفف الألم.
3. الترطيب والنظام الغذائي:
○ شرب الماء بانتظام (الجفاف محفز شائع).
○ تجنب الأطعمة المحفزة مثل (الجبن المعتق، الشوكولاتة، الكافيين الزائد، المواد الحافظة مثل الغلوتامات أحادية الصوديوم).
</td><td>هل يمكن علاج الشقيقة بطرق منزلية أو بديلة؟</td><td>22</td></tr>
</table>

Chatbot 4 (Deepseek V3.2)		
○ تناول وجبات منتظمة لتجنب انخفاض سكر الدم.<br>4. الزيوت العطرية: مثل زيت النعناع (للصداع التوتري المصاحب) أو زيت اللافندر (للاسترخاء)، لكن استخدامها بحذر.<br>5. الأعشاب والمكملات (تحت إشراف طبي):<br>○ الزبدة (البارثينوميد): قد تقلل التكرار عند بعض المرضى.<br>○ المغنيسيوم: نقصه مرتبط بالشقيقة.<br>○ فيتامين 2B (ريبوفلافين).<br>○ الزنجبيل: قد يخفف الغثيان المصاحب.<br><br>**العلاجات البديلة والتكميلية:**<br><br>1. الوخز بالإبر (Acupuncture): أظهرت بعض الدراسات فعالية في تقليل تكرار النوبات.<br>2. التدليك والعلاج الطبيعي: خاصة لصداع التوتر العضلي.<br>3. العلاج السلوكي والتأمل: لتقليل التوتر الذي قد يكون محفزاً.<br>4. اليوجا وتمارين الاسترخاء: لتحسين التنفس وتقليل الإجهاد.<br>5. الارتجاع البيولوجي (Biofeedback): تعلم التحكم في استجابات الجسم مثل توتر العضلات.<br><br>**نقاط مهمة جداً:**<br><br>● التشخي ⍰ الطبي أساسي: الشقيقة قد تكون مشابهة لحالات أخرى تحتاج علاجاً مختلفاً.<br>● العلاجات المنزلية مكملة وليست بديلة: لا تتوقف عن أدويتك الموصوفة دون استشارة الطبيب.<br>● تحديد المحفزات الشخصية: مثل (التوتر، قلة النوم، بعض الأطعمة، التغيرات الهرمونية) يساعد في الوقاية.<br>● الحالات الطارئة: إذا صاحب الصداع أعراض مثل (اضطراب الرؤية المفاجئ، ضعف الأطراف، صعوبة الكلام، ارتفاع حاد في الحرارة)، يجب التوجه للطوارئ فوراً.<br><br>**الخلاصة:**<br><br>العلاجات المنزلية والبديلة يمكن أن تكون جزءاً فعالاً من خطة إدارة الشقيقة، خاصة في التقليل من الاعتماد على المسكنات أو التحكم في المحفزات. لكن يجب دمجها مع نصيحة الطبيب الذي قد يصف أدوية وقائية أو علاجية حسب شدة الحالة.		

<table>
<tr><th colspan="3">Chatbot 4 (Deepseek V3.2)</th></tr>
<tr>
<td>23</td>
<td>ما خيارات علاج الشقيقة أثناء الحمل؟</td>
<td>
إليك نظرة شاملة على خيارات علاج الشقيقة (الصداع النصفي) أثناء الحمل، مع التركيز على السلامة أولاً:

مبدأ أساسي: استشيري طبيبك دائمًا

قبل تناول أي دواء أو تجربة أي علاج، استشيري طبيب النساء والتوليد وطبيب الأعصاب. تُعد الموازنة بين تخفيف ألمك وحماية الجنين أمرًا بالغ الأهمية.

خيارات العلاج أثناء الحمل:

1. العلاج غير الدوائي (الخط الأول والأكثر أمانًا)

• الراحة في غرفة مظلمة وهادئة: تجنب الأضواء الساطعة والأصوات العالية.
• الكمادات الباردة: وضع كمادة باردة على الجبهة أو مؤخرة الرقبة.
• التمارين البسيطة: كالمشي الخفيف أو تمارين الإطالة إذا كان الألم يسم[illegible] بذلك.
• تقنيات الاسترخاء: التأمل، التنفس العميق، اليوغا الآمنة للحمل.
• العلاج بالتدليك: تدليك الرقبة والكتفين.
• تنظيم النوم والوجبات: النوم الكافي، وتناول وجبات منتظمة لتجنب انخفاض السكر.
• تجنب المحفزات: مثل بعض الأطعمة (الشوكولاتة، الجبن القديم، الكافيين بكميات كبيرة)، الروائ[illegible] القوية، والإجهاد.

2. العلاج الدوائي (بعد استشارة الطبيب)
</td>
</tr>
</table>

Chatbot 4 (Deepseek V3.2)

يجب أن يصف الطبيب الدواء المناسب حسب شدة الألم ومرحلة الحمل.

أ. لعلاج نوبات الصداع الحادة:

- باراسيتامول (أسيتامينوفين): يعتبر الخيار الأكثر أمانًا أثناء الحمل لتسكين الألم عند الحاجة ولفترات محدودة.
- مضادات الالتهاب غير الستيرويدية (مثل إيبوبروفين، نابروكسين):
  - قد تكون آمنة في الثلث الثاني فقط من الحمل (بين الأسبوعين 13 و27 تقريبًا).
  - ممنوعة في الثلث الأول (خطر على قلب الجنين) والثلث الثالث (خطر على كلى الجنين وإغلاق مبكر للقناة الشريانية).
- أدوية التريبتان (مثل سوماتريبتان):
  - تظهر معظم الدراسات والأدلة أنها لا ترتبط بزيادة خطر التشوهات الخلقية. يعتبر سوماتريبتان أحد الخيارات المقبولة لبعض الحوامل مع نوبات شديدة لا تستجيب للعلاجات الأخرى.
  - يجب وصفها بدقة من قبل الطبيب المخت [illegible] فقط.

ب. للوقاية من النوبات المتكررة (عند تكرارها أكثر من 3 مرات شهريًا):

قد يوصي الطبيب بأدوية وقائية إذا كانت النوبات شديدة ومتكررة وتعيق حياتك. بعض الخيارات التي قد يناقشها الطبيب:

- حاصرات بيتا (مثل ميتوبرولول، بروبرانولول): تعتبر من الخيارات المقبولة.
- بعض مضادات الاكتئاب (مثل أميتريبتيلين): بجرعات منخفضة أحيانًا.
- المغنيسيوم: له بعض الأدلة على فعاليته في الوقاية وهو آمن عمومًا.

أدوية يجب تجنبها تمامًا أثناء الحمل:

<table>
<tr><th colspan="3">Chatbot 4 (Deepseek V3.2)</th></tr>
<tr><td>

- إرغوتامين وديهيدروإرغوتامين: ممنوعة تمامًا بسبب تأثيرها على انقباضات الرحم وخطر نق □ تدفق الدم للمشيمة.
- حمض الفالبرويك (دواء الصرع والصداع النصفي): يرتبط بزيادة خطر التشوهات الخلقية.

نقاط مهمة جدًا:

1. مرحلة الحمل: يختلف الخيار الدوائي باختلاف الثلث (أول، ثاني، ثالث).
2. الصداع الجديد أو المختلف: إذا ظهر صداع جديد شديد جدًا، مفاجئ، أو مصحوب بزغللة في البصر أو تورم، اطلبي العناية الطبية فورًا لاستبعاد حالات خطيرة مثل تسمم الحمل.
3. الرضاعة الطبيعية: إذا كنت تخططين للإرضاع، ناقشي خطة العلاج مع طبيبك لاستمرار الأمان بعد الولادة.

الخلاصة: البدء بالعلاجات غير الدوائية هو الأساس. عند الحاجة للأدوية، يعتبر الباراسيتامول هو المسكن الأول. للصداع الشديد أو المتكرر، يجب التشاور مع الطبيب المخت □ لتقييم أفضل خطة علاجية شخصية تأخذ بعين الاعتبار فوائد الدواء ومخاطره بالنسبة لك ولجنينك.

أتمنى لك رحلة حمل آمنة ومريحة.

</td><td></td><td></td></tr>
<tr><td>

لا يوجد حاليًا علاج نهائي يشفي من الشقيقة (الصداع النصفي) بشكل تام، حيث إنه حالة عصبية مزمنة. لكن هذا لا يعني أن الوضع ميؤوس منه - يمكن السيطرة على المرض بشكل كبير جدًا.

الهدف من العلاج هو:

1. تقليل عدد النوبات وشدتها بشكل كبير (قد تصل إلى اختفائها لشهور أو سنوات).
2. تقليل مدة النوبة وأعراضها.

</td><td>هل يمكن الشفاء من الشقيقة بشكل نهائي؟</td><td>24</td></tr>
</table>

Chatbot 4 (Deepseek V3.2)

3. تحسين جودة الحياة وجعل الحالة قابلة للإدارة.

الأدوات الرئيسية للتحكم في الشقيقة:

1. العلاج الوقائي (البروفيلاكتيك): أدوية يومية (مثل حاصلات بيتا، مضادات الاختلاج، بعض مضادات الاكتئاب، وأدوية حديثة مثل مضادات CGRP) لتقليل وتيرة النوبات.
2. علاج النوبة الحادة: مسكنات ومضادات التهاب غير ستيرويدية، تريبتانات، إرغوتامينات، وأدوية حديثة مثل جيبانتز.
3. تعديل نمط الحياة: من أكثر العوامل فعالية:
   - تحديد المحفزات وتجنبها: مثل أطعمة معينة (الجبن القديم، الكحول، الكافيين)، التوتر، قلة النوم أو كثرته، التغيرات الهرمونية، الروائ□ القوية، الأضواء الساطعة.
   - الانتظام: في مواعيد النوم والوجبات.
   - التمارين الرياضية المعتدلة والمنتظمة.
   - تقنيات إدارة التوتر: مثل التأمل، اليوغا، التنفس العميق.
4. العلاجات التكميلية: قد تساعد بعض التقنيات مثل الوخز بالإبر (الإبر الصينية)، التدليك، والارتجاع البيولوجي (Biofeedback).
5. حقن البوتوكس: معتمد للشقيقة المزمنة (15 يوم صداع أو أكثر شهريًا).
6. الأجهزة الطبية: أجهزة تحفيز عصبي خارجية (مثل Cefaly) يمكن استخدامها للوقاية أو العلاج.

النقاط المهمة:

- التشخي□ الدقيق من قبل طبيب أعصاب متخص□ في الصداع هو الخطوة الأولى والأهم.
- كل مريض مختلف، وما ينج□ مع شخ□ قد لا ينج□ مع آخر. الأمر يتطلب صبرًا وتعاونًا مع الطبيب لإيجاد الخطة المثلى.
- هناك أبحاث مستمرة لفهم أسباب الشقيقة بشكل أعمق وتطوير علاجات أحدث.

الخلاصة: بينما لا يوجد "شفاء نهائي" بالمعنى التقليدي، يمكن إدارة المرض والسيطرة عليه بشكل ممتاز لدرجة عيش حياة طبيعية إلى حد كبير مع نوبات قليلة جدًا أو خفيفة. المفتاح هو التشخي□ الصحي□، العلاج المناسب، والتزام المريض بخطة العلاج وتعديل نمط الحياة.

يُنص□ دائمًا باستشارة طبيب أعصاب متخص□ في علاج الصداع للحصول على الخطة العلاجية

<table>
<tr><th colspan="3">Chatbot 4 (Deepseek V3.2)</th></tr>
<tr><td></td><td></td><td>الشخصية الأكثر فعالية.</td></tr>
<tr><td>25</td><td>كيف تؤثر الشقيقة على حياة المصابين؟</td><td>الشقيقة (الصداع النصفي) ليست مجرد صداع عادي، بل هي حالة عصبية معقدة تؤثر بشكل عميق على جودة حياة المصابين بها على multiple مستويات:

1. على المستوى الجسدي والألم:
• نوبات ألم شديدة: ألم نابض غالباً في جانب واحد من الرأس، يستمر لساعات أو حتى أيام.
• أعراض مصاحبة: غثيان، قيء، حساسية مفرطة للضوء والصوت والروائ[illegible].
• أعراض قبل النوبة (أورة): اضطرابات بصرية (مثل رؤية flashes من الضوء)، خدر، صعوبة في الكلام لدى بعض المصابين (الصداع النصفي مع أورة).

2. على المستوى الوظيفي والأكاديمي والمهني:
• انخفاض الإنتاجية: خلال النوبة، يصب[illegible] أداء أي عمل مستحيلاً تقريباً.
• غياب متكرر: الكثيرون مجبرون على التغيب عن العمل أو المدرسة.
• صعوبة التخطيط: الخوف من حدوث نوبة فجأة يعطل القدرة على الالتزام بالمواعيد أو وضع خطط مستقبلية.
• تأثير مالي: خسارة أيام العمل قد تؤدي لخسارة الدخل، بالإضافة لتكاليف العلاج.

3. على المستوى النفسي والعاطفي:
• القلق والتوتر المستمر: العيش في خوف دائم من موعد النوبة القادمة.</td></tr>
</table>

Chatbot 4 (Deepseek V3.2)

- الاكتئاب: الألم المزمن والعزلة الاجتماعية تزيد من خطر الاكتئاب.
- الإحباط والشعور بالعجز: خاصة عندما لا يفهم المحيطون خطورة الحالة ويعتبرونها "مجرد صداع".

4. على المستوى الاجتماعي والعائلي:

- العزلة الاجتماعية: تجنب المناسبات، الحفلات، الأماكن المزدحمة أو المضيئة خوفاً من تحفيز نوبة.
- تأثير على العلاقات: الصعوبة في الوفاء بالالتزامات العائلية أو الاجتماعية، مما قد يؤدي إلى توتر العلاقات.
- اعتمادية: قد يصب[illegible] المريض معتمداً على المساعدة من أفراد العائلة أثناء النوبات.

5. على المستوى اليومي والروتيني:

- تقييد الأنشطة: تجنب ممارسة الرياضة أو بعض الأطعمة أو العوامل المحفزة المعروفة.
- اضطراب النوم: الألم قد يمنع النوم، وقلة النوم قد تسبب نوبة (دورة مفرغة).
- عبء الإدارة اليومية: الالتزام بتناول الأدوية، تتبع مذكرات النوبات، وتعديل نمط الحياة باستمرار.

6. التحديات غير المرئية:

الشقيقة مرض "غير مرئي" غالباً، مما يؤدي إلى:

- قلة التفهم: المجتمع قد يقلل من معاناة المصاب.
- وصمة العار: اتهام المصابين بالمبالغة أو عدم التحمل.

<table>
<tr><th colspan="3">Chatbot 4 (Deepseek V3.2)</th></tr>
<tr><td>

كيف يمكن إدارة التأثير؟

- التشخي□ الدقيق وخطة علاجية: تحت إشراف طبيب أعصاب (قد تشمل أدوية وقائية ومسكنة).
- تعديل نمط الحياة: تنظيم النوم، التغذية، الرياضة الخفيفة، إدارة التوتر.
- التعليم والدعم: فهم المرض يساعد في تقليل الخوف. الانضمام لمجموعات الدعم مفيد جداً.
- التواصل: شرح الحالة للأهل والأصدقاء ورب العمل لخلق بيئة داعمة.

خلاصة القول: تأثير الشقيقة يكون شاملاً وطويل الأمد، ويمتد إلى أبعد من لحظة النوبة نفسها. الفهم المتزايد للمرض كحالة عصبية وليس مجرد صداع، والدعم المناسب، يمكن أن يخفف بشكل كبير من هذا العبء ويحسن جودة حياة المصابين بشكل ملحوظ.

</td><td></td><td></td></tr>
</table>
